# Corrosion of Metallic Biomaterials: A Review

**DOI:** 10.3390/ma12030407

**Published:** 2019-01-28

**Authors:** Noam Eliaz

**Affiliations:** Department of Materials Science and Engineering, Tel-Aviv University, Ramat Aviv 6997801, Israel; neliaz@tau.ac.il; Tel.: +972-3-6407384

**Keywords:** biomaterials, biocompatibility, corrosion, failure, titanium alloys, stainless steels, shape memory alloys, biodegradable metals, metallic glasses, body environment

## Abstract

Metallic biomaterials are used in medical devices in humans more than any other family of materials. The corrosion resistance of an implant material affects its functionality and durability and is a prime factor governing biocompatibility. The fundamental paradigm of metallic biomaterials, except biodegradable metals, has been “the more corrosion resistant, the more biocompatible.” The body environment is harsh and raises several challenges with respect to corrosion control. In this invited review paper, the body environment is analysed in detail and the possible effects of the corrosion of different biomaterials on biocompatibility are discussed. Then, the kinetics of corrosion, passivity, its breakdown and regeneration in vivo are conferred. Next, the mostly used metallic biomaterials and their corrosion performance are reviewed. These biomaterials include stainless steels, cobalt-chromium alloys, titanium and its alloys, Nitinol shape memory alloy, dental amalgams, gold, metallic glasses and biodegradable metals. Then, the principles of implant failure, retrieval and failure analysis are highlighted, followed by description of the most common corrosion processes in vivo. Finally, approaches to control the corrosion of metallic biomaterials are highlighted.

## 1. Introduction

Biomaterials are commonly defined as nonviable materials intended to interface with biological systems to evaluate, treat, augment or replace any tissue, organ or function of the body [1]. Before a new biomaterial is introduced to the market, various issues are considered, including its designated anatomic location, functional tissue structure and pathobiology, mechanical and other property requirements, toxicology, biocompatibility, the healing process, ethics, standardization and regulation [2]. Material, device and procedure standards are issued by international organizations, mainly the International Standards Organization (ISO) and The American Society for Testing and Materials (ASTM). To preclude ineffectually tested devices and materials from coming on market and to filter entities clearly unqualified to produce biomaterials, regulatory systems have been established by both the USA and the European Union. While the assessment in the USA is by a government agency (i.e., the U.S. Food & Drug Administration, FDA), in Europe it is by Notified Bodies (NBs). While the focus of the latter is primarily on proof of safety, the former puts significant emphasis also on effectiveness of a device. An essential European requirement for marketing is first obtaining a CE Marking. It should be noted that the FDA does not regulate the materials used in medical devices but rather the devices themselves.

Biocompatibility is an essential requirement of a biomaterial. A biocompatible material performs with an appropriate host response (i.e., minimum disruption of normal body function) in a specific application [3]. Thus, the material causes no thrombogenic, toxic or allergic inflammatory response when it is placed in vivo. There are two key factors determining the biocompatibility of a material: the host reactions induced by the material, and the degradation of the material in the body environment. Often, both factors should be considered.

Since about 4000 years ago, humans have been using artificial materials to repair fractured and diseased tissues and organs. In the early ages, the Greeks and Egyptians implanted wood and bones from animals in humans. The development of advanced biomaterials is related to the development of modern medicine and advanced materials. Only in 1546 was a synthetic material (gold plate) used to repair a cleft palate. Vanadium steel was developed in the early 1900s specifically for implants [4]. Its first application was bone fracture fixation plates introduced by Sherman and aimed at stabilizing bone fractures and accelerating their healing. Quickly, however, implant dysfunctionality due to corrosion, mechanical failure and poor biocompatibility was reported. In 1924, Zierold [5] reported the effect of various metals on the surrounding tissues. When inserted to bone, copper and nickel caused significant discoloration of the surrounding tissue, while iron and steel dissolved rapidly and aggravated tissue erosion. Although certain pure metals such as gold, silver and aluminium did not cause tissue discoloration, there were too soft for most medical devices. In 1926, the 18Cr–8Ni (wt%) stainless steel was first used in implants. This steel was both more corrosion resistant in vivo and stronger than the vanadium steel. Later that year, molybdenum was added to the steel to improve its corrosion resistance in chloride-containing water. This alloy is known as 316 stainless steel. In 1940, titanium and its alloys were first considered for orthopedic practice. These materials had been used in aircraft applications and showed excellent corrosion resistance in seawater. Therefore, good corrosion resistance in vivo could be anticipated. This was indeed observed after implant retrieval. In 1947, Maurice Down introduced a variety of orthopedic devices such as plates and screws made of titanium. In the 1950s, the 316L stainless steel was introduced. The carbon content in this alloy was reduced from 0.08 wt% to 0.03 wt% in order to improve the corrosion (sensitization) resistance and weldability compared to 316 stainless steel. In the 1960s, Sir John Charnley, the British orthopedic surgeon, introduced the first successful total hip replacement (THR) in patients suffering from osteoarthritis (OA). The damaged femoral head was removed and the hip replaced with a stainless steel ball and a high-density polyethylene (HDPE) socket. Methacrylate bone cement was used for implant fixation. This may be regarded as the beginning of modern orthopedics, in which the development of novel materials plays a central role. THR is one of the most successful and cost-effective operations in the whole of medicine. Charnley’s operation has improved the quality of life of millions of humans by relieving pain of hips with arthritis and avascular necrosis, restoring mobility and correcting deformity. 

**Figure 1:**
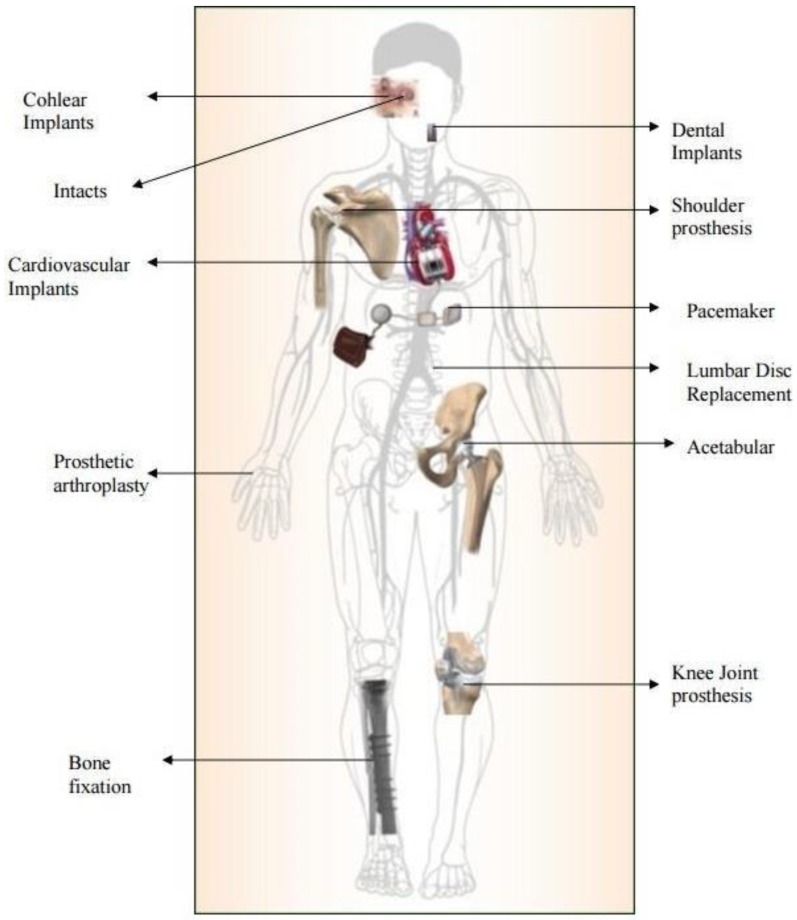
Application of metallic biomaterials as implants in different areas of the human body [39]. Reproduced with permission from Bentham Science Publishers.

Nowadays, biomaterials are made of metals and alloys, ceramics, polymers and composites. Figure 1 illustrates some applications of metallic biomaterials. One example is the vascular stents made of stainless steel or shape memory alloy (SMA), sometimes coated with a polymer for drug eluting [6]. The global coronary stents market size was estimated at USD 9.3 billion (milliard) in 2016 and is expected to reach USD 15.2 billion by 2024. Another example is the use of calcium phosphate (CaP) bioceramics in the field of bone regeneration, both in orthopedics and in dentistry [7,8,9,10,11,12,13,14,15,16,17,18,19,20,21,22,23,24,25,26,27,28,29,30,31,32]. CaPs are common in the form of coatings on titanium implants, but they are also used as scaffolds, bone fillers and cements.

Corrosion is an important factor in the design and selection of metals and alloys for service in vivo. Allergenic, toxic/cytotoxic or carcinogenic (e.g., Ni, Co, Cr, V, Al) species may be released to the body during corrosion processes. In addition, various corrosion mechanisms can lead to implant loosening and failure [33,34,35,36,37,38,39]. Therefore, biomaterials are often required to be tested for corrosion and/or solubility before they are approved by regulatory organizations. Hence, the corrosion behaviour of metallic implant materials has been widely studied, in the framework of quality assurance, implant retrieval analysis and failure analysis.

The objective of this review paper is to introduce the reader with the fundamentals of biomaterials corrosion. First, the body environment is described in Section 2. As shown, this environment is harsh and puts several challenges with respect to corrosion control. Subsequently, the principles of biocompatibility are presented in Section 3, because this term is often used in corrosion-related reports. Next, Section 4 discusses the kinetics of corrosion, including passivity, its breakdown and regeneration. Section 5 presents the major metals and alloys currently used in biomedical applications. A brief discussion of implant failure, retrieval and failure analysis is given in Section 6. Section 7 then reviews the most important corrosion mechanisms in vivo. Finally, Section 8 focuses on strategies for corrosion control in vivo.

## 2. The Body Environment

The body environment and its effects on corrosion have been reviewed in many manuscripts (see, for example, [32,38,39,40,41,42,43,44,45]). The water content of the human body ranges from 40% to 60% of its total mass. Functionally, the total body water can be subdivided into two major fluid compartments, namely the extracellular and the intracellular fluids. Extracellular fluids (ECFs) consist of the plasma found in the blood vessels, the interstitial fluid that surrounds the cells, the lymph and transcellular fluids (e.g., cerebrospinal fluid and joint fluids). Intracellular fluid (ICF) refers to the water inside the cells. Both the amount and the distribution of body fluids and electrolytes are kept normal and constant, a mechanism known as homeostasis.

Electrolytes play a major role in body functionality. Among various functions, they take part in metabolism, determine the cell membrane potentials and osmolarity of body fluids and so forth. Major cations include hydrogen, sodium, potassium, calcium and magnesium ions. Major anions include hydroxide, bicarbonate, chloride, phosphate and sulphate ions. Dissolved salts are probably the most influential components for implant corrosion in vivo. Chloride ions (and other halides) enhance the corrosion of almost all metals and interfere with many methods of corrosion protection. 

Temperature and pH are two important factors affecting the corrosion behaviour of materials. Under normal conditions, body fluids have a temperature of 37 °C. This can be regarded as a constant temperature throughout the lifespan of an implant with respect to corrosion. Reference [46] provides thorough definition and discussion of different terms, reactions and procedures in electrochemistry. Hence, these will not be discussed in this article, except when very important for the understanding of the following sections.

The hydrogen evolution reaction (HER) and oxygen evolution reaction (OER) are two important reduction reactions in corrosion in general, and also in vivo. The HER in acid solutions is

(1) $$2H^{+}+2e^{-}\to H_{2},$$

whereas in alkaline solutions it is written as

(2) $$2H_{2}O+2e^{-}\to H_{2}+2{\left(OH\right)}^{-}.$$

The Nernst equation for the HER is

(3) $$E_{\mathrm{rev}}=\frac{2.3RT}{2F}\log\frac{{\left[H^{+}\right]}^{2}}{\left[H_{2}\right]}=-\frac{2.3RT}{2F}\log\left[H_{2}\right]-\frac{2.3RT}{F}\mathrm{pH}.$$

The OER in neutral or acidic solutions can be written as

(4) $$O_{2}+4H^{+}+4e^{-}\to 2H_{2}O,$$

whereas in alkaline solutions it is

(5) $$4{\left(OH\right)}^{-}\to O_{2}+4e^{-}+2H_{2}O.$$

The corresponding Nernst equation is

(6) $$E_{\mathrm{rev}}=+1.229+\frac{2.3RT}{4F}\log\left[O_{2}\right]-\frac{2.3RT}{F}\mathrm{pH},$$

where [H_2_] and [O_2_] represent the partial pressures of hydrogen and oxygen, respectively.

In addition to the above HER and OER reduction reactions, the following reactions, Equations (7) through (10), are some other possible reduction reactions occurring at implant surfaces. Hydrogen peroxide (H_2_O_2_) and hydroxide radicals ($HO_{2}^{•}$ and $OH^{•}$) participate in these reactions. It is thus evident that certain intermediate species, which are known to considerably affect the biological system and prompt oxidative stress in cells, might result from reduction processes [40]. There are many other possible reduction reactions in vivo, including reduction of disulfide bonds and other protein-like molecules. The local redox environment around a metallic implant can influence the redox state of cells [40].

(7) $$O_{2}+2H_{2}O+2e^{-}\to H_{2}O_{2}+2{\left(OH\right)}^{-},$$

(8) $$H_{2}O_{2}+2e^{-}\to 2{\left(OH\right)}^{-},$$

(9) $$O_{2}+H_{2}O+e^{-}\to HO_{2}^{•}+{\left(OH\right)}^{-},$$

(10) $$HO_{2}^{•}+H_{2}O\to H_{2}O_{2}+OH^{•}.$$

Neutrality is defined when [H^+^] = [OH^–^]. Thus, pH = 7 for neutrality at 25 °C. However, because the equilibrium constant (ionic product) is a function of temperature, the pH at neutrality depends on temperature too. For example, *K*_w_ = 2.4 × 10^–14^ (mol L^–1^)^2^ at 37 °C, thus the corresponding pH value at neutrality in the human body will be 6.81. The acid-base balance is an important part of homeostasis: metabolism depends on enzymes and enzymes are sensitive to pH. The normal pH range for blood plasma is 7.35 to 7.45. A decrease in blood pH below normal is known as acidosis, whereas an increase in blood pH above normal is known as alkalosis. Buffers resist changes in pH. There are two major types of mechanisms that control the body pH: chemical and physiological. The rapid-acting chemical buffers (e.g., bicarbonate, phosphate and protein systems) immediately (i.e., in fractions of a second) combine with any added acid or alkali that enters the body fluids, thus preventing drastic changes in hydrogen ion concentration and pH. If the immediate action of chemical buffers cannot stabilize the pH, the physiological buffers (i.e., respiratory and urinary response systems) serve as a secondary defence against harmful shifts in pH. The physiological buffer system controls the output of acids, bases or CO_2_. The respiratory response system buffers within minutes, whereas the urinary response system requires several hours, but it buffers the greatest quantity [47].

When an implant is placed in vivo, the disruption of blood supply to the bone is often accompanied by severe pathological infections that might affect the healing and cause electrochemical variations in the equilibrium state [48]. In addition, the pH of the body fluid can drop from 7.4 to 5.5 and it could take 10 to 15 days to recover its normal value. Bacterial infection could result in even a wider range of pH in vicinity of the implant surface, from acidic to alkaline (4.0 to 9.0, respectively). Laing [49] reported that the pH around a newly inserted implant can drop to as low as 4.0 due to the build-up of hematomas, a condition that could last for several weeks. The local decrease in pH could result in severe localized corrosion of the metal implant. In addition, H_2_O_2_ may form during the initial stages of the inflammatory response to the placement of an implant in vivo [50,51]. The level of the aforementioned pathological changes depends on the biological activity of any corrosion products released from the implant as well as on the implant size and shape; it could vary across the surface of the implant, possibly leading to the development of electrochemical cells [52]. The risk of localized corrosion due to local variations in pH in vicinity of titanium alloys has also been reported based on in vitro studies [53].

The most important characteristics of body fluids that influence the corrosion of metal implants are the chloride, dissolved oxygen and pH levels. Body fluids may seem to be slightly less aggressive than seawater, based on the lower pitting resistance equivalent number (PREN) of 26 and greater recommended to prevent pitting corrosion of stainless steels in vivo, in comparison to the value of 40 usually required for stagnant seawater [54]. However, the dissolved oxygen levels in blood are lower than in artificial solutions exposed to air atmosphere due to combination with haemoglobin, which is the main component of red blood cells. The partial pressure of oxygen in blood varies between 100 to 40 mmHg for arterial and venous blood, respectively. The corresponding value in air is 160 mmHg. Because most biomaterials rely on oxygen to repassivate, repassivation of metal surfaces is more difficult under conditions of low dissolved oxygen concentration. Indeed, deaeration of the solution with high-purity nitrogen gas to maintain low O_2_ concentration was found to better predict the in vivo performance of metal implants [55]. Since the partial pressure of oxygen varies widely within the body, from about 2.67 × 10^2^ to 1.33 × 10^4^ Pa, an implant surface can be in contact with anatomical environments of widely different oxygen partial pressures, thus possibly establishing aeration cells. Another gas, carbon dioxide (CO_2_), influences the corrosion in vivo by affecting the pH [41]. Bicarbonate levels are about twenty times higher in blood than in seawater [56].

A very useful method of describing the stability of metals in aqueous solutions is the potential-pH (Pourbaix) diagrams introduced by Marcel Pourbaix [57,58]. Black [44] was probably the first one to draw the Pourbaix diagram for body fluids. This diagram illustrates the range and complexity of conditions that may be experienced by biomaterials in vivo. The OER line is the upper limit of water stability; it represents oxygen-rich solutions or electrolytes near oxidizing materials. In the human body–saliva, intracellular fluid and interstitial fluid are saturated with oxygen and their stability domains are therefore near the oxygen evolution line. The HER line is the lower limit of water stability. In the human body–urine, bile, the lower gastrointestinal tract and the secretions of ductless glands have stability domains somewhat above the hydrogen evolution line. Aqueous corrosion can occur in the region between these two lines (i.e., in the water stability domain). It is evident from the Pourbaix diagram of body fluids that different pH values and oxygen concentrations prevail in different parts of the body. Consequently, a metal that performs well in one part of the body, namely it is either immune or passive, might undergo an unacceptable extent of corrosion in another part. 

The Pourbaix diagrams have some limitations [46]. Since these are equilibrium diagrams, we can only learn from them what cannot happen. We cannot deduce which reaction will happen at a measurable rate, because these diagrams do not reflect the kinetics. The fact that at a certain pH and potential a metal can corrode according to its Pourbaix diagram is no proof that it actually will do so. Whether passivation will or will not form depends on the nature of the oxide and on the environment in contact with it. Furthermore, potential-pH diagrams are usually given for the pure elements, while many metallic biomaterials are alloys. The corrosion behaviour of an alloy is rarely, if ever, a linear combination of the corrosion behaviour of its components. Even for a given composition, the corrosion of an alloy usually depends on metallurgical factors such as the grain size and heat treatment of the material. An extreme example is the high corrosion resistance of some of the so-called glassy metals or amorphous alloys, compared to crystalline alloys of the same composition. Other limitations of the Pourbaix diagrams: (1) they typically refer to pure water, but in body fluids there are other ions that may affect equilibria. (2) Their shape is affected by the species that are taken into account, thus previous knowledge of the specific system is required. (3) The pH is that in vicinity of the metal surface, which could differ significantly from the pH of the bulk solution.

Dissolved oxygen, dissolved bicarbonate and some other constituents of body fluids (e.g., phosphates, cholesterols and phospholipids) are usually thought to either play no role in the corrosion process or exist at insignificant levels. Therefore, most in vitro experiments have been conducted in either saline or standard isotonic solutions such as Ringer’s or Hank’s, in which the presence of bicarbonate and calcium chloride is the main difference compared to saline. Blood has been used in some corrosion studies, often adding sodium citrate to the blood as an anticoagulant. Nevertheless, sodium citrate has been shown to affect the passivation behaviour of Co-Cr-Mo alloys and stainless steels, among others [45]. Some studies have avoided the use of anticoagulants by performing the tests in blood serum [45]. Compositions of selected body fluids and simulated body fluids (SBFs) are provided in Table 1 and Table 2, respectively. Phosphate buffered saline (PBS) is mostly recommended because it maintains the pH almost constant throughout in vitro experiments [59]. A review by Solar [60] concluded that inorganic solutions based on diluted NaCl were indeed satisfactory substitutes for human body fluids when studying the behaviour of passive metals. Thus, many researchers use the simple saline solution (0.9 wt% NaCl in DI water) for in vitro experiments. However, some differences between in vivo and in vitro corrosion evaluation have been reported, which have been attributed, among others, to the higher concentration of dissolved oxygen in isotonic solutions compared to venous blood [56,61]. In addition, accelerated in vivo corrosion may be associated with some minor constituents in blood. For example, sulphur contained in amino acids may enhance crevice corrosion of stainless steels [62]. Another cause of difference between corrosion data acquired in vivo versus in the laboratory stems from different hydrodynamic conditions affecting the implant surface. For example, blood flow can cause mass transport-limited reactions to take place at noticeably different rates than assessed in the lab.

With respect to dental implants, the environment in the oral cavity is not well-defined [36,42]. Several recipes exist for artificial saliva, the most popular one is that of Fusayama [63], that is, 0.400 g·dm^–3^ NaCl, 0.400 g·dm^–3^ KCl, 0.795 g·dm^–3^ CaCl_2_·H_2_O, 0.690 g·dm^–3^ NaH_2_PO_4_·H_2_O and 0.005 g·dm^–3^ Na_2_S·9H_2_O, at pH 5.5. However, the composition of human saliva actually varies considerably between individuals, especially in the sulfide content, which can cause tarnishing of both silver- and gold-based amalgams. Many foodstuffs are acidic and have high chloride content; thus, they are significantly more corrosive than saliva. In addition, oral hygiene has a strong effect on the corrosiveness of the oral environment. Finally, many dental products and solutions contain fluoride, which is harmful to the passive layer. Some of the special varnishes used by dentists, for example, contain more than 2 wt% fluoride [64]. Thus, although dental implants are relatively accessible for repair or replacement, there has been a concern that the toxicity of metals leaching out from amalgams, Nitinol and other dental materials, might cause oral cancer [65,66].

### 2.1. The Effect of Biological Macromolecules

Biological macromolecules can change the corrosion rate by interfering in different ways with the anodic or cathodic reactions. Proteins and lipids from the ECF adsorb onto the implant surface and may changes its chemical properties through oxidation and/or hydrolytic reactions. 

Many articles report the effects of *proteins* on the corrosion behaviour of different biomaterials, yet the conclusions are ambiguous. This is probably due to varying experimental procedures (e.g., which proteins were used) but also because the effect of a specific protein varies on different metals and alloys [38,41]. Proteins may have several effects on the corrosion behaviour: (1) Proteins can bind to metal ions and transport them away from the implant surface. This will destabilize the equilibrium across the electrical double layer (EDL) and trigger further dissolution of the metal. (2) Proteins can affect the electrode potential due to their electron-carrying capability, whereas bacteria can change the pH of the local environment by generation of acidic metabolic products. (3) The adsorption of proteins onto the surface of biomaterials could limit the diffusion of oxygen to certain regions of the surface, thus causing preferential corrosion of oxygen-deficient regions and breakdown of the passive layer [2]. (4) An adsorbed protein layer could act as a barrier between the metal surface and the environment, thus inhibiting corrosion. (5) In the case of wear or wear-assisted corrosion reactions, proteins can act as lubricants on the surface. The procedures used to study protein effects on metals vary greatly, in that either serum (containing many proteins) or single proteins (most frequently–albumin, being the most abundant protein in the blood) are added [41].

*Cells* can significantly influence the nature of the passive films and the corrosion behaviour of metallic biomaterials [67]. However, the effects observed depend on the type of metal/alloy studied. As typically the cell-material interactions are cell-specific, it is not possible to draw a general conclusion on the effect of cells on the corrosion behaviour [38,41]. Similar to the discussion in the last paragraph, different effects of cells on the corrosion behaviour could be expected when they adhere on surfaces: (1) the cell layer could act as a physical barrier, blocking the surface and thus increasing its corrosion resistance. (2) Cells may release strong oxidizing agents and enzymes that are targeted at decomposing the implant material. Cell metabolism products could influence the surface reactions. For instance, macrophages can generate active oxygen species (O_2_^−^, for example), which can lead to increased metal release from titanium in the absence of wear and fretting [41].

*Bacteria* near an implant could consume hydrogen that is released in cathodic reactions, thus accelerating the corrosion process [2].

### 2.2. The Effects of Relative Motion and Crevices

The relative motion between the surfaces of an implant and a tissue might accelerate wear of both surfaces, thus stimulating chronic inflammation and creating an even severer chemical environment [8,68,69]. Some implants inherently introduce crevices with local solution chemistry that is significantly different than the physiological environment of 0.154 M saline at pH 7.4. Examples include modular tapers and metal-on-metal (MoM) hip replacements. Analysis of retrieved implants has indicated that the pH within taper crevices could be lower than 1 and that cation concentrations within tapers could be orders of magnitude higher than in the peri-implant tissues [40].

### 2.3. Correlations Between In Vitro and In Vivo Tests

Corrosion of metals in vivo may be regarded as an electrochemical process. While the electromotive force (EMF) series lists metals according to their thermodynamic driving force to undergo oxidation, the practical nobility of metals in vivo may be different either because: (1) the biomaterial is an alloy rather than a pure metal, (2) the pressure-concentration-temperature conditions are not standard, or (3) the implant is surrounded by serum ions, proteins and cells, which may all affect local corrosion processes. Hence, significantly different corrosion behaviours have been documented in non-physiological in vitro tests, physiological in vitro tests, and in vivo studies. Since all metals corrode to some extent in vivo, one should refer to corrosion control rather than to corrosion prevention, and international standards of metallic implants and medical devices typically include a requirement for corrosion tests. After implantation, raised metal concentrations are often measured even in remote organs. This may be related to ionization but also to phagocytosing cells that circulate small metal and metal oxide particles [40].

Kuhn et al. [70] reviewed the literature for comparisons between in vitro and in vivo corrosion data. It was concluded that:

(1) Certain organic species, notably serum, accelerate the corrosion rate in vivo of some metals and alloys under non-fretting (static) conditions. Such species should be included in artificial test environments for a better correlation with the in vivo corrosion rates of these metals.
(2) The use of electrochemical test methods for corrosion testing, especially in the presence of complex organic species, simple sulfides and other possibly electrochemically active compounds, should be avoided if such compounds, by their anodic oxidation, were shown to interfere with the measurement. The importance of this is probably greatest with noble metals and less with passivating metals.
(3) Given the slowness of the passivation process for certain metals and alloys, the value of short-term (i.e., less than 1,000 h) experiments is questionable for these materials.
(4) The use of electrochemical techniques conducted in simple electrolytes, such as PBS, afford a valid means of ranking or screening biomaterials, subject to comments 1 to 3 above.
(5) The importance of correct specimen mounting to avoid undesired crevice formation and the role of solution agitation effects should be borne in mind.
(6) The formation in vivo of a soft or hard tissue capsule around an implant may retard corrosion. It should be possible to simulate this in laboratory corrosion tests by application of some sort of sheath around the test specimen.

## 3. Biocompatibility

A biocompatible material disrupts the normal body function as little as possible. There are many factors which influence implant biocompatibility; for example, implant size, shape, material composition, surface wettability, surface roughness and charge. The biomaterial must not: (1) change plasma proteins (including enzymes) so as to activate adverse reactions; (2) cause thrombus formation, adverse immune response or cancer; (3) destroy or sensitize the cellular elements of blood; (4) produce toxic or allergic responses; (5) deplete electrolytes; (6) be affected by sterilization. In turn, the environment should not cause degradation (e.g., biological or mechanical) or corrosion of the biomaterial that would result in loss of physical and mechanical properties. In practice, no synthetic material is completely harmonious with the living environment; however, materials do have different levels of inertness.

Following implantation of biomaterials and medical devices, the following sequence of local events takes place: injury, blood-material interactions, provisional matrix formation, acute inflammation, chronic inflammation, granulation tissue development, foreign-body reaction (FBR) and fibrosis (fibrous capsule development) [71]. The initial inflammatory response is activated by injury to vascularized connective tissue. Since blood and its components are involved in the initial inflammatory responses, blood clot formation and/or thrombosis also occur. Acute inflammation is of relatively short duration, lasting from minutes to days, depending on the extent of injury. Its main characteristics are the exudation of fluid and plasma proteins and the emigration of leukocytes. Chronic inflammation is less uniform histologically than the acute inflammation. In general, it is characterized by the presence of mononuclear cells (i.e., monocytes and lymphocytes) at the implant site, with the proliferation of blood vessels and connective tissue. Chronic inflammation is typically of short duration and is confined to the implant site. The persistence of the acute and/or inflammatory responses beyond a three-week period usually indicates an infection. After the acute and chronic inflammatory responses are resolved, granulation tissue can be identified by the presence of macrophages, the infiltration of fibroblasts and neovascularization in the new healing tissue. Depending on the degree of injury, granulation tissue may be seen as early as 3–5 days post-implantation. The essence of FBR (a special form of nonimmune inflammation) is that the presence of an implant changes the healing process. FBR involves protein adsorption, macrophages, multinucleated foreign body giant cells (i.e., fused macrophages), fibroblasts and angiogenesis. FBR can result in fibrosis, scar or even biomaterial rejection. The FBR with the development of granulation tissue is considered the normal wound healing response to implanted biomaterials. For most inert biomaterials, the late tissue reaction is encapsulation by a relatively thin fibrous tissue, composed of collagen and fibroblasts. The cellular components of the FBR separate this fibrous capsule from the granulation tissue.

Obviously, a major factor influencing the host reaction to a biomaterial is the surface of the latter, because it is the one that the body “senses” first. The specific reactions at the surface determine the FBR, the path and speed of the healing process and the long-term development of the biomaterial/body interface. The chemical composition, structure and morphology of a surface are all important in this regard. Hence, the nature of the initial interface that is established between an artificial material and the adjacent tissue determines the ultimate success or failure of the implant.

*Osteolysis* (i.e., active resorption of bone matrix) and the formation of a thick fibrous layer between the implant and bone are indicative of poor biocompatibility. Furthermore, normally non-toxic materials (e.g., polyethylene) might trigger an inflammatory response when they are in the form of microparticles of certain size. These particles cause an irritation of phagocytic cells and activate them to produce and release cytokines, proteinases, growth factors and other proinflammatory factors, eventually leading to chronic inflammation, fibrosis, osteolysis and porosis in bone. In the case of *aseptic loosening* of the prosthesis, the presence of wear particles could lead to the formation of a poorly vascularized, synovial-like interface membrane between the prosthesis and bone. The formation of necrotic focuses, granulomas and osteolysis may finally result in loosening of the prosthesis. The increase of metallic wear also increases the total surface are of the metallic biomaterial, and consequently the concentration of metal ions. The porous surface increases the surface area but also particular wear.

The corrosion of metallic implants can affect the surrounding tissues in three ways: (i) electrical current may affect the behaviour of cells, (ii) the corrosion process may change the chemical environment, and (iii) metal ions may affect cellular metabolism [72,73]. One of the issues that arise from the release of corrosion products into the body is systemic and remote effects [8,40,69,72,73,74,75]. Mild corrosion in many cases can also produce symptoms, which range from a local tenderness at the site of the corroded area to acute pain, reddening and swelling over the whole general area around the device. Systemic reaction to metallic implants stem from this flux of metal ions released by corrosion. Organ specific accumulations of certain metal ions together with the simultaneous ion specific excretion rates from the body could lead to the establishment of elevated concentrations of specific alloying elements of implants. This could upset the overall balance established by physiological tolerance to toxicity [73,74]. In animals and patients with either stainless steel or cobalt-base orthopedic total joint replacement (TJR) components, corrosion and wear produce longer-term changes in blood composition, primarily in its metal content. These include elevations of metallic content in tissue (at both local and remote sites, e.g., kidney and liver) and of metal-bearing ion concentrations in serum and urine. Although histology has not provided a clear proof of the slow release of metal ions, the discoloration of the surrounding tissue and the FBRs evidently point on implant corrosion as the origin of these metal ions [76]. 

*Metallosis* is a local destruction of soft and hard tissues due to metal implants [8,43,69,75]. It is often associated with significant osteolysis and is sometimes reflected by an infiltration of periprosthetic soft tissues and bone by metallic debris resulting from wear of joint arthroplasties [43]. By themselves, metal ions lack the structural complexity required to challenge the immune system. However, when combined with proteins, such as those available in the skin, connective tissues and blood, a wide variety of metals induce immune responses and thus should be considered harmful. Cobalt, chromium and nickel are the most common examples, nickel having the strongest effect. Delayed type IV hypersensitivity is the most typical response of a metal-sensitized individual to such metal ions [69,75]. Table 3 lists the adverse effects of some metals. 

**Figure 2:**
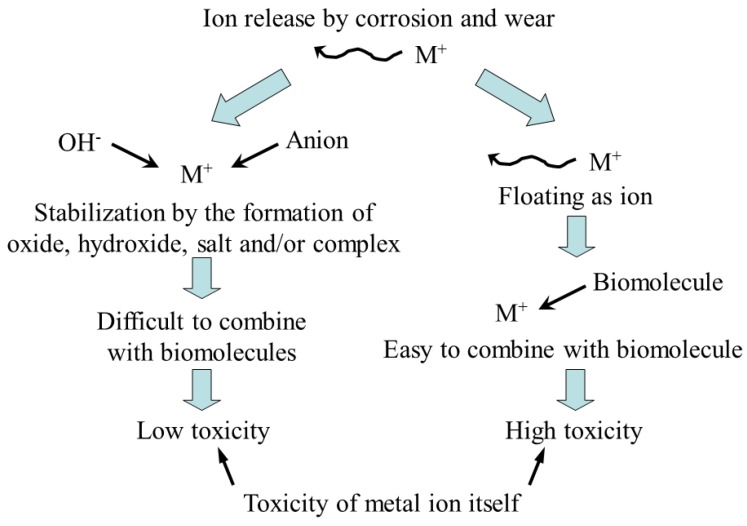
Two possible toxicity routes for metal ions released into body fluids due to corrosion and wear [42]. Reprinted with permission from Springer Nature.

Metal ions released into the human body do not always harm it; the partner for combination with them is very important (Figure 2). Whether an ion will be active and immediately react with water molecules or inorganic anions depends on the number and mass of the molecules. For example, titanium ion is very active and readily reacts with hydroxyl radicals and anions, forming oxide and salt in body fluids, thus indicating that the possibility of combination with biomolecules is low. However, this does not mean that the reaction of titanium ions with biomolecules will not occur at all. Zirconium, niobium and tantalum ions behave like titanium ions. On the other hand, inactive ions, for example, of nickel and copper, do not immediately combine with water molecules and inorganic anions and last in the ionic state for relatively long time. Therefore, these ions have higher chance of combining with biomolecules and exhibit toxicity. To conclude, the following factors must be considered when dealing with toxicity of metallic biomaterials: (1) corrosion resistance of the material, (2) ions released by corrosion and wear, (3) activity of the released ions, and (4) toxicity of the ion itself [42].

**Figure 3:**
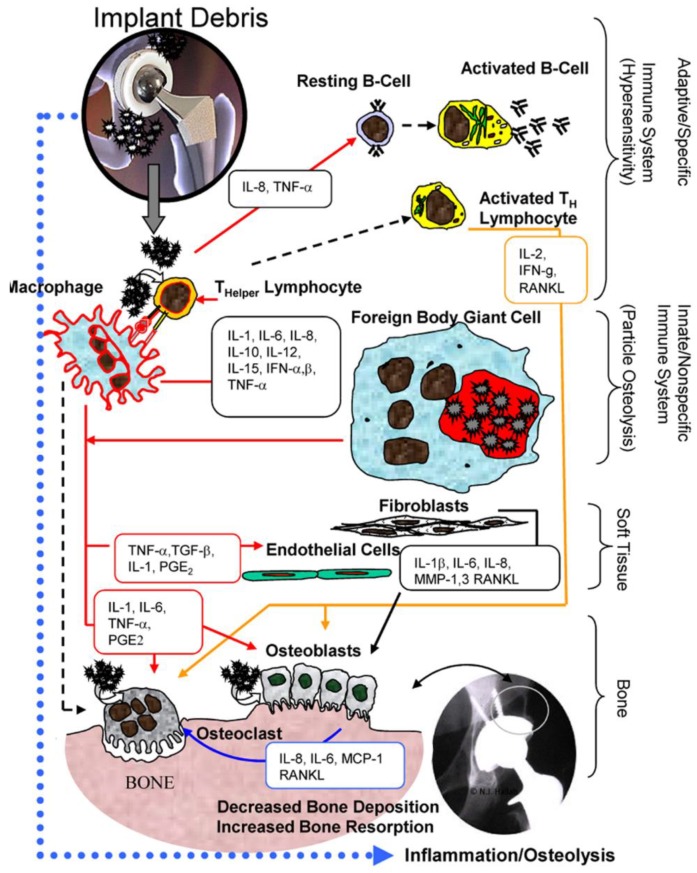
The biologic reactivity of implant debris causes local immune responses primarily mediated by macrophages, which produce reactive oxygen intermediates and pro-inflammatory cytokines that affect a host of local cell types and induce a widening zone of soft-tissue damage and inflammation [69]. Reprinted with permission from Springer Nature.

Figure 3 [69] illustrates that the biologic reactivity of implant debris causes local immune responses primarily mediated by macrophages, which produce reactive oxygen intermediates and pro-inflammatory cytokines that affect a host of local cell types and induce a widening zone of soft-tissue damage and inflammation.

While corrosion *per se* may not be of great concern, when combined with mechanical effects, restricted crevice-like geometries, inflammation or any combination thereof, considerably amplified corrosion rates might arise, potentially leading to adverse host response or implant failure [40].

In contrast to the case of metals, immune responses to biopolymers have not been reliably reported, whereas immune responses to bioceramics are unlikely thanks to their exceptionally low solubility [75,77,78].

### Cells, Tissues and Wear Particle Analysis

The act of rupture due to wear is localized in a small volume of material, which is removed in the form of wear particles (i.e., wear debris). Different wear mechanisms generate different wear particles with different sizes, shapes, surface morphologies and colours [80]. Isolation and analysis of wear debris, cells and tissue fragments from body fluids is valuable in disease diagnosis and prognosis, remnant life prediction, failure analysis and optimization of implants.

*Ferrography* is a non-destructive condition monitoring technique, which has been found very sensitive and successful in monitoring the wear state of engineering systems. In analytical ferrography, ferromagnetic and paramagnetic particles suspended in a flowing liquid are captured on a glass slide thanks to the interaction between their magnetic moments and a strong external magnetic field. By quantifying the ferrographic patterns (i.e., number, size, shape, texture and colour) and determining the composition of different particles on the ferrogram–the origin, mechanism and level of wear can be determined [80]. The success of *analytical ferrography* in condition monitoring of engineering systems triggered few feasibility studies in the fields of life sciences and medicine, mainly in hip and knee joint applications, already in the 1980s [81]. These included erythrocyte and white blood cell separation [82,83,84,85], bacterial tracking [84,85,86] and monitoring the wear of either natural diarthrodial joints [81,87,88,89,90,91,92,93,94,95,96] or artificial joints [87,88,91,97]. In those studies, Er^3+^ was the magnetizing agent.

A modified version of the conventional analytical ferrograph, known as Bio-Ferrograph, was specifically developed for magnetic isolation of target cells or tissues [80]. Since its introduction in the late 1990s, it has been used in several feasibility studies aimed at tracking Escherichia coli bacteria in natural watersheds [98,99,100,101,102,103,104,105], isolation and characterization of low concentrations of Vibrio cholera bacteria from a ship’s ballast water [106], capture of magnetic minerals embedded in the comb cells of *Vespinae* [107] and isolation of carbon nanoparticles [108]. Bio-Ferrography has also been used to isolate cancer cells, with very promising results [80,81,109,110,111,112,113]. In addition, it has been used for isolation of bone and cartilage particles from synovial fluids for early diagnosis of osteoarthritis [81,114,115] and isolation of both polymeric and metallic wear particles from synovial fluids for either design or failure analysis of artificial hip joints [80,81,116,117,118,119]. 

**Figure 4:**
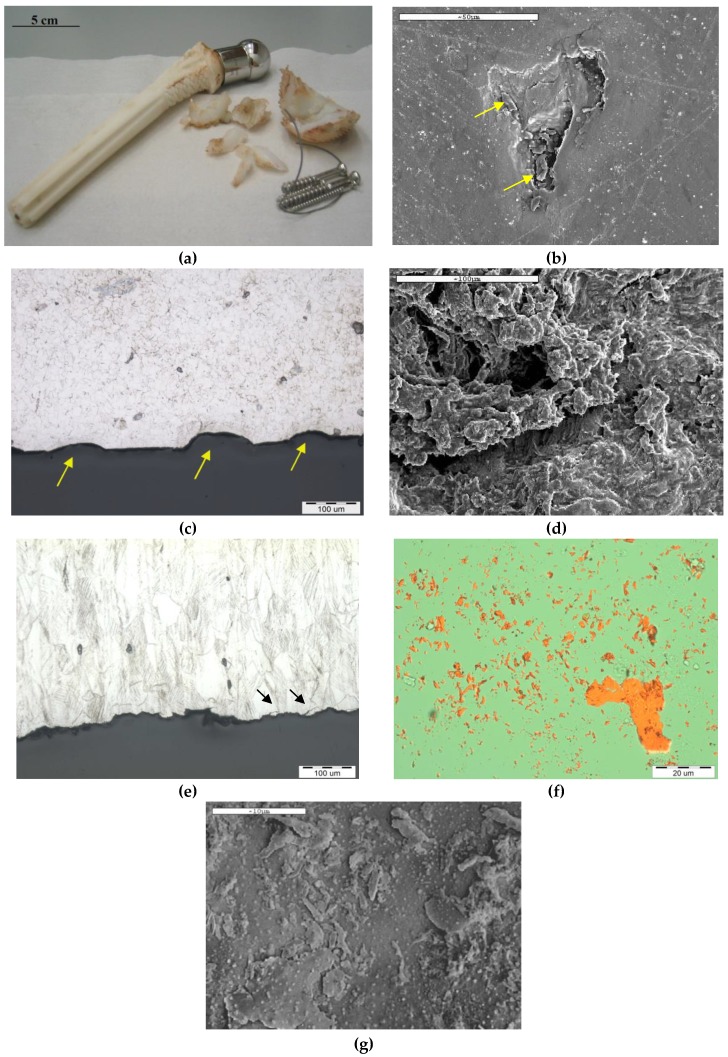
The use of Bio-Ferrography in the study of biodegradation of artificial hip joint. (**a**) A retrieved cementless isoelastic hip joint. (**b**,**c**) SEM and optical microscope images of pits on the neck surface. (**d,e**) Transgranular SCC of a stainless steel screw. (**f,g**) Isolated 316L stainless steel particles as seen under an optical microscope with bichromatic illumination (**f**) and by SEM (**f**).

At Tel-Aviv University, we have used Bio-Ferrography to isolate particles suspended in synovial fluids for evaluation of artificial hip and knee joints performance. Synovial fluid aspirates and prosthesis compartments removed by revision surgery from 14 patients were analysed. Results showed that metallic (namely, Ti-, Co- and Fe-based alloys), polymeric (ultrahigh molecular weight polyethylene—UHMWPE, polyoxymethylene—POM, and poly(methyl methacrylate)—PMMA) and bone particles were suspended in synovial fluids. The formation of metal, PMMA and bone particles seemed to further accelerate the wear of certain prostheses. Figure 4a provides a macroscopic view of a failed hip prosthesis. This type of cementless isoelastic prosthesis was designed to reduce stress shielding of the proximal femur. The stem is made of POM, the acetabular cup of UHMWPE, and the ball and neck component from 316L stainless steel. In addition, four bone screws made of 316L stainless steel and a wire made of Ti–6Al–4V are noticed in Figure 4a. One of the screws fractured in vivo. Failure analysis revealed ductile tearing of the UHMWPE, as well as crazing and microvoid coalescence in the POM component. Pitting and wear were noticed in the neck component (Figure 4b,c). Energy dispersive X-ray spectroscopy (EDS) analysis revealed traces of chloride in these pits. Transgranular stress corrosion cracking (TG-SCC) and wear were identified around the fracture surface of the failed screw (Figure 4d,e). The exposure of grain boundaries to the outer surface of the screw indicates that the threads were fabricated by machining and not by plastic forming. This could have made them susceptible to failures by mechanisms such as SCC and fatigue [120]. Surprisingly, FDA guidelines do not include a requirement for manufacturing of threads of bone screws by cold forming, although this is a common knowledge and requirement for critical aircraft screws, for example. Numerous metallic wear particles were observed on the ferrogram under an optical microscope with bichromatic illumination (Figure 4f). Scanning electron microscope (SEM)/EDS analysis showed that 316L stainless steel, POM and bone particles were all suspended in the synovial fluid. The stainless steel particles were mainly in the form of platelets with a smooth surface and no striation marks (Figure 4g). Thus, the failure of the implant was attributed to the synergistic effect of corrosion and wear. This high amount of wear particles suspended in the synovial fluid of an alloy containing both Ni and Cr could alert on the issues of biocompatibility concerned with the use of such a metallic biomaterial. This example demonstrates the potential attractiveness of Bio-Ferrography in the study of implant degradation in vivo.

Another example for biocompatibility-related issues that may arise from the use of biomaterials is given here, this time focusing on the adverse effects of polymer wear debris. Soft bearing materials that aim to reproduce the tribological function of the natural joint are gaining popularity as alternatives to traditional hard bearing materials such as polyethylene, metals and ceramics in artificial hip and knee joints. Soft bearing materials may reduce wear by maintaining a fluid film between the articulating surfaces [80]. A commercial hip system based on a polycarbonate-urethane (PCU) acetabular liner and CoCr femoral head has been available on the European market for several years. 

**Figure 5:**
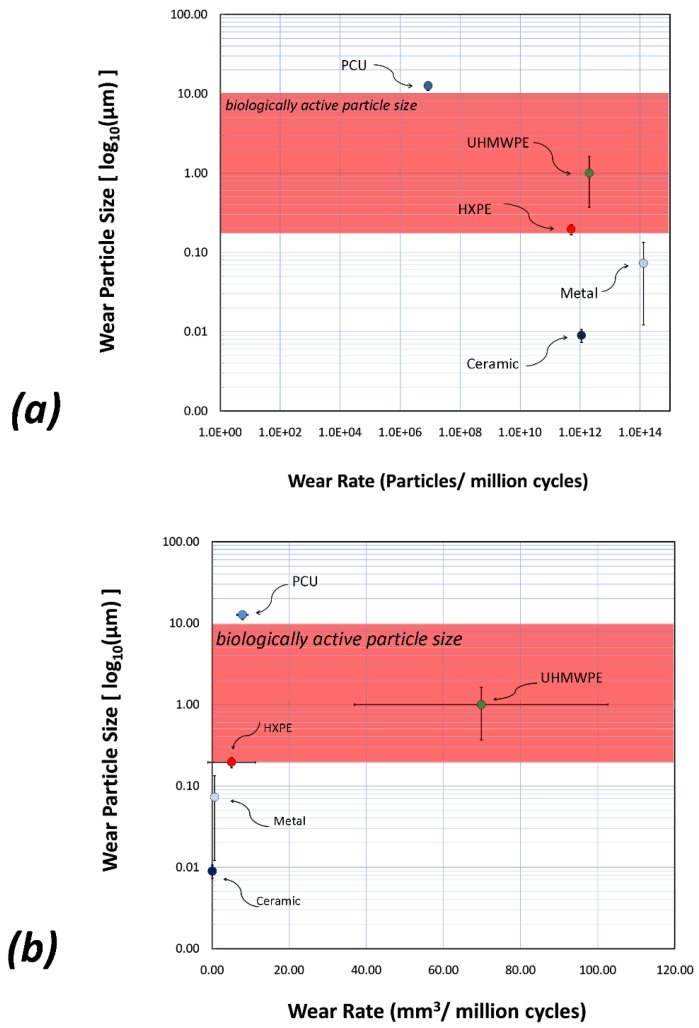
Comparison between typical wear particle sizes versus wear rates reported for various acetabular liners used in total hip replacement (THR). Data is presented based on the volumetric wear rate (**a**) and wear particle generation rate (**b**) [118]. Reprinted with permission from John Wiley & Sons, Inc.

We have analysed by Bio-Ferrography and several microscopy techniques the wear rate and characteristics of PCU bearing coupled to a CoCr femoral head in an artificial hip joint that was run on a multiaxis simulator up to 20 million gait cycles, which may be considered as equivalent to 20 years of clinical use in an average patient [117,118,119]. The PCU liner exhibited a low particle generation rate, with the majority of wear particles lying above the *biologically active range* of polymeric particles (0.2–10 µm), which is regarded as osteolysis inducer, see Figure 5. The steady-state volumetric wear rate of the PCU liner (5.8–7.7 mm^3^ per million cycles) is substantially lower than that of traditional UHMWPE bearings, and of the same order of magnitude as new-generation UHMWPE, highly cross-linked polyethylene (HXLPE) and MoM bearings, see Figure 5b. The PCU liner also showed low particle generation rate (2–3 × 10^6^ particles per million cycles), which is 5, 6 and 6–8 orders of magnitude lower than that of UHMWPE, HXLPE and MoM bearings, respectively (Figure 5a). It was thus anticipated that the combination of larger wear particles, less reactivity and lower particle generation rate would make PCU of lower osteolytic risk compared to hard bearings in THR.

## 4. Kinetics of Corrosion and Passivity

### 4.1. Fundamental Terms and Equations

Thermodynamics can provide only the negative answers; it allows us to calculate and determine which reactions *will not* happen. We need kinetic information to determine what will happen at a rate that may be of practical interest or at least at a rate that can be detected. 

The Transition-State Theory can be used to derive the Butler-Volmer equation. This equation is valid only for activation-controlled reactions. A plot of the current-potential relation is commonly referred to as a *polarization curve*. Two limiting cases (approximations) of the Butler-Volmer equation yield two important equations for corrosion tests: Tafel extrapolation and polarization resistance [46]. The latter is used in *linear polarization resistance* (LPR) measurements [121,122]. 

The understanding gained by considering the Evans diagrams allows us to measure the corrosion current directly. First, we must realize that the corrosion potential is in fact the open-circuit potential (OCP) of a system undergoing corrosion. It represents steady state but not equilibrium. It resembles the reversible potential in that it can be very stable. Following a small perturbation, the system will return to the open-circuit corrosion potential, just as it returns to the reversible potential. It differs from the equilibrium potential in that it does not follow the Nernst equation for any redox couple and there is both a net oxidation of one species and a net reduction of another [46].

The corrosion potential, *E*_corr_, is the potential of a corroding surface in an electrolyte, relative to a reference electrode. It is deduced either from the plateau in the potential transient when the working electrode is not polarized or from Tafel extrapolation of the anodic and cathodic curves in potentiodynamic polarization curves. The current density at the corrosion potential, *j*_corr_, is also deduced from potentiodynamic polarization curves and is directly proportional to the corrosion rate. The higher *E*_corr_ and the lower *j*_corr_ are, the better the corrosion performance of the material is [46].

**Figure 6:**
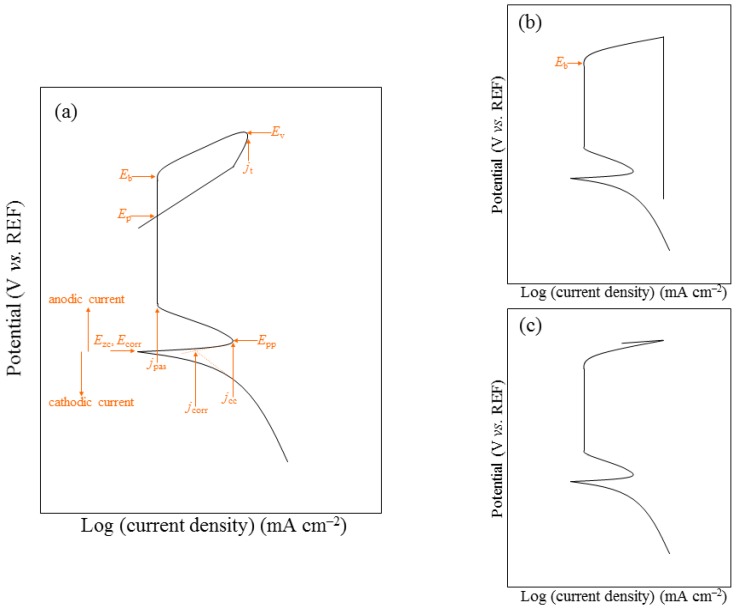
Schematics of cyclic potentiodynamic polarization curves of: (**a**) a metal that exhibits a protection potential, (**b**) a metal that does not exhibit a protection potential and (**c**) a metal that repassivates.

In electrochemical passivation, as the potential of a metal sample is increased in the anodic direction, the rate of dissolution increases, reaches a maximum and then decreases to a very low value. Further increase of the potential has little effect on the current in the passive region until passivity breaks down, whereupon the current rises rapidly with potential. The sequence of events observed on an iron electrode when its potential is swept very slowly in the positive direction is shown schematically in Figure 6a. 

Cyclic potentiodynamic polarization and Tafel extrapolation measurements are described in different ASTM standards [55,122,123,124]. The most common procedure for testing susceptibility of small implants, in their final form and finish, to localized corrosion is described in ASTM F2129 [55]. This procedure is based upon construction of *potentiodynamic polarization curves*, after monitoring the OCP for 1 h. In this technique, the potential of the test specimen is controlled and the corrosion current measured by a potentiostat. The potential is first scanned in the positive (forward) direction until a predetermined potential (or current density), usually within the transpassive region, is reached. Then, in the case of *cyclic potentiodynamic polarization*, the scan is reversed until the specimen repassivates or the potential reaches a pre-set value [46]. The parameters defined in these experiments are identified in Figure 6a. The vertex potential, *E*_v_, is a pre-set potential at which the scan direction is reversed. Similarly, the threshold current density, *j*_th_, is a pre-set current density at which the scan direction is reversed. The corrosion potential and corrosion current density are determined through Tafel extrapolation of the straight-line portion. The ASTM standard defines the zero current potential, *E*_zc_, as the potential at which the current reaches a minimum during the forward scan. The potential at which the anodic dissolution current has its maximum value is called the primary passivation potential, *E*_pp_. The corresponding maximum anodic current is referred to as the critical corrosion current, *j*_cc_. In the passive region, which may extend over half a volt or more, the current density *j*_pas_ is nearly constant (although, very often, metastable pits are apparent by transient bursts of anodic current); this current density is proportional to the charge transferred under passivation conditions. The breakdown (or critical pitting) potential, *E*_b_, is the least noble potential at which pitting or crevice corrosion or both will initiate and propagate, along with oxygen evolution and electrochemical dissolution of the passive film–the occurrence of the latter two depends on the conductivity of the oxide. The anodic process taking place at such high potentials usually involves the transformation of the oxide to a higher oxidation state, which is often more soluble. In contrast, on valve metals (Al, Hf, Nb, Ta, Ti, Zr) the oxide continues to grow in thickness as the potential is increased. If there are no aggressive anions in solution, this anodizing process can lead to very thick oxide films, up to tens of micrometres, on which oxygen evolution cannot occur.

A higher value of *E*_b_ reflects a higher pitting corrosion resistance. Another important parameter is the so-called *protection potential*, *E*_p_, sometimes referred to as repassivation potential, at which the reverse scan intersects the forward scan at a value that is less positive (noble) than *E*_b_. The protection potential cannot be determined if there is no breakdown (see Figure 6c). The absence of a hysteresis loop indicates repassivation or oxygen evolution. While pitting will occur on a pit-free surface only above *E*_b_, it will occur on a pitted surface only in the range of potentials between *E*_p_ and *E*_b_. The susceptibility to crevice corrosion increases as the difference between *E*_b_ and *E*_p_ increases, that is, the hysteresis of the polarization curve becomes larger. Therefore, a higher value of *E*_p_ indicates a higher resistance to crevice corrosion. If the metal does not repassivate until a potential below *E*_r_ is reached, then it is very susceptible to crevice corrosion [46].

It is worth noting that the scan rate (typically, 0.167 or 1 mV s^–1^) may affect the *E*_b_ value and the shape of the passive region. In addition, deaeration of the solution with nitrogen gas before and during the test is recommended. It is also recommended to run experiments under the same conditions on reference devices that have a history of good corrosion resistance in vivo, for comparison. To avoid intensive hydrogen absorption during the cathodic portion of the curve, in particular on hydride-forming metals such as titanium, it is required to start the polarization only 100 mV below *E*_r_ [46].

One of the unique features of most corroding metals undergoing passivation is a region of apparent negative resistance. Looking at Figure 6, we note that at potentials anodic to the primary passivation potential *E*_pp_, the current density decreases with increasing anodic potential until it reaches the passive region. This is an unstable region in which the current keeps decreasing with time, even at constant potential, due to the formation and growth of the passive film [46]. 

In the presence of oxidizing species (such as dissolved oxygen), some metals and alloys passivate spontaneously. Consequently, no active region (and no *E*_pp_, *j*_cc_) is evident in the polarization curve. The oxidizer adds an additional cathodic reaction to the Evans diagram, thus shifting the intersection of the total anodic and total cathodic lines to the passive region (i.e., *E*_corr_ > *E*_pp_). The open-circuit dissolution rate under these conditions is *j*_pas_, which is often on the order of 0.1 μA cm^–2^ or less [46].

Titanium and its alloys typically exhibit a fairly noble OCP or corrosion potential and they are transformed directly into a stable passive behaviour from the Tafel region, without exhibiting an active-to-passive transition. Both commercially pure titanium (CP-Ti) and its alloys typically do not show a breakdown potential or pitting potential in the range of potentials tested (even at potentials higher than *E*_rev_ for the OER). This implies that the oxide layer on titanium is very protective, thus preventing corrosion. Consequently, the release of ionic or by-product residue into the periprosthetic tissue is minimal and these biomaterials may be classified as biologically inert or electrochemically passive in the whole range of clinically relevant potential-pH combinations [40].

**Figure 7:**
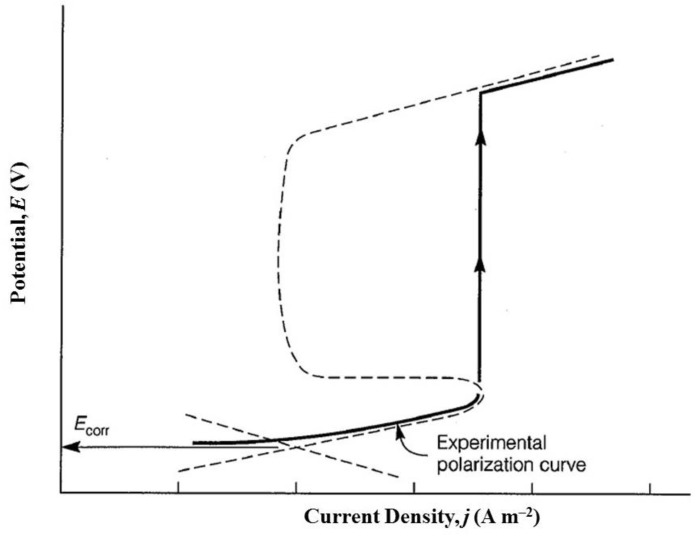
Comparison between potentiostatic (dashed line) and galvanostatic (bold line) anodic polarization curves. The x-axis is in logarithmic scale.

Anodic polarization of active-passive metals and alloys can be established either potentiostatically or galvanostatically. Figure 7 illustrates the differences in the shape of the polarization curves in each case. A detailed study of the important parameters influencing passivity is possible only by potentiostatic measurement. In contrast, because potential is not a single-valued function of current, galvanostatic measurements are inadequate for obtaining the active-passive behaviour properly. Above *j*_cc_, the curve no longer follows the anodic curve in the passive region; instead, it unexpectedly enters the transpassive region where oxygen evolution takes place.

### 4.2. Corrosion Rate

The amount of metal removed by corrosion can be determined from Faraday’s Law:

(11) $$w=\frac{j_{\mathrm{corr}}AtM}{nF},$$

where *w* is the corroded mass (g), *M* is the atomic mass of the metal (g mol^–1^), *t* is time (s) and *A* is the exposed surface area (cm^2^). Possible causes of deviation from Faraday’s Law include: (1) consumption of some charge by parasitic processes; (2) not all of the reactants are consumed; (3) the postulated process is not the actual process; and (4) some of the material from the sample falls of [46].

The corrosion intensity (CI) can be similarly expressed in units of g cm^–2^ s^–1^ as

(12) $$CI=\frac{j_{\mathrm{corr}}M}{nF}.$$

We can also express the *corrosion penetration rate* (CPR) in units of loss-in-dimension perpendicular to the corroding surface per unit time (mil per year (mpy) where 1 mil = 0.001 in = 25.4 μm)

(13) $$CPR=\frac{j_{\mathrm{corr}}}{25.4F\rho }\times \frac{M}{n}\times 3.154\times 10^{11}.$$

In this equation, the density *ρ* is substituted in units of g cm^–3^. The time coefficient takes into account 365 days per year. If we multiple Equation (13) by 25.4 we get the CPR in units of micrometres per year. In Equation (13) *M*/*n* was separated because it is the equivalent weight (EW). The number of equivalents, *N*_EQ_, is simply

(14) $$N_{\mathrm{EQ}}=\frac{1}{EW}=\sum \left(\frac{f_{i}}{M_{i}/n_{i}}\right)=\sum \left(\frac{f_{i}n_{i}}{M_{i}}\right),$$

where *f*_i_ is the mass fraction of element *i* in the alloy. This allows us to calculate the corrosion rate of common alloys [122]. 

**Figure 8:**
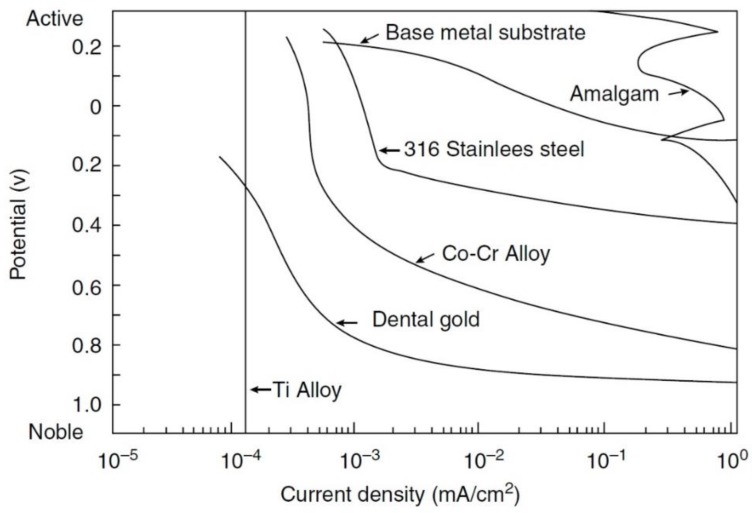
Polarization curves for some biomaterials. Reprinted from Greener et al. [125].

What is an acceptable corrosion rate? This would depend on the application, the possible effect of corrosion on the functionality of the part, safety concerns and the cost of the material. For example, for medical implants we usually permit less than 1 μm per year (0.0394 mpy). Comparison between the typical potential-current density curves for some biomaterials such as titanium alloy, dental gold, Co-Cr alloy, 316 stainless steel and amalgam is shown in Figure 8 [125].

Note that the above corrosion rate value and equations assume uniform (general) corrosion. However, if localized corrosion is involved (as often occurs in vivo), these calculations become less relevant and one should consider other parameters, for example, pit density and depth.

### 4.3. Passivation and its Breakdown

The first and most fundamental step in corrosion is the oxidation of the metal to its lowest stable valence state. This is most often followed by the formation of insoluble products, the exact nature of which depends on the metal and on the environment in which it is corroding. The ion formed in the initial step may be oxidized further, producing oxides or other compounds of mixed valence. In some cases (e.g., Al, Ti, Cr) the corrosion products form dense oxide layers, which prevent further corrosion. In other cases (e.g., low-carbon steel), the layer is porous, allowing the corrosion process to continue until the whole piece of metal has been consumed. When a protective layer does exist, it does not have to be very thick: about 5 nm in the case of Al and 3 nm in the case of stainless steel. Thus, as little as 10–20 molecular layers of the oxide film can provide excellent protection for long periods of time [46]. This oxide layer may be amorphous or crystalline. Calcium and phosphorous contaminations are often observed [74]. Table 4, drawn based on information in Ref. [126], summarizes the characteristics of selected oxide layers on biomaterials.

Chemical passivation was discovered more than 200 years ago. In 1790 James Keir discovered that treatment of iron with nitric acid produces a peculiar condition in which the metal loses its power of precipitating silver from silver nitrate, although retaining its ‘metallic splendour.’ It was Christian Friedrich Schönbein who coined the word ‘passivity’ in 1836, but it was Michael Faraday who brought the phenomenon to the forefront of electrochemistry, also in 1836. Faraday developed the theory according to which passivity was established by an oxide film or by a similar state of affairs. The presence of several nanometres thick oxide film on the surface of passivated metals was confirmed experimentally in the 1960s [46]. 

Uhlig and Revie [127] defined two types of passivity: (1) A marked anodic polarization (i.e., low corrosion rate, noble potential) causes the metal to substantially resist corrosion in a given environment. Examples include Ni, Cr, Ti, Fe in oxidizing environments (e.g., chemical passivating solutions such as chromate), stainless steels and many others; (2) A marked thermodynamic tendency to react (i.e., low corrosion rate, active potential), yet the metal substantially resists corrosion. Examples include Pb in H_2_SO_4_ and Fe in an inhibited pickling acid [46].

Passive films formed in aqueous solutions usually consist of an oxide or a mixture of oxides, usually in hydrated form. Passive layers are electronic insulators or semiconductors depending on the bandgap of their constituents. Valve metals and even pure Sn may be anodized to potentials much above 1.5 V without oxygen evolution, some like Al or Ta even to much more than 100 V. Other metals like Fe, Co, Ni, Cr, Cu and Ag form semiconducting oxides with a sufficiently small bandgap to allow electron transfer reactions with redox systems, including oxygen evolution. Some are even metallic conductors (e.g., IrO_2_, RuO_2_ and PbO_2_). Nickel, chromium and their alloys with iron (notably, the various kinds of stainless steel) can be readily passivated and, in fact, tend to be spontaneously passivated upon contact with water or moist air. It should be noted that passivation does not occur when chloride ions are introduced into the solution and a pre-existing passive film may be destroyed [46]. 

Electrochemical passivation (see Figure 6) is in many ways similar to chemical passivation. Deaeration of the solution with nitrogen gas before and during the test is a common practice to better reflect the reduced oxygen levels in vivo. The values of *E*_pp_ and *E*_b_ as well as the shape of the passive region depend to some extent on the sweep rate. The environment and alloy composition both affect the anodic polarization part. Increasing the chloride (or some other halides) concentration in the environment will shift the curve to the right and will gradually reduce *E*_b_ and the whole passivity region. Increasing the temperature will have a similar effect. Increasing the chromium concentration in the alloy will shift the curve to the left and increase *E*_b_ and the passivity region. Increasing the molybdenum concentration in the alloy will just extend the passivity region to higher potentials, thus improving the resistance of the alloy to localized corrosion [46].

Many theories of metal passivity have been presented in the literature. The major theories are the oxide film theory and the adsorption theory. According to the former, the passive film is always a diffusion-barrier layer of reaction products that separate the metal from its environment and reduces the reaction rate. The electric field across the film drives the following processes: (1) entry of metal atoms into the film as cations at the metal/film interface; (2) transport of the metal cations or of oxygen anions through the oxide; and (3) dissolution of metal cations from the film at the film/environment interface. The adsorption theory, on the other hand, suggests that chemisorbed films displace the normally adsorbed water molecules and reduce the rate of anodic dissolution associated with hydration of metal ions. In 1946, Uhlig proposed that an adsorbed oxygen film is the primary source of passivity. Such a film forms preferentially on the transition metals in accordance with their uncoupled *d*-electrons interacting with oxygen to form a stable bond, combined with their high heats of sublimation favouring retention of metal atoms in their lattice in preference to their removal to form an oxide lattice. Uhlig proposed that a film of adsorbed oxygen atoms markedly decreases the exchange current density in the Tafel equation and hence increases anodic polarization in accordance with the requirements of passive behaviour. For non-transition metals with filled *d*-levels, such as Cu or Zn, the heats of oxygen adsorption are expected to be lower and the formation of oxides is less favourable. Such metals do not exhibit thin-film passivity [46].

The principal paradigm of biocompatibility of metallic biomaterials has been “the more corrosion resistant, the more biocompatible.” Therefore, the majority of metals and alloys used today in implants and other medical devices are characterized by high corrosion resistance. The latter could be attained either due to low thermodynamic driving force for corrosion, as in the case of noble metals (e.g., Au, Ag and Pt) or due to a passive metal-oxide thin film that spontaneously forms on the surface and acts as a kinetic barrier (as in the case of stainless steels, Co-Cr, Ti, Zr, Nb and Ta alloys). However, since the latter have high driving force to corrode, if the oxide film on the surface is ruptured or interrupted–oxidation of the underlying metal will occur, releasing ions into the environment. This will happen until the oxide film regenerates, possibly within milliseconds. Hence, any discussion of biomaterial corrosion should take into account the synergistic effects of electrochemistry, mechanics and biology [40].

When the surface oxide film is degraded mechanically and the underlying bare metal is brought into contact with a body fluid, the following cascade of events will occur. Within the first nanosecond or two, the metal atoms at the surface begin to oxidize and are either released to the solution as ions or remain adsorbed at the metal surface as oxidized ions. Electrons leave the atoms and accumulate at the metal surface, thus forming a strong electric field that can be as high as 10^7^ V cm^–1^. Within 1–2 milliseconds, this electric field will provide the driving force for repassivation by interaction with water molecules. Electron accumulation in the metal will also shift the surface potential to more negative values. Hence, the OCP of a surface covered with an abraded oxide film will shift to a more cathodic (negative) value. Consequently, the corresponding reduction reactions will rise until the excess electrons are removed and the potential slowly shifts positively to its initial OCP value. These shifts in OCP change the structure of the oxide film and the reactions that are taking place, and can result in stray electric currents that might affect biological processes in the neighbouring tissue [40].

**Figure 9:**
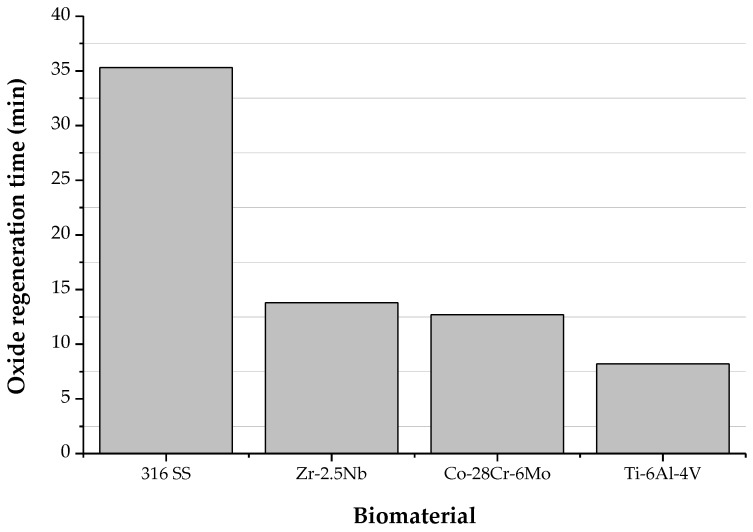
Regeneration time of surface oxide films on biomaterials. Drawn based on values from Hanawa [126].

The time required for repassivation, also known as regeneration time, is different for different biomaterials, see Figure 9 [126]. The corrosion rate after disruption and the amount of released metal ions are affected by this repassivation time. The repassivation time is longer for Ti in Hank’s solution than in saline but is unaffected by pH. Moreover, the outer layer of the surface oxide film regenerated on CP-Ti in Hank’s solution has been found to contain phosphate ions. The film thus consists of titanium phosphate-containing titanium oxide and titanium oxyhydroxide. Calcium and phosphate ions are also adsorbed onto the film after regeneration, and CaP or calcium-titanium-phosphate are formed on the outermost surface. In the case of Ti–6Al–4V too, CaP was observed on the surface oxide film regenerated in Hank’s solution. In contrast, only phosphate, without calcium, is formed on Ti–56Ni, Ti–Zr and Zr-based biomedical alloys. Thus, the composition of surface oxide layer and its interaction with the environment are strongly dependent on the chemical composition of the biomaterial used. The stability of the surface oxide layers on 316L stainless steel and NiTi SMA is inferior compared to Ti–6Al–4V and Co–Cr alloys. Therefore, surface modification (e.g., coating) may be applied to improve the corrosion resistance [39,126].

## 5. Common Metallic Biomaterials and Their Corrosion Performance

Metals and alloys have a large range of biomedical applications, including devices for fracture fixation, partial and TJR, external splints, craniofacial plates and screws, braces and traction apparatus, dental amalgams, cardiovascular surgery (e.g., parts of artificial hearts, balloon catheters and valve replacements) and so forth. The high modulus and yield stress coupled with the ductility of metals make them suitable for high load bearing without suffering from large deformations and permanent dimensional changes. The compositions most commonly used for load-bearing applications include stainless steels, cobalt-based wrought or cast alloys and titanium-based alloys. In addition, the NiTi SMA has attracted much attention due to its ability to reproduce its original shape upon exposure to body temperature and its pseudoelastic properties (*E*_eff_ ~ 40 GPa) which enable, among others, the manufacture of low-stiffness, high springback, orthodontic wires. Dental implants are frequently manufactured from CP-Ti or titanium alloys, amalgams and precious metals (e.g., Au). Noble metals such as Pt and Pt-Ir are also being used as electrodes in cardiac pacemakers and other neuromuscular stimulatory devices. Copper is used in contraceptive intrauterine devices (IUDs). Magnesium and some other biodegradable metals are being considered for bone screws and stents. Small metallic medical devices are used in a wide range of other implants, including skin and wound staples, vascular endoprostheses, filters and occluders. Although metals exhibit high strength and toughness, they have several drawbacks, mainly high modulus of elasticity, *E* (which might cause stress shielding of bone) and susceptibility to chemical and electrochemical degradation. This susceptibility is usually increased by the action of applied forces and wear. The combination of a relatively corrosive environment and a poor tolerance of the body to even small concentrations of most metallic corrosion products excludes from consideration most metallic materials. In this Section, the main metals and alloys currently used as biomaterials will be discussed. Obviously, given the page limitation and the large global R&D activities in this field, it is impossible to discuss them all. Yet, similar approaches for corrosion characterization and control may be applied to other metals and alloys too.

### 5.1. Stainless Steels

Various versions of the Pourbaix diagram of iron have been included in articles dealing with biomaterials corrosion. Most of them adapt the Pourbaix diagram for 25 °C, some mix between hydrous and anhydrous compounds of iron. Hence, it is worthy to remind in this review article how the Pourbaix diagram of iron should be constructed for hydrous conditions as in the body and a temperature of 37 °C. The reader can then use a similar procedure to construct the Pourbaix diagrams for other metals used in medical devices. In order to keep this part concise, only one example of each type of line in the diagram will be discussed. 

**Figure 10:**
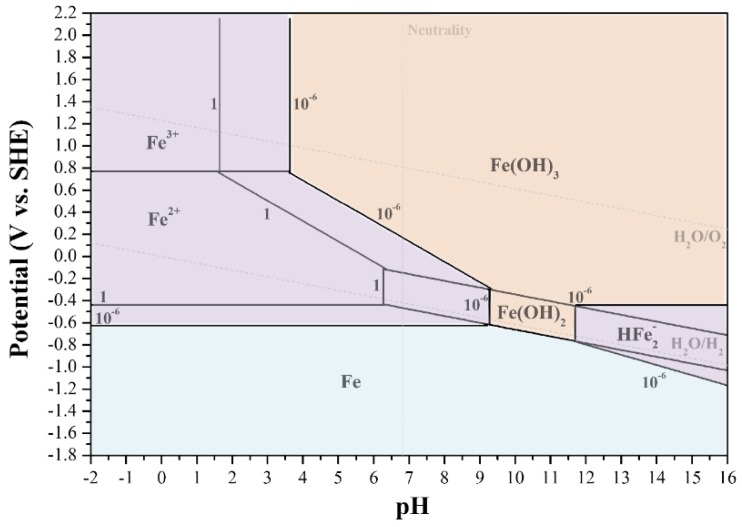
The potential-pH diagram for iron at 37 °C and wet (hydrous) corrosion products. The lines for two concentrations (10^–6^ M and 1 M) of soluble species are drawn.

The corrosion of iron and formation of rust (Fe_2_O_3_·H_2_O) takes place in several stages. The first and most fundamental step is the oxidation of iron to its lowest stable valence state. Figure 10 illustrates the Pourbaix diagram for iron at 37 °C (at which 2.3*RT*/*F* = 0.06144 V) in the presence of water or humid environments (i.e., the hydrous form). This diagram was calculated by considering all possible reactions associated with iron in wet or aqueous conditions (see some examples below), excluding dry forms of corrosion products such as the black ferrous oxide (FeO), black magnetite (Fe_3_O_4_) or red-brown haematite (Fe_2_O_3_). The equilibrium lines in this diagram are drawn for dissolved ion concentrations of either 1 μM or 1 M. Fe(OH)_2_ and Fe(OH)_3_ are considered as the only solid phases, in addition to Fe, with a negligible solubility product. However, these compounds, despite their thermodynamic stability under the considered conditions, do not have a protective nature because of their structure and the presence of defects in the crystal structure. Due to the effects of environmental variables, for example, the presence of hydrogen carbonates/bicarbonates and phosphates, chloride ions and changes in the concentration of corrosion products, the stability limits of each phase or chemical species in this Pourbaix diagram may change; furthermore, other ferrous compounds may form. 

In the construction of the Pourbaix diagram of iron the following standard chemical potentials are used [57]: $\mu_{H^{+}}^{0}=0$, $\mu_{O_{2}}^{0}=0$, $\mu_{H_{2}O}^{0}=-56,690\;\mathrm{cal}\;{\mathrm{mol}}^{-1}$, $\mu_{OH^{-}}^{0}=-37,570\;\mathrm{cal}\;{\mathrm{mol}}^{-1}$, $\mu_{\mathrm{Fe}}^{0}=0$, $\mu_{Fe^{2+}}^{0}=-20,300\;\mathrm{cal}\;{\mathrm{mol}}^{-1}$, $\mu_{Fe^{3+}}^{0}=-2,530\;\mathrm{cal}\;{\mathrm{mol}}^{-1}$, $\mu_{\mathrm{Fe}O_{4}^{2-}}^{0}=-111,685\;\mathrm{cal}\;{\mathrm{mol}}^{-1}$, $\mu_{\mathrm{Fe}{\left(\mathrm{OH}\right)}_{2}}^{0}=-115,570\;\mathrm{cal}\;{\mathrm{mol}}^{-1}$, $\mu_{\mathrm{Fe}{\left(\mathrm{OH}\right)}_{3}}^{0}=-166,000\;\mathrm{cal}\;{\mathrm{mol}}^{-1}$, $\mu_{\mathrm{HFe}O_{2}^{-}}^{0}=-90,627\;\mathrm{cal}\;{\mathrm{mol}}^{-1}$, $\mu_{\mathrm{Fe}O_{4}^{2-}}^{0}=-111,685\;\mathrm{cal}\;{\mathrm{mol}}^{-1}$. The precipitation constants, *K*_sp_, for Fe(OH)_2_ and Fe(OH)_3_ are taken as 2 × 10^–15^ and 1.1 × 10^–36^, respectively. 

For water, the vertical line of neutrality is drawn at pH = 6.81, while the lower and upper borders of the water stability domain are defined by

(15) $$E_{\mathrm{rev}}=-0.06144\;\mathrm{pH}\;\mathrm{for}\;H_{2}{O/H}_{2},$$

and

(16) $$E_{\mathrm{rev}}=+1.229-0.06144\;\mathrm{pH}\;\mathrm{for}\;H_{2}{O/O}_{2},$$

respectively.

For the electrochemical equilibrium

(17) $${\mathrm{Fe}}_{\left(\mathrm{aq}\right)}^{2+}+2e^{-}\;↔Fe_{\left(s\right)},$$

the Nernst equation yields:

(18) $$E_{\mathrm{rev}}=-\;0.440\;+\;\frac{0.06144}{2}\;\log\left[Fe^{2+}\right],$$

from which we get *E*_rev_ = –0.62432 and –0.44 V for [Fe^2+^] = 1 × 10^–6^ M or 1 M, respectively. These are horizontal lines.

The *chemical equilibrium* between Fe^2+^ and Fe(OH)_2_ is a precipitation reaction that can be written for acidic media as

(19) $${\mathrm{Fe}}_{\left(\mathrm{aq}\right)}^{2+}+2H_{2}O_{\left(l\right)}\;↔\mathrm{Fe}{\left(\mathrm{OH}\right)}_{2(s)}+2H_{\left(\mathrm{aq}\right)}^{+},$$

or for alkaline media as

(20) $${\mathrm{Fe}}_{\left(\mathrm{aq}\right)}^{2+}+2OH_{\left(\mathrm{aq}\right)}^{-}\;↔\mathrm{Fe}{\left(\mathrm{OH}\right)}_{2(s)}.$$

Note that white Fe(OH)_2_ is the hydrous form of FeO with one H_2_O molecule; accordingly will be the difference in the standard chemical potential. From the definition of the precipitation constant and a value of *K*_sp_ = 2 × 10^–15^ for Fe(OH)_2_ we can write

(21) $$2\times 10^{-15}=\left[Fe^{2+}\right]\times {\left[OH^{-}\right]}^{2}.$$

But, at 37 °C

(22) $$\log\left[OH^{-}\right]=\mathrm{pH}-13.62,$$

thus,

(23) $$\mathrm{pH}=6.271-\frac{1}{2}\log\left[Fe^{2+}\right].$$

Thus, we get pH = 9.271 or 6.271 for [Fe^2+^] = 1 × 10^–6^ M or 1 M, respectively. These are vertical lines on the Pourbaix diagram.

The electrochemical equilibrium between Fe^2+^ and Fe(OH)_3_ can be represented by

(24) $${\mathrm{Fe}(\mathrm{OH})}_{3(s)}{+3H}_{\left(\mathrm{aq}\right)}^{+}+e^{-}\;↔{\mathrm{Fe}}_{\left(\mathrm{aq}\right)}^{2+}+3H_{2}O_{\left(l\right)}.$$

Note that 2Fe(OH)_3_ is the hydrous form of Fe_2_O_3_ with three water molecules. Using the Nernst equation we can write:

(25) $$E_{\mathrm{rev}}=E_{\mathrm{Fe}{\left(\mathrm{OH}\right)}_{3}|Fe^{2+}}^{0}-\;0.06144\;\log\frac{\left[Fe^{2+}\right]}{{\left[H^{+}\right]}^{3}}.$$

Using the standard chemical potentials we can *calculate the standard reduction potential* of reaction (24):

(26) $$E_{\mathrm{Fe}{\left(\mathrm{OH}\right)}_{3}|Fe^{2+}}^{0}=-\frac{\left(-20,300+3\times \left(-56,690\right)\right)-\left(-166,000+3\times 0\right)}{23,060\times 1}=+1.057\;V.$$

Substituting into Equation (25), we get

(27) $$E_{\mathrm{rev}}=1.057-0.18432\;\mathrm{pH}-0.06144\;\log\left[Fe^{2+}\right].$$

Ferrate ($\mathrm{Fe}O_{4}^{2-}$), a hexavalent iron species, is a very strong oxidizing agent. It is unstable in acidic (where the half-cell potential is very high) and weakly alkaline solutions. Ferrate undergoes rapid decomposition under near-neutral and basic conditions. It can practically be observed for short periods of time only at pH > 9.2 [128] or even pH > 10.0 [129,130] and is irrelevant to the body environment. Nevertheless, it should be noted that its domain was originally drawn by Pourbaix with a question mark, also for acidic media [57]. Since then it has become clear that this is not the case.

When drawing a Pourbaix diagram, it is recommended that the equilibrium lines be taken one at a time, progressing from the immune, metallic state through the various oxidized states, to establish the ends of each of the lines. It is usually difficult to draw all the lines and to exclude those lines and portions of lines that are redundant or inadequate. Alternatively, one may use any of dozens dedicated software for drawing Pourbaix diagrams, such as PhreePlot, The Geochemist’s Workbench or Chesta. 

From Figure 10 it is evident that at a concentration of 1 μM, potentials more positive than −0.62 V versus SHE and pH values below ca. 9.3, ferrous ion (Fe^2+^) is the stable substance. This indicates that iron will corrode under these conditions. In other regions of the iron Pourbaix diagram, it is evident that the corrosion of iron produces ferric ions (Fe^3+^), ferric hydroxide (Fe(OH)_3_) and ferrous hydroxide (Fe(OH)_2_). At pH ≥ 12.3, the anion ${\mathrm{HFeO}}_{2}^{-}$ is thermodynamically stable, showing that iron can also be amphoteric to some extent. There are no soluble species between pH 9.3 and 12.3 and iron could pass directly from the immune to the passive region as the potential is increased. Also, it should be possible to passivate iron in solutions where the pH exceeds about 4, by oxidizing the surface, either chemically or electrochemically. In this case, the potential can be forced to cross the active region (where Fe^2+^ is the stable species) rapidly, to reach the region of passivity, where Fe(OH)_3_ is stable. The presence of a relatively large immunity region in Figure 10 indicates that iron may corrode much less under these potential-pH conditions. This diagram also indicates that if the potential of iron is made sufficiently negative or shifted cathodically below approximately −0.62 V versus SHE in acidic, neutral or slightly alkaline environments, iron will be cathodically protected [46].

Now that the Pourbaix diagram of iron has been discussed, we shall move on and discuss the use of stainless steels as biomaterials. As mentioned in the Introduction, the first stainless steel developed specifically for implantation was the “vanadium steel,” in the early 1900s [4]. The early successful devices were fracture fixation plates. However, the surgeons quickly learnt that these devices failed due to mechanical, corrosion and poor biocompatibility reasons. Iron and steel were found to dissolve rapidly in vivo and caused erosion of the adjacent bone. Thus, a 18Cr–8Ni stainless steel was introduced to the market in 1926 and was reported to exhibit improved strength and corrosion resistance. Later that year, Mo was added to this steel in order to improve its localized corrosion resistance; this alloy became known as 316 stainless steel. In 1943, type 302 stainless steel was recommended to the U.S. Army and Navy for bone fixation [73]. During the 1950s, the carbon content was reduced to 0.03 wt%; this alloy is known as 316L stainless steel (UNS S31673). Because type 316L stainless steel is more corrosion resistant both in vitro and in vivo than type 316, ASTM recommends the former for implant fabrication. Standards of stainless steels include ASTMs F138 (bar and wire) [131], F139 (sheet and strip) [132], F899 [133], F1586 (nitrogen alloyed) [134] and ISO 5832-1 [135]. Chemical compositions of certain stainless steels currently or potentially used as biomaterials are provided in Table 5.

Austenitic stainless steels have an fcc (γ) structure, unless they undergo a phase transformation due to severe plastic deformation. Chromium soluted evenly within the microstructure allows the formation of a thin (typically 10–50 Å thick), amorphous chromium oxide (Cr_2_O_3_) layer on top of the steel. The ionic bonding in this layer protects the surface from electrochemical degradation. Hence, stainless steels are often pickled in nitric acid to enhance the growth and thickening of this passive layer. Austenitic stainless steels are used either in the annealed state or in the cold work state. The latter improves the tensile strength and fatigue properties, with some trade-off in corrosion resistance. Advantages of stainless steels include high strength, availability, cost effectiveness and good formability (namely, good cold work, machinability and weldability). Shortcomings, on the other hand, include possible release of toxic ions (namely, Cr and Ni) due to the combined action of corrosion and wear, and stress shielding of adjacent bone due to high modulus of elasticity (*E* ~ 190 GPa), which is about ten times that of bone. Consequently, stainless steels are used nowadays mainly as temporary medical devices or in elder patients.

Hanawa [126] discussed briefly the chemical composition and reconstruction of the oxide films on austenitic stainless steels. The composition was found to comprise of Fe and Cr, small amounts of Mo but no Ni. After mechanical polishing in water, the surface of 316L consisted of iron and chromium oxides containing small amounts of Ni, Mo and Mn oxides. The surface oxide also contained a large amount of (OH)^–^. Following immersion in Hank’s solution and incubation with cells, CaP was formed on and within the film. Sulphate was also adsorbed on the surface of the oxide film and was reduced to sulphite and/or sulphate in cell culture medium.

Test procedures and evaluation criteria for the corrosion resistance of surgical instruments made of stainless steels and intended for reuse in surgery are described in ASTM F1089 [136]. Venugopalan and Gaydon [137] studied the corrosion behaviour of 316L stainless steel in deaerated neutral Hank’s balanced salt solution (HBSS) at 37 °C by means of cyclic potentiodynamic polarization experiments. In comparison to titanium alloys, the corrosion potential of 316L (and many other stainless steels) is typically more active and its corrosion rates are higher. A true potential-independent passive region is typically not apparent in the polarization curve of stainless steels. In addition, the passive region was small and a fast transition to a transpassive region was evident. The steel did not show *E*_p_ and the large hysteresis loop indicated susceptibility to localized corrosion. The following values were recorded: *E*_corr_ = –130 mV, *j*_corr_ = 186 nA·cm^–2^, CPR = 1.94 μm·y^–1^.

Stainless steels are susceptible to pitting corrosion in saline. Depending on the type of alloy used, the pitting potential of stainless steels may be set in a potential region relevant to biomedical applications [38].

The corrosion performance of stainless steels has been investigated both in vitro and in vivo in numerous papers. For example, von Fraunhofer et al. [138] studied the effect of antibiotics additions to saline on the corrosion potential of the major surgical alloys, including stainless steels. It was found that only one antibiotic, oxytetracycline, exerted a significant effect on the electrochemical behaviour, generating an anodic shift of 120–250 mV in *E*_corr_. Shih et al. [139] showed that while the amorphous oxides on either 316L stainless steel or Nitinol were resistant against localized corrosion both in vitro and in vivo, their counterpart polycrystalline oxides experienced severe pitting or crevice corrosion. Sutow et al. [140] showed that the in vitro crevice corrosion of cold worked 316LVM steel could be reduced following passivation in 30% HNO_3_. Bundy et al. [141] conducted in vitro tests and showed that cyclic anodic polarization tests with highly loaded fracture mechanics samples resulted in a lower *E*_b_ and disruption of the passive films. The current density increased by more than an order of magnitude in the presence of plastic deformation, compared to loading to below yield stress. Brown and Merritt [142] studied the fretting corrosion of stainless steel plates in vitro. The results showed a tenfold decrease in fretting corrosion when 10% solution of foetal calf serum was added to saline. In a later publication [143], proteins were found to increase the corrosion rate of 316L in the static mode, while decreasing the corrosion rate in the fretting mode. The presence of proteins apparently caused an increase in the anodic Tafel constant and a decrease in the cathodic Tafel constant of this material.

During the last decades, the role of sulfide inclusions in initiating pitting corrosion in stainless steels was recognized [144], leading to the development of type 316LVM stainless steel. This steel is vacuum melted (VM) to reduce the non-metallic inclusions content [145]. Because biomaterials are more prone to localized corrosion in dental applications, a further improvement has been made, forming ultraclean high nitrogen austenitic stainless steels [146]. As mentioned in Section 2, a PREN of 26 and greater has been recommended to prevent in vivo pitting corrosion of stainless steels, in comparison to the value of 40 usually required for stagnant seawater [54]. Additions of nitrogen to 316L stainless steel increase the PREN of this alloy to above 26 and also increase the ultimate tensile strength (UTS), although the trade-off is some loss of ductility (as observed by elongation to fracture). A typical composition (UNS S31675) is given in Table 5.

**Figure 11:**
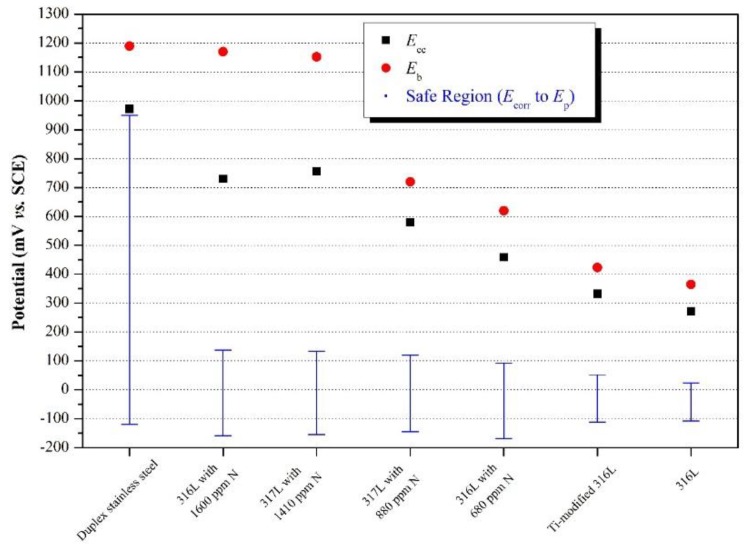
Comparison between selected corrosion resistance characteristics of several stainless steels in Hank’s solution, illustrating the effect of nitrogen concentration in the steel [37]. Reproduced with permission from De Gruyter.

Mudali et al. [37] identified different failure mechanisms in stainless steel orthopedic devices and suggested routes to improve the corrosion behaviour of these steels. Alloy modification was carried out through additions of titanium and nitrogen as alloying elements. A super-ferritic stainless steel (Sea-Cure^®^, UNS S44660, ASTM A268 [147], see Table 5), that was commercially introduced in 1979 to provide high strength and high resistance against localized corrosion and SCC in seawater, was also characterized for comparison. A second comparison was made to SAF 2205 (UNS S31803) duplex stainless steel [148], see Table 5. This steel acquires a ferrite/austenite two-phase microstructure and is characterized by good weldability and an attractive combination of high strength, high toughness and excellent corrosion resistance (PREN = 35; high resistance to SCC in chloride-bearing environments, as well as to erosion corrosion and to corrosion fatigue). Another stainless steel tested was the 317L (UNS S31703) [149], see Table 5, which is expected to have a significantly higher resistance to pitting and crevice corrosion than 316L at ambient temperatures. Nitrogen was added in contents of 680–1600 ppm. Figure 11 summarizes several corrosion parameters that were obtained from cyclic potentiodynamic polarization curves in Hank’s solution. It is apparent that the increase of nitrogen concentration in austenitic stainless steels results in higher values of *E*_b_, indicating a higher resistance to pitting corrosion. The *E*_b_ value for the duplex stainless steel is nobler than *E*_b_ of the currently used type 316L stainless steel. The super-ferritic stainless steel showed immunity to pitting and crevice corrosion attack and is thus excluded from Figure 11. The mechanisms by which each of the above steels attains its pitting corrosion resistance are summarized elsewhere [37]. Another parameter shown in Figure 11 is the critical crevice potential, *E*_cc_. Mudali and Dayal [150] defined this parameter in anodic polarization curves as the potential above which significant crevice attack occurred on the surface of the specimen. Hence, the higher *E*_cc_, the better would be the resistance of the steel against crevice corrosion. In practice, *E*_cc_ was identified as the potential at which a monotonic increase in the anodic current exceeding 25 µA was noticed. Figure 11 shows that 316L with high nitrogen contents and duplex stainless steel exhibit higher values of *E*_cc_. Finally, the potential range (from *E*_corr_ to *E*_p_) which is safe against localized corrosion attack is also marked in Figure 11. Based on the aforementioned results, the resistance of the different stainless steels to pitting corrosion was ranked [37] as follows: super-ferritic > duplex > 316L with 1600 ppm nitrogen > 317L with 1410 ppm nitrogen > 317L with 880 ppm nitrogen > 316L with 680 ppm nitrogen > Ti-modified 316L > reference 316L. With respect to crevice corrosion resistance, however, the order slightly changed, as follows: super-ferritic > duplex > 317L with 1410 ppm nitrogen > 316L with 1600 ppm nitrogen > 317L with 880 ppm nitrogen > 316L with 680 ppm nitrogen > Ti-modified 316L > reference 316L.

Advanced stainless steels for surgical implants include the low-nickel (or nickel-free) austenitic stainless steels [151]. One example is UNS S29108 [152], known also as BioDur^®^ 108 from Carpenter Technology Corporation (see Table 5). This alloy was designed to minimize problems associated with Ni toxicity and is produced by the electro-slag re-melting (ESR) process to guarantee its microstructural integrity and cleanness. It has better mechanical properties (both static and fatigue) and localized corrosion resistance compared to type 316L alloy.

### 5.2. Cobalt-Chromium Alloys

The industrial use of pure cobalt dates back to the beginning of the 20th century. However, in the form of a pure metal, it is not sufficiently ductile and corrosion resistant. Between 1907 and 1913 Haynes developed a series of cobalt-chromium and cobalt-chromium-tungsten alloys with good corrosion resistance. During the early 1930s, an alloy called Vitallium, with a composition Co–30Cr–7W–0.5C, was used for the preparation of metallic dental castings. Since these alloys were marketed in dentistry as an alternative to the expensive gold alloys, the dentists quickly adapted them widely, in particular for the large partial denture castings that require considerable amount of metal. More recently, cast Vitallium started to find use also in artificial joints. Wrought Vitallium is used in manufacturing of the stems of heavily loaded joints, such as femoral hip stems. Porous coated Co–Cr implants have been extensively used for bone ingrowth applications. Sintered beads, plasma flame sprayed metal powders and diffusion bonded fibre metal pads are different types of coatings applied on Co–Cr orthopedic implants [74,75].

Cobalt-based alloys are highly resistant to corrosion, including chloride-induced crevice corrosion. Galvanic corrosion is also of less concern compared to stainless steels. Cobalt-based alloys are fairly resistant to fatigue and to environment-induced cracking (EIC). They have reasonable toughness and elongation of more than 8% to fracture. However, wear processes can lead to release of toxic Cr, Co and Ni ions into the body. Another drawback of these alloys is their high modulus of elasticity, which enhances stress shielding compared to other alloys, including stainless steels and titanium-based alloys.

Hanawa [126] described the composition of the surface oxides on CoCrMo alloys. Typically, these are oxides of Co and Cr, without Mo. However, after mechanical polishing in DI water, there were also oxidic species of Mo and the overall film thickness was about 2.5 nm. The surface film contained also high (OH)^–^ content, that is, it was hydrated or oxyhydroxidized. After dissolution in Hank’s solution, the surface oxide consisted of chromium oxide (Cr^3+^), which contained also molybdenum oxides (molybdenum having +4, +5 and +6 valences). Chromium and molybdenum were found to be more widely distributed in the inner layer compared to the outer layer of the oxide film. It was also argued that in body fluids Co is entirely dissolved and the surface oxide converts to chromium oxide containing a small amount of molybdenum oxide. CaP is also formed on the top surface.

There are two major types of cobalt-chromium biomedical alloys–cast CoCrMo and wrought CoNiCrMo alloys. While the former has been used for years in dentistry and more recently in orthopedics (for TJR), the latter is used for manufacturing hip stems and knee joints. The abrasive wear properties of the wrought alloy are similar to those of the cast alloy. However, the superior fatigue and UTS of the wrought alloy make it suitable for applications that require long service life without fatigue or fracture. The CoCrMo alloy is particularly susceptible to work hardening; hence, the normal manufacturing procedures selected for other metals are not applicable. Standards of cobalt-chromium alloys include ASTMs F75 (cast UNS R30075 alloy) [153], F90 (wrought UNS R30605 alloy, Alloy L-605) [154], F562 (wrought UNS R30035 alloy) [155], F799 [156] and F1537 [157] (forged UNS R31537, R31538 and R31539 alloys) and F1058 (wrought UNS R30003 and R30008 wire and strip, known as Elgiloy) [158]. ISO standards include 5832-4 (cast CoCrMo alloy) [159], 5832-5 (wrought CoCrWNi alloy) [160], 5832-6 (wrought CoNiCrMo alloy) [161], 5832-7 (forgeable and cold-formed CoCrNiMoFe alloy) [162], 5832-8 (wrought CoNiCrMoWFe alloy) [163] and 5832-12 (wrought CoCrMo alloy) [164]. Co-35Ni-20Cr-10Mo is commonly referred to as MP35N or in its low-Ti form—35N LT [145]. Selected compositions are given in Table 5.

Venugopalan and Gaydon [137] studied the corrosion behaviour of cast and wrought Co-based alloys in deaerated neutral HBSS at 37 °C by means of cyclic potentiodynamic polarization tests. The following values were recorded for the cast alloy: *E*_corr_ = –104 mV, *j*_corr_ = 760 nA·cm^–2^, CPR = 6.77 μm·y^–1^. The following values were recorded for the wrought alloy: *E*_corr_ = –171 mV, *j*_corr_ = 94 nA·cm^–2^, CPR = 0.839 μm·y^–1^. These values should be compared to those reported in Section 5.1 above for 316L stainless steel under the same test conditions. The cast alloy had inferior corrosion behaviour compared to the wrought alloy. Both alloys did not exhibit an active-passive transition. Their passive regions were not as potential independent as those of titanium and its alloys. They also showed a secondary peak at about 500–700 mV, corresponding to the oxidation reactions of Cr at a higher valence level (this was more distinct in the case of the wrought alloy). There was a true passive-to-transpassive transition, with a visibly defined *E*_b_ [33]. The current increase in this case was due to transpassive dissolution by oxidation of the Cr_2_O_3_-rich passive film into soluble $\mathrm{Cr}O_{4}^{2-}$ species and not due to pitting corrosion [38]. For the cast alloy, the reverse scan did not cross over the forward scan immediately but essentially traced a loop in doing so. The wrought alloy did not show hysteresis as that of the cast alloy, indicating that it can repair damage to its oxide layer faster (or better). The finer grain size and more uniform distribution of carbides in the wrought alloy contribute to its chemical homogeneity and improved corrosion behaviour [33]. 

### 5.3. Titanium and its Alloys

Titanium and its alloys are the most widely used materials for medical implants [165]. Titanium is light (*ρ* = 4.51 g·cm^–3^), biologically and chemically inert, biocompatible and has low electrical and thermal conductivity (*σ* = 2.3 × 10^4^ Ω^–1^·cm^–1^ at 22 °C, *κ* = 22 W·m^–1^ K^–1^ at 27 °C, respectively) in comparison to other metals. Since it is weakly paramagnetic, image interference in magnetic resonance imaging (MRI) or computed tomography (CT) scans is not observed. In addition, because it has a coefficient of thermal expansion which is similar to that of the bone, MRI testing complications due to thermal expansion and distortion are minimized. The modulus of elasticity of titanium and its alloys is closer to that of bone than the moduli of stainless steels and cobalt-based alloys, thus the concern over stress shielding is reduced. The oxide layer on the surface of titanium and its alloys is formed due to the high affinity of titanium to oxygen, provides corrosion resistance and allows for histological osseointegration. This oxide layer, typically several nanometres thick, is thermodynamically stable and repairs itself rapidly as long as there is low concentration (several ppm) of oxygen or water in the environment. However, because osseointegration of titanium implants takes a long time (typically three to six months), they are often coated with hydroxyapatite (HAp) and other calcium phosphates [7,8,9,10,11,12,13,14,15,16,17,18,19,20,21,23,27,28,29,30,31].

Relevant ASTM standards for titanium and its alloys include F67 for unalloyed titanium (UNS R50250, R50400, R50550 and R50700) [166], F136 for wrought Ti–6Al–4V ELI (UNS R56401) [167], F1472 for wrought Ti–6Al–4V (UNS R56400) [168], F1713 for wrought Ti–13Nb–13Zr (UNS R58130) [169], F1295 for wrought Ti–6Al–7Nb (UNS R56700) [170], F1813 for wrought Ti–12Mo–6Zr–2Fe (UNS R58120) [171] and F1580 for titanium and Ti–6Al–4V powders for coatings [172]. ISO standards include 5832-2 for unalloyed titanium [173], 5832-3 for wrought Ti–6Al–4V alloy [174], 5832-10 for wrought Ti–5Al–2.5Fe alloy [175] and 5832-11 for wrought Ti–6Al–7Nb alloy [176]. Typical chemical compositions of titanium and its alloys used as biomaterials are provided in Table 6. Typical mechanical properties of selected Ti-based alloys are summarized in Table 7, in comparison to some other common biomaterials.

Titanium and its alloys are used in a range of applications, including fracture fixation devices (plates and screws), spinal fixation devices, THR femoral components, ligament anchorage screws, dental implants and maxillofacial surgery, heart pacemaker housings and artificial heart valves and components in high-speed blood centrifuges. Generally, alloys are used for joint replacement components because of their better mechanical properties compared to CP-Ti. 

The Ti–6Al–4V alloy has a dual microstructure, which consists of a fine-grained hcp (α) phase and a distributed bcc (β) phase. The presence of elements such as Al, O, N, Ga and C stabilizes the α phase, whereas metals such as V, Nb, Ta and Mo stabilize the β phase. The microstructure and mechanical properties of Ti–6Al–4V are greatly dependent on the thermomechanical processing treatments. If the material is cooled too slowly, the β phase becomes more noticeable and lowers the strength and corrosion resistance of the alloy. Ti–6Al–4V is still the most common alloy in medical use. It combines good workability, heat treatment ability and weldability, as well as high corrosion resistance, high strength and good biocompatibility. Interstitial atoms such as oxygen, nitrogen and carbon increase the strength significantly, nitrogen having an almost double effect per atom. The extra-low interstitials (ELI) grade, containing small amounts of oxygen, carbon, nitrogen and hydrogen, is the one commonly used for biomedical applications (e.g., for stems of artificial hip joints). The Ti–6Al–4V ELI alloy has excellent toughness, because the impurities decrease the fatigue strength with a notch effect. Its yield strength in the forged and annealed condition is very high (896 MPa), much higher than that of 316L stainless steel (331 MPa) or wrought Co-Cr alloys in the annealed condition (448–648 MPa), making plastic deformation of the titanium alloy limited even under a large load.

Drawbacks of titanium and its alloys include high price, high susceptibility to friction and wear (as reflected by a high coefficient of friction) and low shear strength. Machining of titanium is not cost-effective. Although precision casting reduces the manufacturing costs, cast titanium alloys exhibit low fatigue strength and low elongation compared to wrought alloys due to the coarse microstructure of the cast alloys. Therefore, supplement microstructural refinement is often required to improve the mechanical properties while preserving the shape of the product. Thermomechanical processes with post heat treatment have been found to increase the strength, elongation and fatigue strength of α + β titanium alloys. Another cost-effective process is powder metallurgy (PM). This process is very useful in producing more homogenous β alloys that contain alloying elements with high melting point, such as Nb and Ta [177].

The presence of Zr and Nb in titanium has been found to reduce significantly the modulus of elasticity as well as to improve the corrosion resistance in vivo. In 1986, the dual phase Ti–6Al–7Nb was introduced into clinical use (this alloy was first synthesized in 1977) as a possible alternative for Ti–6Al–4V, realizing that Nb is both cheaper and more biocompatible than V. In this regard, VO_2_ generated by passivation of the metal surface is thermodynamically unstable; some V could be released into the human body and cause toxic effects. Ti–6Al–7Nb has a α + β grain structure. It has been shown to possess higher UTS, yield strength and strain to fracture compared to Ti–6Al–4V, as well as better workability [178,179,180,181,182]. For example, the hot-forged alloy has *E* = 110 GPa, *σ*_y_ = 900–1000 MPa, *σ*_uts_ = 1000–1100 MPa, *ε* = 10–15%, reduction of area (RA) of 15–45%, and rotating bending fatigue strength of 500–600 MPa. Ti–6Al–7Nb has been produced by casting, hot rolling, hot forging and PM methods. In recent years, however, this alloy has been processed also by different three-dimensional (3D) printing techniques, mainly selective laser melting (SLM) [183] and electron beam melting (EBM). Current applications of this alloy include artificial hip and knee joints, bone plates, screws for fracture fixation, dental applications, cardiac valve prostheses, pacemakers and artificial hearts.

Another alloy, Ti–13Nb–13Zr [169], exhibits a near-β structure, higher adhesion of osteoblasts and lower bacterial adhesion than CP-Ti and Ti–6Al–4V. This alloy is useful in designing cardiovascular implants because the ZrO_2_ passive film is thrombogenically compatible with blood. The corrosion resistance of this alloy is also improved due to the presence of ZrO_2_ and Nb_2_O_5_, which strengthen the TiO_2_ film formed on the surface. Hence, many contemporary titanium-based alloys are β-stabilized in order to reduce the elastic modulus towards that of bone, although the trade-off is typically lower wear resistance and fatigue strength. The excellent corrosion behaviour of Ti–13Nb–13Zr in different types of SBFs has been validated. The chemical composition of this alloy, with all alloying elements exhibiting a large range of stable passivity, allows it to remain remarkably passive in many environments. With respect to wear-accelerated corrosion (*tribocorrosion*), this alloy was found to have lower overall degradation than Ti–6Al–4V and even than Ti–6Al–7Nb (that was somewhat better than Ti–6Al–4V) [41].

**Figure 12:**
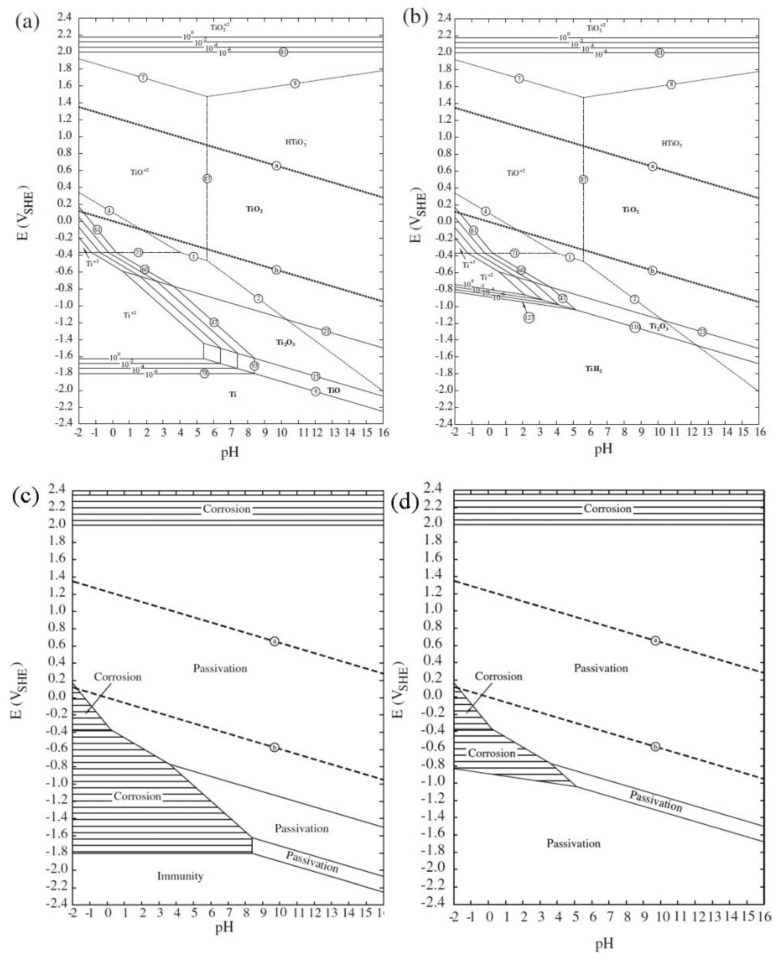
Pourbaix diagram of the Ti–H_2_O system at 25 °C: (**a**) not taking into account titanium hydrides and (**b**) taking into account titanium hydrides. Line numbers correspond to reaction numbers in Ref. [186]. Solid lines bound the stability regions of the solid phases in equilibrium with 10^–6^, 10^–4^, 10^–2^ and 100 M activity values of the soluble titanium species. Fine broken lines mark equilibria between the dissolved species. (**c**) and (**d**) are the corresponding Pourbaix diagrams for (a) and (b), respectively, when marking only the immunity, passivation and corrosion domains [186]. Reprinted with permission of Elsevier.

The Pourbaix diagram for the Ti–water system at 25 °C [57,58] is shown in Figure 12. Pure titanium is a base metal with a very negative standard reduction potential $\left(E_{\mathrm{Ti}/Ti^{2+}}^{0}=-1.63\;V\;\mathrm{vs}.\mathrm{SHE}\right)$. Although titanium is thermodynamically reactive, it is very corrosion resistant thanks to the instantaneous generation of a hard, tightly adherent, passive film that is stable at all pHs at oxidizing potentials [38,40,41,184]. Thermodynamically, TiO_2_ is stable within a large pH range, as evident from Figure 12. However, complexing agents, such as HF and H_2_O_2_, can cause substantial dissolution. Fluorides have significant effect on Ti corrosion, in particular in dental applications, given the use of prophylactic fluoride-containing products in dental treatments [41]. Titanium corrodes only at low pH in solutions without oxidizers. As the passivation potential of Ti is more negative than the reversible potential of hydrogen evolution and the critical current density for passivation is fairly low, spontaneous passivation of a bare Ti surface, even in deaerated acidic solutions, can be expected. However, the passivation reaction can be troubled by the slow kinetics (i.e., high overpotential) of the reduction HER on the Ti surface. This explains the observation of active dissolution of Ti under reducing conditions in acidic solutions (depending on the acid type and concentration). Yet, in oxidizing acids and in most neutral solutions, Ti passivates spontaneously [41].

CP-Ti and single-phase titanium-based alloys exhibit enhanced corrosion properties compared to most other metallic biomaterials. Venugopalan and Gaydon [137] studied the corrosion behaviour of CP-Ti and titanium alloy in deaerated neutral HBSS at 37 °C by means of cyclic potentiodynamic polarization experiments. The following values were recorded for the titanium alloy: *E*_corr_ = 95 mV, *j*_corr_ = 26 nA·cm^–2^, CPR = 0.232 μm·y^–1^. These values should be compared to those reported in sections 5.1.2 and 5.2 for stainless steel and Co-based alloys, respectively. The *E*_corr_ of the titanium alloy is nobler than that of 316L stainless steel and Co-based alloys, and the material enters a stable passive behaviour directly from the active regime without exhibiting an active-to-passive transition. Moreover, neither *E*_b_ nor *E*_p_ is evident, indicating that this material has a very protective oxide layer (see explanation in Section 4.1). Consequently, only minimal release of ions or by-product residues into the periprosthetic tissue occurs and this material may be classified as bioinert in the whole range of clinically relevant potential-pH combinations. Since CP-Ti, Ti–6Al–4V, Ti–6Al–7Nb and other Ti-based alloys used for biomedical applications do not show breakdown of passivity at potentials lower than 1 V, pitting corrosion by chloride ions only takes place at high anodic potentials not relevant to biomedical applications. Ti and its alloys are exception to other materials susceptible to pitting corrosion, in that chloride ions are not the most aggressive halides in initiating pitting corrosion. Hence, stable pitting corrosion of Ti and its alloys is not expected in vivo [38].

**Figure 13:**
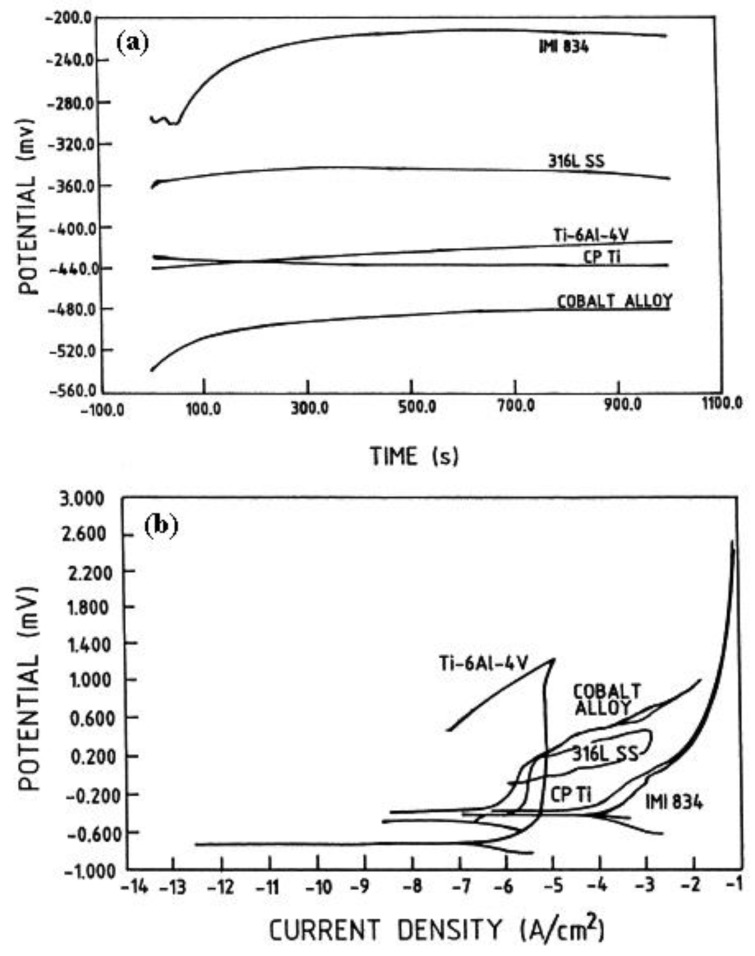
(**a**) OCP versus time and (**b**) cyclic potentiodynamic polarization curves of 316L stainless steel, Co-Ni-Cr-Mo alloy, CP-Ti, Ti–6Al–4V alloy and IMI 834 T-based alloy. Tests were conducted in deaerated Hank’s solution at 37 °C [185]. Reprinted with permission of Elsevier.

An interim summary of the corrosion characteristics of stainless steels, Co-based alloys and Ti-based alloys can be made with the aid of a study of Gurappa [185]. 316L stainless steel, Co-Ni-Cr-Mo alloy, CP-Ti, Ti–6Al–4V and a new Ti-based alloy (known as IMI 834), which contains Al, Sn, Zr, Nb, Mo and Si, were tested in deaerated Hank’s solution at 37 °C. Figure 13a shows the OCP of these materials, while Figure 13b shows their cyclic polarization curves. The OCP is the potential of the working electrode relative to the reference electrode when no potential or current is being applied to the cell. In case of a reversible electrode system, the OCP is also referred to as the equilibrium potential. Otherwise, it is called the rest potential, *E*_r_ or the corrosion potential, depending on the studied system. Metals with nobler (more positive) OCP are more thermodynamically stable than those with more negative OCP; the former will be less susceptible to uniform corrosion. The first step in most electrochemical tests is to measure the OCP. It is vital to allow sufficient time for the OCP to reach steady state (which indicates that the various corrosion reactions have assumed a constant rate) before beginning the next experiment. This could take between minutes and days [46]. From Figure 13a it is thus evident that for all materials, the formation of a passive film is evident from the shift in the positive direction of the OCP over time. Hysteresis loop in the potentiodynamic curve is evident only in the case of 316L stainless steel, indicating its high susceptibility to pitting and crevice corrosion. 

When exposed to different synthetic physiological solutions, titanium naturally forms a few nanometres thick passive film, which contains a relatively high concentration of oxygen vacancies. This titanium oxide layer may be described as an *n*-type semiconductor containing anion vacancies. The kinetics of titanium corrosion in neutral solutions is controlled by migration of oxygen vacancies across this oxide film [187]. Therefore, a thick TiO_2_ film containing a low concentration of oxygen vacancies will favour a very slow mass transport rate across the film. When the oxide is sufficiently thin (0.4–3 nm), electron exchange occurs between the redox electrolyte and the underlying metal by direct tunnelling or resonance tunnelling via intermediate states. A freshly abraded titanium surface immediately passivates, forming a low-crystalline rutile and/or anatase oxide layer. The oxygen concentration in the titanium oxide gradually decreases, from TiO_2_ at the outer surface, to Ti_2_O_3_ and TiO closer to the metal/oxide interface. Depending on the environment, this oxide may be covered with an amorphous or hydrated surface oxide, thus exhibiting a bilayer oxide structure. The thickness of the oxide layer can be increased by anodizing or thermal oxidation. The former process can yield an oxide layer several thousand angstroms thick, depending on the applied potential [188].

The characteristics of the surface oxide layer on titanium have been studied by different researchers. Hanawa [126] reviewed the composition, reconstruction and regeneration in biological environments of various surface oxides, including those on titanium and its alloys. The film on Ti–6Al–4V was found to be virtually the same as that on CP-Ti, although containing a small amount of Al_2_O_3_. Vanadium was not detected in the oxide layer. Calcium, phosphorus and sulphur were observed on the surface of CP-Ti surgically implanted into the human jaw. CaPs also form on titanium and its alloys by immersion in Hank’s solution and other SBFs. The repassivation rate was not affected by the pH of solution, dissolved oxygen or proteins [126].

Gilbert et al. [189] reported the effect of potential, pH and aeration on the titanium oxide film fracture and repassivation. In a subsequent work, Bearinger et al. [190,191] studied the morphological changes of TiO_2_ oxide films upon exposure to PBS and hydrogen peroxide-modified PBS solutions. Hydrogen peroxide (H_2_O_2_) is an oxidizing molecule, which is secreted by cells and is associated with wound healing and is thus present in significant quantities in fresh implant environments. The substrates were either CP-Ti or Ti–6Al–4V. The surfaces were subjected to simultaneous polarization or impedance testing and in situ electrochemical atomic force microscopy (EC-AFM) imaging to assess how the structure and properties of the passive oxide film are affected by changing potential and hydration. All samples were found covered with protective titanium oxide domes that developed in area and coalesced because of hydration and as a function of increasing applied potential and time. Reversal of dome growth did not happen when the potential was reduced, whereas the impedance behaviour was quasi-reversible, indicating that the structural and electrical properties were independent. Oxide growth occurred in part by lateral spreading and overgrowth of domes at the oxide/solution interface [190,191].

The temperature of the environment plays a significant role in the incidence of crevice corrosion of Ti and its alloys. The critical temperature for crevice corrosion is typically observed at around 70 °C. Nevertheless, already at 37 °C, the degradation behaviour is changed compared to room temperature. For example, the passive dissolution rate in neutral physiological saline solutions was found to slightly increase in the case of Ti–6Al–4V alloy (in contrast to CP-Ti) at body temperature compared to room temperature. In more aggressive acidic solutions, the temperature effect is even more distinct because, at higher temperature, less acidic solutions can result in surface activation; in this regard, the effect is more substantial for the Ti–6Al–4V alloy than for CP-Ti. Temperature increase also has a very pronounced effect on the metastable pitting activity of CP-Ti and Ti–6Al–4V. It should be noted that, at 37 °C, the frequency of pit nucleation does not decline to zero over time; hence, during long-term exposure, even a few activation/repassivation events could cumulatively contribute significantly to metal ion release. Apart from influencing the passive dissolution behaviour, rising the temperature to 37 °C influences the adsorption of Ca^2+^ and ${\mathrm{PO}}_{4}^{3-}$ ions on the surface of passive Ti or Ti alloys. It is thus evident that in order to assess the chemical, electrochemical and degradation behaviour of Ti alloys properly, experiments should be conducted at 37 °C [41].

Wear and corrosion processes result in release of vanadium and aluminium from Ti–6Al–4V into the body, thus causing hypersensitivity and other clinical complications [192]. The potentially toxic aluminium has been removed in the new Ti–50Ta alloy (an α + β alloy), which has shown excellent corrosion resistance and high tensile strength in vitro [193]. Ti–Ta alloys with 0–70 wt% Ta have shown increased corrosion resistance as the content of Ta was increased. The wear resistance of the Ti–Ta alloys was better than that of Ti–6Al–4V and the former were also non-cytotoxic, similar to CP-Ti [41]. Khan et al. [194] studied the corrosion behaviour of CP-Ti, Ti–6Al–4V, Ti–6Al–7Nb and Ti–13Nb–13Zr in PBS at various pH levels as well as in the presence of protein solutions. It was shown that the Ti–13Nb–13Zr alloy was least affected by the change in the pH level, in comparison to the other two alloys. The decrease in the hardness of the surface oxides as a result of corrosion in protein solutions was less for Ti–13Nb–13Zr than for Ti–16Al–7Nb and Ti–6Al–4V. Moreover, the α + β alloys had the best combination of corrosion and wear resistance but the tribocorrosion was less intense for the Ti–6Al–7Nb alloy than for the Ti–6Al–4V alloy and CP-Ti.

**Figure 14:**
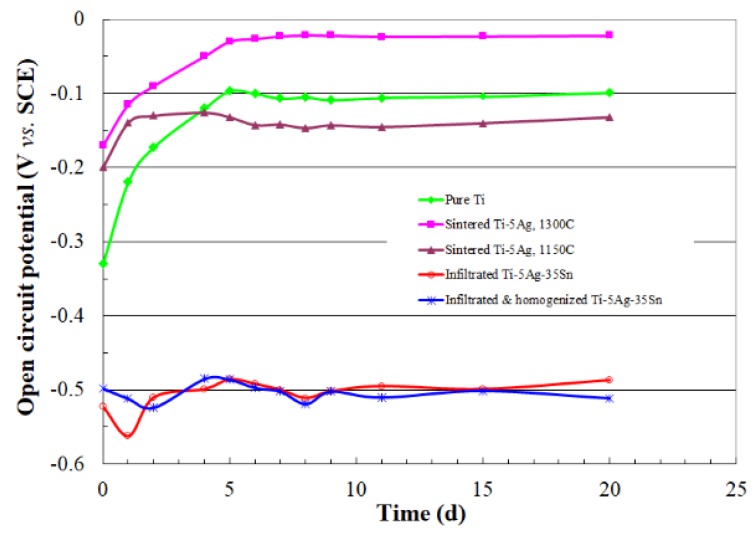
The time dependence of the OCP in saline solution at pH 5.5, T = 37 °C, of CP-Ti as well as of Ti-5Ag and Ti-5Ag-35Sn alloys processed by three-dimensional printing [187]. Reprinted with permission from Elsevier.

The following example is from one of the first corrosion studies, if not the first one, of Ti alloys prepared by *3D printing* (also known as *additive manufacturing*, AM). 3D Printing offers a variety of benefits over traditional manufacturing processes, including the ability to manufacture new designs and complex geometries, customization to customer requirements, improved performance, reduced weight, less generated waste, less energy consumption, higher efficiency, faster and cheaper manufacturing. Figure 14 shows the time dependence of the OCP of Ti–5Ag and Ti–5Ag–35Sn (wt%) alloys prepared by 3D printing followed by liquid-phase sintering or liquid tin infiltration (in order to densify the printed alloys). The OCP of pure Ti control sample is also shown, for comparison. The test solution was non-deaerated saline (0.9% NaCl, pH 5.5) at 37 °C. From Figure 14 it is evident that the OCP of both pure Ti and Ti–5Ag alloys increases over time (i.e., they become nobler due to formation of a stable passivation layer). Because the oxygen reduction rate on titanium is much lower compared to any other oxidizer, about a week is required for the reduction reaction to attain steady state. The addition of Ag affects the steady-state passivation behaviour. Over the whole 20-day period, the Ti–5Ag alloy sintered at 1300 °C shows the less negative OCP among all five materials. In principal, the Ti–Ag system could be regarded as galvanic coupling between a passive metal and a noble metal. Typically, coupling of Ti with noble metals such as Pt, Au or Pd results in spontaneous passivation, since these metals act as good catalysts (i.e., have higher *j*_0_) for the hydrogen and oxygen reduction (cathodic) reactions. The inclusion of 5 wt% Ag (*E*^0^ = +0.799 V vs. SHE) is thus sufficient to increase the corrosion potential of Ti–Ag. Because sintering at 1300 °C results in a more chemically homogeneous material with lower porosity compared to sintering at 1150 °C, the effect of Ag on the corrosion potential is more pronounced for the former alloy. In the case of pure Ti, as the surface oxide layer becomes thicker, the kinetics of mass transport through the layer becomes slower, thus resulting in a decrease in the passivation current density. On the other hand, Ag enhances the oxygen anion uptake in the *n*-type oxide layer on the Ti–Ag alloy, thus consuming oxygen anion vacancies. This vacancy deficit could result in a reduced passivation current density. If the solution was aerated, the combination of a more significant contribution of the hydrogen reduction reaction and corrosion potential increase due to the presence of Ag could have stabilized the Ti–Ag alloy within the passivity region. Thus, the positive effect of Ag addition could have been even more distinct. The Ti–Ag–Sn alloys also exhibit a galvanic couple behaviour, but it is much different from that observed in the Ti–Ag system. At a constant potential, the cathodic polarization curve of Sn typically exhibits a lower current density, while the anodic polarization curve of Sn exhibits a much higher current density, compared to Ti. Therefore, the corrosion potential is likely determined by Sn anodic reaction and Ti cathodic reaction. Consequently, the Ti–Ag–Sn alloys do not exhibit any passivation before Sn dissolution is significantly increased. The sintered Ti–Ag alloys were also found to exhibit an exceptionally high hardness, which may be beneficial in providing enhanced wear resistance. The combination of good electrochemical and mechanical properties and the presence of an antibacterial agent (Ag), make these alloys attractive candidates for both orthopedic and dental applications [187,195,196].

**Figure 15:**
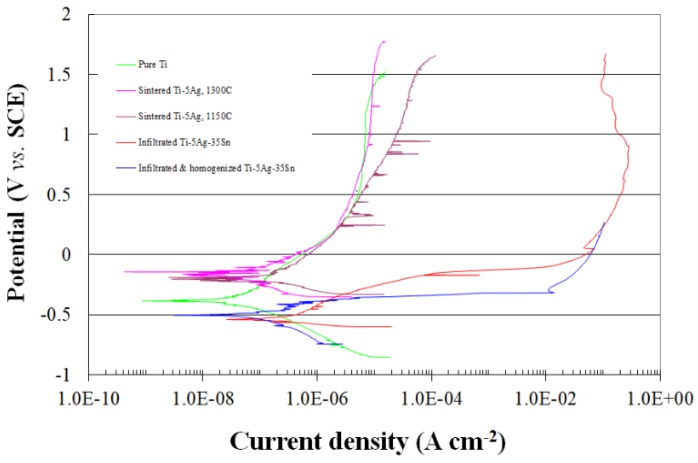
Potentiodynamic polarization curves in saline solution at pH 5.5, T = 37 °C, of pure Ti as well as of Ti–5Ag and Ti–5Ag–35Sn alloys processed by three-dimensional printing [187]. Reprinted with permission from Elsevier.

Figure 15 shows the potentiodynamic polarization curve that was measured following the OCP transient that is shown in Figure 14. From this figure it is evident that the curves for the pure titanium control sample and for the Ti–Ag alloy sintered at 1300 °C are similar in shape, although the Ti–Ag alloy exhibits a less active *E*_corr_. The Ti–Ag alloy sintered at 1150 °C, however, exhibits a less stable passivation (or metastable pitting) at high potentials. The Ti–Ag alloys sintered at either 1300 °C or 1150 °C exhibit higher *j*_corr_ values compared to the titanium control samples, although of the same order of magnitude. From this figure it is also evident that the corrosion potential is much more active and the corrosion current density is higher for the Ti–Ag–Sn alloys, either in the as-infiltrated or in the infiltrated and homogenized conditions [187,195,196].

The corrosion behaviour of different titanium alloys in different environments has been widely studied. Nakagawa et al. [197] studied the corrosion behaviour of Ti–6Al–4V, Ti–6Al–7Nb and Pd-containing titanium-based alloys in a wide range of pH and fluoride concentrations. The Ti–0.2Pd alloys were found to be more corrosion resistant due to surface enrichment with Pd. Williams et al. [143] studied the effect of proteins on the corrosion rates of CP-Ti and Ti–6Al–4V in the static as well as in the fretting modes. It was found that proteins increased the corrosion rate of CP-Ti but did not have an effect on Ti–6Al–4V. In the fretting mode, however, proteins did not have an appreciable effect on these two materials. Aziz-Kerrzo et al. [198] compared the corrosion susceptibility of CP-Ti, Ti–6Al–4V and Ti–45Ni in PBS using anodic polarization and electrochemical impedance spectroscopy (EIS) measurements. CP-Ti and Ti–6Al–4V were found to be more resistant to pitting corrosion in comparison to the Ti–45Ni SMA, for which pitting potentials as low as +250 mV versus SCE were recorded. Grosgogeat et al. [199] measured the galvanic corrosion current and potential for various couplings of either CP-Ti or Ti–6Al–4V with seven different Au-, Ag-, Pd- or Co–Cr-based dental alloys. In all cases, the level of corrosion was found low. 

### 5.4. Nitinol Shape Memory Alloy (SMA)

The shape memory effect (SME) was discovered in the early 1960s by Buehler and his co-workers at the U.S. Naval Ordnance Laboratory in an equiatomic alloy of nickel and titanium (50 at% Ni–50 at% Ti or 55 wt% Ni–45 wt% Ti). This alloy was thus named Nitinol (Nickel-Titanium Naval Ordnance Laboratory). The first attempts to evaluate the potential of NiTi as an implant material were made by Johnson and Alicandri in 1968. However, the use of NiTi for medical applications was first reported only in the 1970s. Although in the early 1980s some orthodontic and orthopedic applications were already available on market, it was only in the mid-1990s that the first commercial stents became widely used in medicine [75].

What makes the NiTi SMA unique is its ability to exist in two different reversible crystalline phases in its solid state at clinically useful temperatures: the austenite (A) phase prevails at a higher temperature, whereas the martensite (M) phase prevails at a lower temperature. A gradual transition between the A and M phases takes place between these two temperatures. The phase transformation takes place by shear lattice distortion. Each martensite crystal has two different forms, called twinned martensite and detwinned martensite. When martensite NiTi is heated, it starts transforming into austenite. The temperature at which this transformation begins is called austenite start temperature, *A*_s_. The temperature at which this transformation is completed is called austenite finish temperature, *A*_f_. When austenite NiTi is cooled, it starts transforming to martensite. The temperature at which this phenomenon begins is called martensite start temperature, *M*_s_. The temperature at which martensite is completely reverted to martensite is called martensite finish temperature, *M*_f_. The temperature range for the martensite-to-austenite transformation that occurs upon heating is somewhat higher than that for the reverse transformation upon cooling. The difference in the transition temperatures upon heating and upon cooling is called hysteresis. In practice, an alloy designed to be completely transformed by body temperature upon heating (*A*_f_ < 37 °C) would require cooling to about 5 °C to fully retransform into martensite (*M*_f_). Composition and metallurgical treatments have intense effects on these transition temperatures [75,200,201,202,203,204,205,206].

With respect to real biomedical applications, NiTi can have three different forms: martensite, stress-induced martensite (superelastic) and austenite. Martensitic NiTi is soft and ductile and can be easily deformed, superelastic NiTi is greatly elastic (rubber-like) and austenitic NiTi is rather strong, hard and rigid (similar to titanium). The NiTi alloy combines all of these properties, their exact manifestation being dependent on the temperature at which it is used. The SME, superelasticity and good damping properties make the NiTi SMA an attractive material for surgical applications, such as self-locking, self-expanding and self-compressing implants [75,200,201,202,203,204,205,206]. 

**Figure 16:**
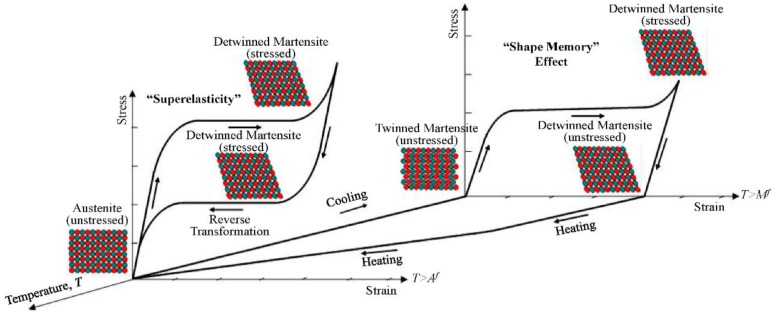
Stress–strain–temperature diagram of SMAs, illustrating both the shape memory and superelasticity phenomena [203]. Reprinted with permission from MDPI AG.

The term *shape memory effect* (SME) reflects the ability of an alloy to revert its original shape following deformation at low temperatures and subsequent heating above its transition temperature. This effect is based on the temperature-dependent austenite-to-martensite phase transformation on an atomic scale. As can be seen in Figure 16, the SME occurs when the SMA is deformed in the twinned martensite phase and unloaded while at a temperature below *M*_s_. While having this martensitic phase, the SMA can accommodate a significant amount of plastic strain which results from the low stress plateau, and then displays high residual strain upon unloading. Subsequently, when the SMA is heated above *A*_f_, it will recover its original shape by phase transformation from detwinned martensite to austenite. The SMA will regain the twinned martensite structure upon cooling, thus completing one cycle of phase transformation due to the SME [75,200,201,202,203,204,205,206]. 

SMAs may have two different kinds of SME: one-way shape memory effect (OWSME) and two-way shape memory effect (TWSME). A two-way SMA (TWSMA) exhibits the memory of both a high-temperature shape in austenite (hot shape) and a low-temperature shape in martensite (cold shape) and can be cycled reversely and spontaneously between these two shapes by simply changing its temperature. In comparison with one-way SMA (OWSMA), where an additional element such as a spring is needed to provide an external force to reset the cold shape upon cooling, TWSMA allows a more compact and simplified configuration of smart actuators and structures. This may be of advantage to industrial and surgical applications where a limited working space often exists. The TWSME is not intrinsic to SMA but may exist after specific thermomechanical treatments known as training procedures. Shape memory treatment is the final process of Nitinol SMA fabrication. The most common treatment is the so-called “medium temperature treatment” (a temperature range of 350 to 450 °C, holding time ranges from 10 to 100 min, depending on the product size). Alternatively, an aging treatment, known as “training process,” is often used for TWSME [75,200,201,202,203,204,205,206].

Nowadays, most self-expanding implants such as stents and filters employ the thermal SME of Nitinol to allow deployment into the body. The implant is usually compressed at low temperature to fit into a delivery catheter. It is not essential to retain the implant cold during introduction into the body since the implant remains constrained inside the delivery catheter to avoid any early release. The original shape of the implant is restored when it is released and reaches body temperature [75,205,206].

The superelasticity (SE or pseudoelasticity) phenomenon, also illustrated in Figure 16, is caused by a stress-induced transformation and represents a nonlinear deformation behaviour with almost zero-residual strain of SMAs at temperatures above the *A*_f_ temperature. By deforming the austenite, stress-induced martensite is formed. The martensite reverts to austenite once the stress is removed. Within a given temperature range, NiTi can be strained sometimes more than conventional alloys without being plastically deformed (namely, it can reach elastic deformations of 10–12%). It has high yield strength and low *E*, that is, high resilience. It can be loaded at 4 times yield strength and still run 10 cycles without failure [75,200,201,202,203,204,205,206].

Nitinol thus has remarkable advantages in applications requiring kink resistance, flexibility, crush resistance, constancy of applied stress and large expansion or deformation ratios. Most Nitinol stents are superelastic at body temperature and can be crushed fully flat and still recover their original shape. Superelasticity is a very important feature in the treatment of superficial vessels, such as carotid and femoral arteries subjected to external crushing. To date, the most successful medical applications of Nitinol using the superelasticity property are guidewires, baskets, snares, needles, coils, soft tissue anchors, intramedullary canal reamers, anastomotic devices, self-expanding stents and stent-grafts, filters and occlusive distal protection devices and several catheters used for radiofrequency ablation, brachytherapy, atherectomy, thrombectomy and laser therapy [75,205,206].

Relevant ASTM standards include F2005 (terminology related to SMAs) [207] and F2063 (wrought alloys) [208]. In recent years, Nitinol and other SMAs have been produced by different AM processes [209,210,211]. 

Some unique properties of NiTi may be valuable in surgery. NiTi is capable of being vastly damped and vibration-attenuated below *A*_s_. In orthopedics, this property can be useful, for example, for dampening the peak stress between bone and an articulating implant. The low elastic modulus of NiTi (*E* ≅ 40 GPa, much similar to that of bone than any other metallic biomaterial) may also be beneficial. NiTi possesses enhanced fatigue and ductility properties, as well as very high wear resistance. It is nonferromagnetic and has lower magnetic susceptibility than stainless steel; therefore, it generates less artefacts during MRI examination [75]. The mechanical properties of NiTi are very sensitive to the chemical composition and the thermomechanical history. A typical chemical composition is provided in Table 6, whereas typical mechanical properties are given in Table 7.

Due to the high nickel content of NiTi, a concern has been raised that nickel ions might be released from this alloy due to corrosion and cause undesirable effects. Fortunately, however, the surface of NiTi consists mainly of TiO_2_, smaller amounts of NiO and Ni_2_O_3_ and metallic Ni, while nickel-titanium constitutes the inner layer. Because its surface oxide is primarily TiO_2_, Nitinol is sometimes regarded as a Ti alloy [45]. The thickness of the oxide layer is between 2 and 20 nm. During implantation, this oxide layer grows and picks up minerals (e.g., CaPs) and other constituents of biofluids, which results in remodelling of the surface. Several surface treatments have been employed to enhance the corrosion properties of NiTi, including titanium nitride coating processes by an arch ion plating method, chemical modification with human plasma fibronectin via aminosilane and glutaraldehyde as coupling agents, plasma-polymerized tetrafluoroethylene (PPFTE) coating, laser surface treatment, electropolishing and nitric acid passivation [75].

Most of the information on the corrosion behaviour of NiTi stems from investigations of dental arch wires under in vitro conditions. Although more studies and for longer exposure periods, are still necessary, the resistance of NiTi to pitting corrosion is apparently similar to (or better than) 316L stainless steel [212]. Ryhänen [75] reviewed the literature and added his own data to demonstrate that despite its higher initial nickel dissolution, NiTi induces no toxic effects, decrease in cell proliferation or inhibition in the growth of cells in contact with the metal surface. The muscular tissue response to NiTi was clearly non-toxic and non-irritating, as were also the neural and perineural responses. The overall inflammatory response and the presence of immune cells, macrophages and foreign body giant cells were similar to those around 316LVM stainless steel, CP-Ti and Ti–6Al–4V. After eight-week implantation, histomorphometry revealed that the fibrous capsule around NiTi was thicker than around stainless steel. However, at 26 weeks post-surgery, the capsule thickness was the same. Determination of trace metals in several distant organs showed no statistically significant differences in Ni concentration between the NiTi and 316LVM stainless steel. Thus, Ryhänen concluded that the biocompatibility of NiTi is similar to or better than that of stainless steel or Ti–6Al–4V alloy. Yet, it was recommended that when NiTi is intended to be used in long-term implants, an optimal surface treatment should be considered [75].

Some surface modification processes have been shown to improve the corrosion performance of Ni–Ti alloys and avert nickel dissolution. These include titanium nitride coating and chemical modification with coupling agents to improve the corrosion resistance. Nevertheless, when the coating on a Ni–Ti alloy is damaged, corrosion is apparently accelerated compared to an uncoated alloy. Laser surface treatment of Ni–Ti leads to increase in the superficial titanium concentration and thickness of the oxide layer, improving its cytocompatibility to that of CP-Ti. Electropolishing and nitric acid passivation can improve the corrosion resistance of Ni–Ti alloys thanks to the enhanced uniformity of the oxide layer [213].

### 5.5. Dental Amalgams

Dental amalgams, in widespread use for over 150 years, are among the oldest materials used in oral healthcare. They are still the most useful restorative material for posterior teeth. They are formed by mixing liquid mercury (45–55 wt% Hg) and a powder made of Ag, Sn and Cu (sometimes–also smaller amounts of Zn, Pd, In or Se). Mixing occurs via mechanical vibration and results in a putty-like material, which is easy to manipulate (e.g., in filling cavities). Dental amalgam has been broadly used as a direct filling material due to its advantageous mechanical properties along with low cost and easy placement. Amalgams possess high compressive strength and high dimensional stability; however, shrinkage and corrosion remain of concern [214].

The oral environment triggers corrosion. Mouth is permanently moist and is routinely subjected to temperature variations. Food and liquids ingested have a wide pH range. Acids are released during the breakdown of foodstuffs. Dental alloys with high contents of Au and other precious metals, used for crowns and inlays amalgams, have high corrosion resistance in almost all oral environments. An exception may be when high fluoride levels develop in the mouth during some dental cleaning procedures. In some patients, both Ag- and Au-based alloys might undergo *tarnishing*, in which a thin black layer (most likely a sulfide) grows across the surface [36]. In the oral cavity, tarnish frequently results from the formation of hard and soft deposits on the surface of the restoration. The soft deposits are plaques and films consisted mostly of microorganisms and mucin. Surface discoloration may also occur on a metal due to the formation of thin films, such as oxides or sulfides. Tarnish is an early sign of corrosion. Amalgam restorations often tarnish and corrode in oral environment. Corrosion of dental amalgam may establish galvanic couples. Ion release due to corrosion is of greatest importance. Humans are exposed to mercury and other dental metals via vapor or corrosion products in swallowed saliva and also by direct absorption into blood from oral mucosa. In recent decades, the use of dental amalgam has been considered in regard to potential toxicity of mercury components. The main toxic effects are said to be neurotoxicity, kidney dysfunction, reduced immunocompetence, effects on oral and intestinal bacterial flora, foetal and birth effects and effects on general health. The clinical challenge is that corrosion often takes place below the surface of restorations. Evaluating the internal corrosion state of an amalgam restoration is challenging for current clinical diagnostic tools and techniques. Instead, alleged recurrent decay is the dominant reason for replacing amalgam restorations [214].

Several sulfides, including hydrogen sulfide or ammonium sulfide, corrode silver, mercury and other metals that exist in amalgam. Water, oxygen and chloride ions are present in saliva and accelerate the corrosion attack. Various acids such as phosphoric, acetic and lactic are sometimes present. At the right concentration and pH, these can result in corrosion. Particular ions may play a key role in the corrosion of certain alloys. For example, oxygen and chlorine are involved in corrosion of amalgam at the tooth interface and within the bulk of the alloy [214]. 

Because dental amalgams are multiphase alloys, localized, galvanic or intergranular corrosion between the different phases might develop [214,215]. In the conventional silver amalgams, the Ag-containing phases are nobler and accelerated corrosion attacks the γ_2_ phase (Sn_7_Hg). Corrosion results in the formation of tin oxychloride from the tin in the γ_2_, while releasing toxic mercury to the body. The reaction of the released mercury with unreacted γ can form more γ_1_ and γ_2_ [214]. On the other hand, the high-copper amalgams do not contain any γ_2_ phase in the final set mass. Although these amalgams are also susceptible to intergranular corrosion, the most susceptible phase is η′ (Cu_6_Sn_5_), which does not release Hg into the body when it corrodes [216]. Yet, because the high copper content shifts *E*_corr_ to more positive values, high Hg release might still be observed. Electrochemical tests of pure phases have revealed that the Ag_2_Hg_3_ phase has the highest corrosion resistance, followed by Ag_3_Sn, Ag_3_Cu_2_, Cu_3_Sn, Cu_6_Sn_5_ and Sn_7–8_Hg. The following compounds have been detected on dental amalgams in patients: SnO, SnO_2_, Sn_4_(OH)_6_Cl_2_, Cu_2_O, CuCl_2.3_Cu(OH)_2_, CuCl, CuSCN and AgSCN [214]. A galvanic shock is well known in dentistry. A post-operative pain due to galvanic shock can rarely be a real source of discomfort to the patient [214]. 

Joska et al. [217] observed that the Hg release rates from conventional silver amalgams were similar to those from high-copper amalgams, with the method of preparation being acute. Moreover, it was shown that, for most people, the main source of Hg uptake is food, with less than 10% originating from dental amalgams [218]. High-copper amalgams are processed from either a mixture of silver-tin and silver-copper alloys or from a ternary silver-copper-tin alloy. The high-copper amalgams have superior clinical properties, including a higher corrosion resistance [219]. Nevertheless, too much copper might cause a positive shift in the corrosion potential, resulting in increased corrosion rates [217].

### 5.6. Gold

Gold and its alloys are used in applications such as electrode materials in medical devices and dentistry due to their durability, immunity to corrosion, desired electrical and thermal properties and aesthetic appearance. Alloying of gold with copper or platinum results in increased strength, while silver is added to compensate for the colour of copper. Although gold is common in dental cast restoration applications, it is not suitable for orthopedic applications due to its high density, poor strength and high cost.

Under most environments, gold is immune to corrosion. The Pourbaix diagram for gold in aqueous solutions free from complexing substances at 37 °C shows that, except of a small regime at pH < 1, gold is immune to corrosion over the entire domain of water stability. However, the introduction of a small amount of chloride ion into the system allows for regions of stability of soluble gold(I)-chloride complexes, thus making it prone to corrosion. In the presence of 0.1 M Cl^−^ (and 0.6 μM Au^+^), the Pourbaix diagram shows that the domain of gold corrosion extends significantly to pH as high as 8, although at 6 < pH < 8 it does not overlap the region of water stability and thus requires the application of voltage perturbation [220]. With respect to the valence of gold ions, Frankental and Siconolfi [221] found that gold dissolves as a monovalent ion at *E* < 0.8 V and as a trivalent ion at *E* > 1.1 V. Gold(I)-chloride is exceptionally unstable in aqueous environments. Analysis of antibody sensitization indicates that AuCl quickly decomposes in vivo into AuCl_3_ [222]. From kinetics point of view, gold exhibits a typical passive metal behaviour in the presence of chloride. Gold corrosion rate in the presence of chlorides is a strong function of both chloride concentration and solution velocity. These effects are attributed mainly to a depletion of chloride ions at the anodic surface, leading to a diffusion-limited current density. This suggests that the corrosion rate is unlikely to be significantly affected by cathodic reactions [220]. 

Rosenberg [220] studied the electrochemical performance of gold membranes covering drug-filled wells in implantable drug-delivery devices. Comparisons were made between behaviours in PBS, calf serum and a rat model. Results showed that PBS solutions can match the thermodynamic environment of biological media but not the kinetics. The kinetics of gold corrosion in calf serum was shown to be limited significantly by modification of the gold surface by the media. Gold samples treated in serum and subsequently corroded in PBS exhibited similar kinetics to those corroded in serum alone. It was suggested that the kinetic variation was primarily due to protein modification of the gold surface. Gold samples corroded in serum exhibited protein hydrogels. Gold chloride was shown to prevent serum from modifying the gold surface.

### 5.7. Metallic Glasses

Attempts to find substitutes to conventional biometals led to the discovery of bioglass in the early 1970s [223]. Unfortunately, most bioglasses developed so far are not real alternatives to biometals, because they have inadequate mechanical properties and are thus improper for clinical applications that require placement in load-bearing sites [224].

*Metallic glasses* (MGs), also known as glassy metals or amorphous metals, were first reported by Klement et al. in 1960, for the case of Au_75_Si_25_ [225]. MGs have a unique combination of physical, chemical and mechanical properties. For example, their amorphous structure and concomitant lack of dislocations and associated slip planes results in high strength and elasticity. The homogeneous chemical composition and absence of grain boundaries result in better corrosion resistance, in particular in certain compositions that are associated with segregation of passive layer-forming elements to the surface.

Yet, the limited sample sizes initially achievable (in the range of microns) significantly limited their use. These limitations were overcome by the development of *bulk metallic glasses* (BMGs) with much lower critical cooling rates (<100 K·s^–1^) [224]. BMGs have excellent formability in the supercooled liquid region. 

**Figure 17:**
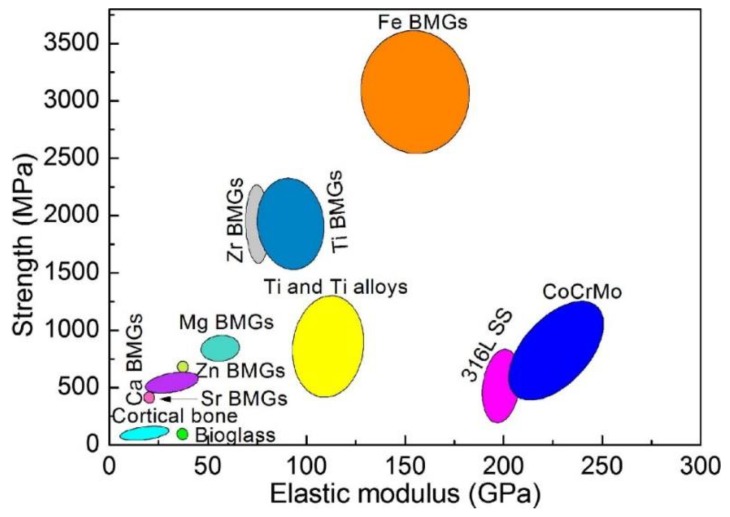
Ashby diagram comparing the mechanical properties of conventional bioglasses, biometals and the novel biomedical BMGs [224]. Reprinted with permission from Elsevier.

Figure 17 is an Ashby diagram comparing the compression strength and Young’s modulus values of biometals, bioglasses and BMGs. It is evident that bioglasses have low strength and low Young’s modulus, while conventional metallic biomaterials have high strength and high Young’s modulus. In contrast, biomedical BMGs, which combine the properties of bioglasses and biometals, have high strength and low elastic modulus. Particularly, the tremendously high elastic limit of 2% typical for biomedical BMGs compares well with the value of ∼1% reported for bone. This suggests that biomedical BMGs are unique in their ability to flex elastically with the natural bending of the bones and would thus distribute stresses more uniformly than other biomaterials in use, minimizing stress concentrations, reducing stress shielding effects and consequently accomplishing faster healing rates. Thanks to the unique properties of biomedical BMGs, BMG bone screws can have a thinner shank and deeper threads, thus yielding greater holding power to the fracture bones [224].

**Figure 18:**
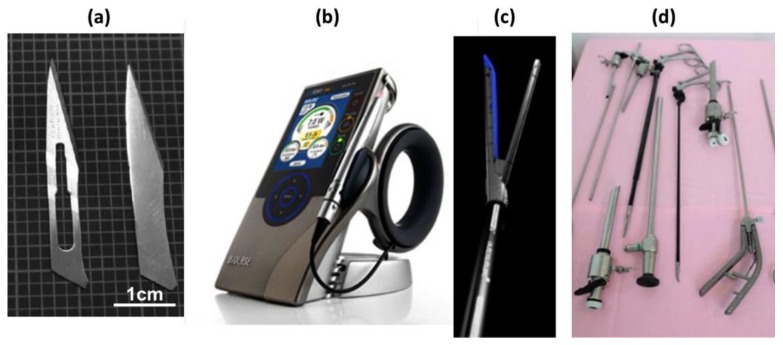
Medical devices made out of BMGs. (**a**) Commercial martensitic steel surgical blade coated with ZrCuAlAgSi BMG film (left) and ZrCuAlAgSi BMG surgical blade (right). (**b**) The ezlase diode dental laser system, from Biolase Technology, uses a glassy metal in its housing. (**c**) BMG medical stapling anvils. (**d**) Amorphous alloys in minimally invasive medical devices [224]. Reprinted with permission from Elsevier.

In the last decades, various BMGs have been developed specifically for biomedical applications and both in vitro and in vivo tests have been carried out to assess their practicability as biomaterials. Some of the biomedical devices that have been developed using BMGs are shown in Figure 18. These include surgical blades with improved sharpness (Figure 18a), the housing of a handheld pen laser for dental procedures (Figure 18b), medical stapling anvils (Figure 18c) and minimally invasive medical devices (Figure 18d). Besides the aforementioned medical devices, BMGs are favourable contenders as biomedical implants, including cardiovascular stents, orthopedic and dental implants [224].

There are several families of metallic glasses of potential use as biomaterials. The first family is obviously Ti-based metallic glasses. Among these, some contain nickel and/or beryllium and will not be mentioned here, because I do not find them of real potential use. Others include the Ti–Zr–Cu–Pd, Ti–Zr–Cu–Pd–Sn, Ti–Zr–Hf–Cu–Ni–Si–Sn, Ti–Zr–(Ta/Pd)–Si, Ti–Zr–Si, Ti–Si, Ti–Nb–Si and Ti–Zr–Nb–Si systems [224,226,227,228,229,230,231,232,233,234,235]. Ti-based BMGs have fairly low Young’s modulus (80–120 GPa), high fracture strength (1700–2500 MPa), low density, excellent specific strength and high wear resistance [224].

With respect to corrosion behaviour, Ti-based BMGs have shown a passive behaviour at OCP, with a low corrosion rate. Their pitting corrosion resistance is statistically equivalent to or better than, the conventional crystalline biomedical alloys, including 316L stainless steel, CP-Ti and Ti-based alloys such as Ti–6Al–4V [224]. Researchers have studied the biocompatibility of Ti-based BMGs, both in vitro and in vivo. Results have demonstrated that Ti-based BMGs are more biocompatible than the conventional crystalline Ti–6Al–4V and Ti–45Ni alloys [224]. It should be noted that copper has been reported to have a critical role in the initiation of significant pitting events in MGs. The pitting potential of the Cu-containing MGs is shifted to lower values when the Cu-content is increased. Hence, the Cu-containing glassy alloys not only show cytotoxic effects in the human body environment but also their limited corrosion resistance in SBFs was recognized as a critical matter in lifetime prediction [226]. 

A second family of MGs for biomedical applications is the Zr-based [224,230,236]. Examples include the Zr–Cu–Ni–Al, Zr–Ti–Ni–Cu–Be, Zr–Al–Ti–Cu–Ni and Zr–Cu–Fe–Al systems. The biomedical Zr-based BMGs have high hardness (about 2–3 times that of conventional crystalline 316L stainless steel, Ti-based alloys and Zr-based alloys) and high yield strength that is significantly higher than that of these crystalline metallic biomaterials. The Zr-based BMGs also show a noticeably large plastic strain, elastic strain around 2% and low modulus of 70–80 GPa. The latter is lower than that of crystalline 316L and Ti–6Al–4V. The high strength of the Zr-based BMGs allows the manufacturing of thinner struts for cardiovascular stents, which benefits its deliverability and reduces the rate of restenosis. Compared to traditional 316L stainless steel stents, biomedical Zr-based BMG stents would require only 1/3 of the cross-section of the strut and would have more than five times the deflection. It should also be noted that the high elastic limit and low Young’s modulus would allow a more uniform distribution of stresses, compared to existing materials, thus minimizing stress concentrations, reducing stress shielding effects and consequently achieving faster healing rates [224].

Corrosion tests in a variety of SBFs have revealed that biomedical Zr-based BMGs have a lower passive current density and a much higher pitting potential compared to crystalline 316L stainless steel, Zr and Zr-based alloys and Ti and Ti-based alloys. This suggests that the passive films formed on the Zr-based BMGs are more protective than those on the aforementioned control groups, thus providing increased corrosion resistance. Adding Nb and Ag is beneficial for corrosion resistance, especially with respect to superior pitting corrosion resistance. The biocompatibility of Zr-based BMGs is also better than that of the crystalline metal counterparts [224].

A third family of BMGs is the Fe-based. Fe-based BMGs are cheaper than Ti-based or Zr-based BMGs, making them potentially attractive for any large-scale biomedical application. They also have practically good glass forming ability (GFA) and can be easily prepared by traditional Cu-mould casting/cooling methods. Since the first synthesis of Fe-based BMG in the Fe–Al–Ga–P–C–B system in 1995, a variety of Fe-based BMGs have been developed, including the Fe–(Zr,Hf,Nb)–B, Fe–Ga–(P,C,B,Si) and Fe–Co–Ln–B systems. In the last material, the lanthanide metal, Ln, is Tm or Er, for example. Unfortunately, most of these BMGs are soft magnetic materials and are thus not suitable for biomedical applications. Nonmagnetic Fe-based BMGs were first developed in 2003 and were termed “amorphous steel,” because their composition is similar to that of stainless steel (i.e., they contain Fe, C, Cr and Mo), for example Fe_51_Cr_18_Mo_7_B_16_C_4_Nb_4_. Various investigations have shown that the Vickers hardness of Fe-based BMGs is in the range of 1200–1800 MPa and that their tensile fracture strength is at least 3,000 MPa. These values far exceed those reported for state-of-the-art steels. In addition, these Fe-based BMGs have better MRI compatibility compared to 316L stainless steel [224].

The Fe-based BMGs have higher pitting potential, lower corrosion current density and quite lower ion release compared to 316L, along with good biocompatibility in vitro. Fe_51_Cr_18_Mo_7_B_16_C_4_Nb_4_ shows higher polarization resistance than that of 316L and Ti–6Al–4V [224].

In many clinical cases, for example, bone fracture and cardiovascular diseases, temporary implant materials are required. In this case, biodegradable materials are the optimal choice, as they function while healing takes place and a new tissue forms and degrade in vivo thereafter. The biodegradable BMG family includes Ca-, Mg-, Sr- and Zn-based BMGs [224]. These biodegradable BMGs, however, will not be discussed here. The reader is referred to Ref. [224] for a review of these materials. BMG composites and foams are also described in Ref. [224], along with routes for future improvement of biomedical BMGs, their surface modification and metallic glass coatings. The latter are attractive candidates for the coating of metal implant bearing surfaces, for example in TJR. Indeed, Tesk et al. [237,238] suggested a while ago glassy Co–P, Co–Cr–C and Cr–C coatings for this purpose.

### 5.8. Biodegradable Metals

The common paradigm of metallic biomaterials entails good corrosion resistance in vivo. However, a new class of degradable materials—the so-called “biodegradable metals”—has been breaking this paradigm in recent years [239,240]. Biodegradable metals can be defined as metals expected to corrode gradually in vivo, with an appropriate host response stimulated by released corrosion products and then dissolve entirely upon achieving the goal of promoting tissue healing, with no implant residues. Thus, the main characteristics of a biodegradable metal are right degradation rates and degradation modes in vivo and metallic elements that can be metabolized by the human body [239].

Interest in Mg-based alloys for biomedical applications has been rapidly growing. But, Mg was suggested as a biodegradable implant material already in 1878, in a report about absorbable ligature for the closure of bleeding vessels. Pure iron had been used in implants long before. The first studies on biodegradable metals in the musculoskeletal field were published at the beginning of the last century based on experience with osteosynthesis implants made of Mg alloys. Metal allergies were not accounted for due to the more significant surgical complications at that time, such as infection or implant failure. Skin sensitizing reactions to metal implants were described more often after the introduction of aseptic surgery and less corrosive osteosynthesis materials, such as stainless steel; they are still apparent clinically in 10–15% of all implanted metals. In contrast, biodegradable Mg alloys have demonstrated no skin sensitizing potential in animal studies. A higher tensile strength and a Young’s modulus that is closer to that of bone make biodegradable metals advantageous for load-bearing applications compared to other biodegradable materials such as polymers, ceramics or bioactive glasses [43].

During the last 20 years the Mg alloy technology has been advancing and both the mechanical and corrosion properties have been improved. Hence, the interest in degradable metals as temporary implant materials has been growing. To this aim, various fundamental issues related to biodegradable metals have been investigated, for example, the selection of alloying elements, tuning of microstructural and mechanical properties, biodegradation mechanisms and the factors affecting them, control of degradation rates and ion release profile and in vitro and in vivo biocompatibility of biodegradable metals [239,240].

The control and adaptation of the implant degradation rate is vital, since the resorption capacity of the tissue is limited. Moreover, the local physiology of the implant environment governs the maximal degradation rate of a temporary implant [43]. There are many factors that contribute to the corrosion of metals when implants are placed in vivo. For Mg corrosion, the most acute factor is apparently the local pH, while Fe corrosion seems to be mainly dependent on the local oxygen concentration. Following surgery, the pH around the implant is lowered to a value of 5.3–5.6, mainly because of trauma. This process might initially accelerate the Mg corrosion, while infectious microorganisms and crevices formed between components can reduce the local oxygen concentration. Mg corrosion is commonly too fast in vivo, while the foremost challenge in Fe implants is to accelerate corrosion. A possible design of appropriate Mg alloys would decrease the initial implant corrosion by the right alloying elements, microstructure, processing, and/or coating. However, it should be borne in mind that the corrosion rate would be further reduced in vivo following implantation because of adherent proteins and inorganic deposits such as CaPs. If so, a Mg implant with an initially lowered corrosion rate could eventually lead to an arresting corrosion process in vivo. Therefore, the right balance between reduced corrosion rate and guaranteed complete corrosion in vivo will establish a valuable biodegradable Mg implant; otherwise, parts of the implant might be preserved locally, acting as long-term biomaterials [43].

Magnesium is essential to human metabolism and is the fourth most abundant cation in the human body. Moreover, magnesium is also a co-factor for many enzymes and stabilizes the structures of DNA and RNA [43]. Magnesium and its alloys are light metals having a density of about 1.74 g cm^–3^, which is 1.6 times less than aluminium and 4.5 times less than steel. The fracture toughness of Mg is higher than bioceramics such as HAp, while its elastic modulus and compressive yield strength are closer to those of natural bone compared to other commonly used metallic osteosynthesis materials. Moreover, magnesium has a low corrosion resistance, as evident from its very negative electrochemical standard potential, *E*^0^ = −2.363 V versus SHE. or alternatively *E*^0^ = −2.159 V versus SHE if magnesium oxides or magnesium hydroxides are formed [43]. According to Pourbaix [57,58], these potentials may be shifted if the Mg ions are complexed in the corrosion layer. It is evident that magnesium carbonates and phosphates are formed during Mg corrosion under cell culture conditions; they have also been detected in the complex corrosion layer in vivo. Hence, complexed Mg actually changes the local potential and stimulates pitting in vivo. When exposed to air, the surface of magnesium is passivated by a thin grey layer of magnesium hydroxide, which is further reduced by chemical reactions. Magnesium hydroxides are marginally soluble in water; however, severe corrosion takes place in saline SBF, as well as in vivo, where high chloride ion concentrations of about 150 mM exist. In water, corrosion-protective magnesium hydroxide forms on the surface, but when the chloride concentration in the corrosive environment is higher than 30 mM, it starts converting into greatly soluble magnesium chloride. Thus, severe pitting corrosion can be identified on magnesium alloys in vivo. Notably, Mg corrosion is rather insensitive to different oxygen concentrations around implants in different anatomical locations [43]. The overall corrosion reaction of magnesium in aqueous environments is

(28) $$\mathrm{Mg}+2H_{2}O\to \mathrm{Mg}{\left(\mathrm{OH}\right)}_{2}+H_{2}.$$

Magnesium corrosion can be determined in vivo using microtomography. Particularly, synchrotron-based microtomography (SRµCT) is a non-destructive method with a high density and high spatial resolution. Element-specific SRµCT allows determining the spatial distribution of the alloying elements during in vivo corrosion. The remaining non-corroded metal volume as well as the surface morphology can be determined by 3D non-destructive testing (3D-NDT) on a micrometre scale using SRµCT [43].

During the corrosion process, magnesium ions are released from the magnesium implant. These ions can be quickly removed from the body by the blood serum and the kidneys. Magnesium can also be stored in muscle (39% of total Mg) or bone (60%), which are the natural storages of the 21–35 g of elemental Mg of an average adult person who weighs about 70 kg. The concentration of magnesium ions in the ECF is maintained constant at 0.7 to 1.05 mM. While serum magnesium levels above 1.05 mM might result in muscular paralysis, hypotension and respiratory distress, cardiac arrest happens only at exceptionally high serum levels of 6–7 mM [43].

In the case of magnesium and its alloys, impurities and cathodic sites with a low hydrogen overpotential facilitate hydrogen evolution, thus causing considerable galvanic corrosion and potential local gas cavities in vivo. However, the reports of these gas cavities are ambiguous. Gas cavities have been observed following subcutaneous implantation, whereas intravasal application exhibited no local gas build-up. This finding may be explained in terms of the diffusion and solubility coefficient of hydrogen in biological tissues. The solubility of hydrogen in tissues is affected by the content of lipids and proteins and by salinity but in fat and oils, the solubility seems to be almost independent of temperature in the physiological range. Not only viscosity but also different tissue components and structures like lipids, proteins and glycosaminoglycans, influence the value of the hydrogen diffusion coefficient. Depending on experimental setup, the diffusion coefficient may be underestimated in both stagnant and flowing media due to a boundary layer formation, which increases the effective diffusion distance. This observation may be important for intravascular magnesium applications. Correlating the hydrogen diffusion coefficients in various biological media with fractional water contents from about 68% to 100%, it is found that the diffusion coefficient of hydrogen increases exponentially with the increasing water fraction of the tissue. The tissue water content in animals increases from adipose tissue to skin and from bone to muscles and humans are similar for the same tissue regardless of the species. This can explain why different corrosion rates and gas cavities were observed for Mg alloys in different anatomical implantation sites. The local blood flow and the water content of the tissue surrounding the implant are important parameters, which need to be taken into account when designing biodegradable Mg alloys with a proper corrosion rate. Concurrently, it can be anticipated that local hydrogen cavities occur when more hydrogen is generated per unit time than can be dissolved in the neighbouring tissue or diffuse from the implant surface into the extracellular medium, which is renewed depending on the local blood flow. This implies that Mg alloys corrode in vivo with an appropriate corrosion rate when no local gas cavities are observed during the implantation period in a specific anatomical site [43].

Magnesium corrosion in vivo can be controlled by alloying, processing and coating. The first empirical approach in biodegradable stent development has successfully led to Mg alloys containing rare-earth elements. However, a more systematic approach is necessary. For the use in humans, it may be better to select Al-free Mg-alloy systems, while Al-containing Mg-alloys may be used for research purposes, for example, to investigate corrosion control by certain coatings by measuring the release rate of aluminium [43].

A second type of biodegradable metals is based on iron. Iron is an essential trace element in the human body and due to its high toxic potential–its pathways are heavily regulated in mammals. Iron in its Fe^2+^ state can only be absorbed in the intestine. In contrast, iron in its Fe^3+^ state should be reduced before it can be absorbed. There are several other metals and ligands (e.g., oxalic acid), which form less soluble iron complexes and thus inhibit the iron absorption. Iron in its Fe^3+^ state is transported by the protein transferrin. In human blood, approximately 70% of iron is bound to haemoglobin; the rest is bound to myoglobin or is stored as ferritin or hemosiderin. Most of the iron released from the degradation of haemoglobin and myoglobin will be reused and only a small portion of ~10% will be excreted via faeces, urine or sweat. Haemoglobin contains Fe^2+^, forms an unstable reversible bond with oxygen in the erythrocytes (oxyhaemoglobin) and appears bright red, while the oxygen-unloaded form appears purple-blue and is called deoxyhaemoglobin. If the iron in the haemoglobin is oxidized, ferrihaemoglobin (methemoglobin) is formed, which cannot bind oxygen. In erythrocytes, the methemoglobin reductase reactivates haemoglobin (Fe^3+^ → Fe^2+^). Otherwise, haemoglobin will be enzymatically degraded and eliminated from the body [43].

Iron is generally bound to proteins and is greatly regulated in mammals; hence, there are several known iron storage diseases. Most of these diseases are based on genetic defects and result in the pathological storage of iron in liver, heart, skin, endocrine organs and the reticuloendothelial system. If the natural storage capacity of free iron is surpassed and transferrin cannot bind any more iron, then iron causes oxidative stress, which leads to cell and tissue damage and the excess iron will be pathologically stored in heart, liver and endocrine organs. In this case, free iron ions harm cells by forming reactive free radicals, for example, hydroxyl radicals, which have a very strong oxidation capacity. The hydroxyradical reacts with organic molecules on the cell membrane and initiates a lipid-peroxidation process, which eventually leads to cell death [43].

Thus, for biodegradable iron implants, it is most important that the local storage capacity and the amount of iron that is released per time interval do not exceed the local and general transport and storage capacities. Moreover, iron implants may need to be used only in individuals without any iron-related disease. However, the slow degradation of an iron stent that weighs ~40 mg will unlikely cause system toxicity, taking into account the high iron load of normal blood (about 447 mg L^−1^) [43].

During the last 5 years, interest has emerged in biodegradable zinc and Zn-based alloys as a third type of biodegradable metals for the fabrication of degradable stents and orthopedic implants [241,242,243,244,245,246,247,248]. This is due to their excellent biodegradability, bioabsorbability and adaptability to tissue regeneration [243] and because their corrosion rates are between those of iron and magnesium in physiological conditions. Zinc and magnesium are chemically similar, in that both elements have only one oxidation state (+2) and both cations are of similar size (0.74 and 0.72 Å, respectively). Zn^2+^ is well regulated within physiological systems and plays a critical role in numerous fundamental cellular processes [242,245]. Zinc is an antioxidant and an endothelial membrane stabilizer. The maximum daily intake of Zn is 10 mg per day for adults [245], although some variance may be found in the literature. Zn^2+^ released from an implant may suppress harmful smooth muscle cells and restenosis in arteries, while stimulating beneficial osteogenesis in bone [242]. In addition, it can inhibit acute inflammation and increase the expression of collagen and VEGF in colorectal tissues [244]. The toxicity of and tolerance to zinc and its alloys depend considerably on the type of biological cells and the concentration of zinc [244]. With respect to mechanical properties, porous zinc has recently been prepared using spark plasma sintering and exhibited both compressive yield strength and compressive modulus similar to those of trabecular bone [246]. It can thus be concluded that Zn-based biodegradable metals may become more attractive than magnesium and iron for the fabrication of certain medical devices. However, before this can happen, further research is required; for example, tailoring their corrosion rates and mechanical properties and characterize more comprehensively their biocompatibility and toxicity in different anatomical sites.

## 6. Implant Failure, Retrieval and Failure Analysis

Failures of implants are usually classified as either mechanical, electrochemical, biological or combinations of these. Mechanical failure mechanisms include micromotions [249,250,251], overload [252], fatigue [253] and wear [69,81]. Electrochemical failures are mainly related to different forms of corrosion. Biological failures result from infection [254,255], inflammation [255,256,257,258], enzymatic degradation, calcification [259] and so forth. Failures may also result from synergistic effects, for example–SCC, corrosion fatigue (CF) and fretting corrosion [40,68,142].

The importance of such biodegradation processes is paramount. Firstly, they may lower the structural integrity of an implant. Secondly, they may lead to periprosthetic bone loss. In this regard, one example is osteolysis resulting from generation of small polyethylene particles during wear of artificial joints. Another example is focal osteolysis, periosteal reaction and cortical thickening due to fretting corrosion of modular femoral intramedullary nails made of 316L stainless steel [260]. Thirdly, metal ions that are released as degradation products are transported by body fluids to remote tissues where they may elicit an adverse biological reaction (such as cytotoxicity, allergy or even cancer).

Different failure analysis protocols for medical devices are practiced in different laboratories and companies. Some of these are adapted from other fields, for example, aircraft [261]. Often, finite element analysis is used [262]. Implant retrieval procedures have also been documented [263]. The U.S. FDA distinguishes between mandatory and voluntary medical device failure reporting [264,265].

Mudali et al. [37] conducted a survey of 50 failures of stainless steel orthopedic implants that had been retrieved from patients. Those implants were arranged based on the reported reasons for removal, type of device, anatomical site, implant lifetime and number of components in the device. Ten cases were selected for thorough failure analysis in order to determine the mechanism and cause of failure. Fatigue-related failures were identified in three cases. Several cases were related to conjoint action of two failure mechanisms (e.g., fatigue and intergranular corrosion attack in a total knee prosthesis, fatigue and pitting corrosion in a compression bone plate and screws fixation device, and a pit-induced SCC in an intramedullary nail). In one case, of a Sherman bone plate, the failure was related to the combined action of pitting corrosion, crevice corrosion and CF.

**Figure 19:**
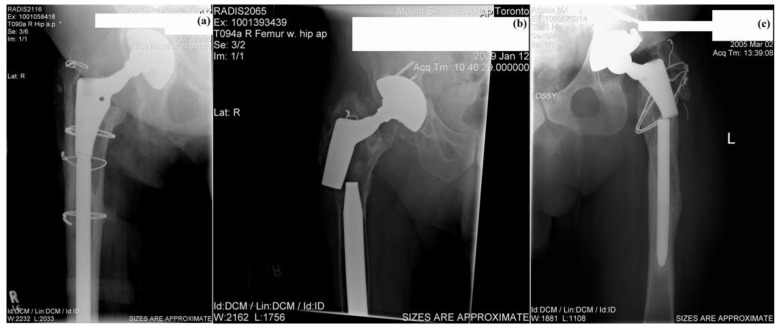
Radiographs revealing the fractures in vivo of cementless femoral stems in three humans (**a**–**c**) represent cases 1, 2 and 6, respectively, in Table I in Ref. [266]). The failures were initiated by a fretting fatigue mechanism and propagated via pure bending fatigue [266]. Reprinted with permission from Wolters Kluwer Health, Inc.

Mechanical failure of femoral stems at the modular junction of revision hip arthroplasty systems has been reported only infrequently. Lakstein et al. [266] investigated the fracture in vivo of cementless femoral stems at the mid-stem junction in modular revision hip arthroplasty systems (ZMR stems, Zimmer, Warsaw, IN). Three of the six stems studied are shown in Figure 19. It was concluded that the stem failure was initiated by a *fretting fatigue* mechanism and was propagated by a pure bending fatigue mechanism. The chemical composition and microhardness matched those of Ti–6Al–4V. Titanium and its alloys are particularly prone to fretting fatigue, especially in a corrosive environment [267]. All fracture surfaces and the adjacent circumferences were carefully inspected for etching, pitting, chloride formation, corrosion products and other possible indications of corrosion. However, no such evidence was found and thus corrosion was ruled out. Fretting fatigue occurs when contacting components are subjected to cyclic loading while small, oscillatory motion of small amplitude exists between them. Fretting increases both the tensile stress and the shear stress at the contact interface and creates defects, which might cause premature crack nucleation. Furthermore, fretting fatigue results in a strong decrease (by a factor of ≥2) in the fatigue endurance limit and orders of magnitude decrease in fatigue lifetime from that observed under pure axial cyclic loading alone [268]. The propagation of multiple cracks is a feature of fretting fatigue failures. This failure mechanism has been observed in fracture fixation devices (such as bone plates and Kuntscher nails) as well as in TJRs [267]. We shall return to fretting in the following Section, in the context of fretting corrosion.

## 7. Mechanisms of Corrosion In Vivo

**Figure 20:**
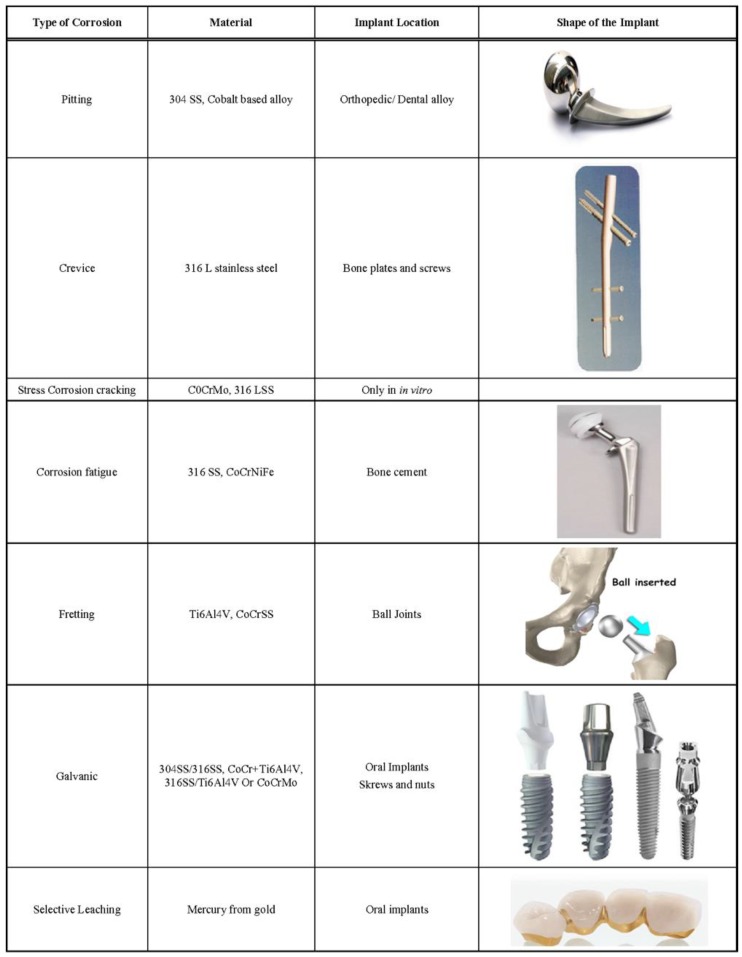
Types of corrosion in common implants [39]. Reprinted with permission from Bentham Science Publishers.

Some forms of corrosion are most typical of implants and other medical devices (see Figure 20). These include localized (both pitting and crevice), intergranular and galvanic corrosion, as well as SCC, CF and fretting corrosion [33,39,269,270,271]. The latter three are the outcome of the synergistic effect of electrochemical and mechanical factors. On the other hand, uniform (general) corrosion occurs in vivo very seldom.

### 7.1. Localized Corrosion

#### 7.1.1. Crevice Corrosion

As its name indicates, crevice corrosion occurs in narrow spaces where an electrolyte can creep, usually by capillary forces, between two metal parts or between a metal and an insulator. It is a form of localized corrosion typically related to a stagnant solution on the microenvironment level. As oxygen diffusion into the crevice is restricted, a differential aeration cell is established between the crevice (microenvironment) and the external surface (bulk environment). Oxygen depletion occurs in crevices, joints and under corrosion deposits, metallic or non-metallic deposits. Localized corrosion will take place at the area of low-oxygen concentration, which acts as an anode. Thus, crevice corrosion is often observed under riveted lap joints, bolt heads, gaskets and O-rings. The susceptibility to crevice corrosion increases with increasing hysteresis of the polarization curve, that is, the difference between *E*_b_ and *E*_p_. Therefore, a higher value of *E*_p_ reflects higher resistance to crevice corrosion. If the metal does not repassivate until a potential below the rest potential is reached, then it is very susceptible to crevice corrosion. Two concerns with this type of corrosion are that: (1) the damage is concentrated within a small hidden area, thus inspection is very difficult, and (2) the extent of damage cannot be monitored by mass loss/gain measurements [46].

The mechanism of crevice corrosion can be described as follows. The metal oxidation reaction is accompanied by oxygen reduction reaction. Initially, these reactions take place uniformly all over the surface, including in crevices; the oxygen concentration in the solution inside a crevice is equal to the level of soluble oxygen and is the same everywhere. However, after some time, oxygen in the crevice is depleted due to mass transport limitation, thus its reduction in this area ceases. Because the area of the crevice is usually very small compared to the overall surface area, we will not observe any significant drop in the overall reduction rate, thus the corrosion rate almost does not change. However, in order to compensate for the positive charge associated with metal cations being continuously formed within the crevice, anions diffuse into the crevice; these are often chloride ions. The chloride anion associates with the metal cation and the metal chloride reacts with water to form metal hydroxide and hydrochloric acid. The pH in a crevice can reach very acidic values, occasionally as those of pure acids. The acidification of the microenvironment can lead to a significant increase in the corrosion rate of most metals. The dissociated chloride ion can react again with the metal ion and the series of reactions repeat. This is an autocatalytic process. The solid corrosion products seal even further the crevice microenvironment. Because the anodic area is localized and small in comparison to the cathodic area, yet both should transfer the same current, the anode current density increases, together with the corrosion rate [46]. 

**Figure 21:**
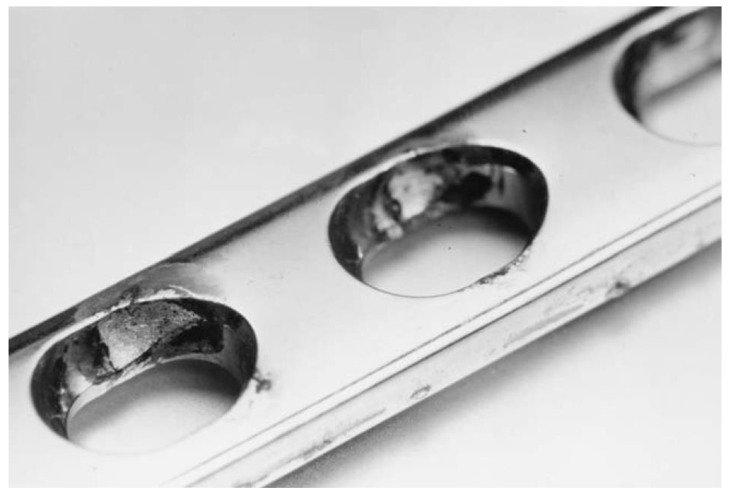
Crevice corrosion in a screw hole in fracture fixation plate [2]. Reprinted with permission from Elsevier.

Metals susceptible to pitting corrosion (see Section 7.1.2) also suffer from crevice corrosion. The presence of crevices on the surface often triggers localized corrosion already under conditions where stable pitting would not take place (e.g., at lower concentration of halides). In the case of medical devices, failures in this mechanism have been observed at the interface between bone plates and screws made of 316L stainless steel (see Figure 21), as well as in cemented Ti-based hip implants [37,38,41,271]. Because a porous matrix provides inherent crevices, it is not surprising that porous titanium [272] and porous CoCrMo alloys [273] have been shown to exhibit much higher corrosion rates compared to their dense counterparts. 

Guindy et al. [274] studied six dental implants whose late failure was related to suprastructure metal corrosion at the marginal gap. The suprastructures (crowns) were made of porcelain fused to a gold alloy that contained also Pt, Pd, Ag, Co, In and Sn. Extensive corrosion lesions and areas of oxidation were observed on all implants and inner crown surfaces. Bone tissue from around five implants showed higher contents of metal ions in comparison to physiologic baseline values. It was concluded that corrosion was initiated by the bonding oxides, which are necessary for fusing porcelain to gold, and rapidly propagated at the gap crevices. The pH in these regions is locally reduced due to both decrease of oxygen flow and bacteria colonization at the marginal gap spaces. To avoid failure recurrence, the authors suggested designing a single-unit implant-abutment as well as crowns made of a homogeneous, biocompatible Ti. In addition, all conditionally removable suprastructures should be cemented or sealed to avoid bacterial colonization and possible crevice corrosion.

#### 7.1.2. Pitting Corrosion

Pitting corrosion is a highly localized corrosion of a metal surface that is confined at a small area and takes the form of cavities. This is generally a process of local anodic dissolution, for example at local breakdowns of the passive layer, where metal loss is exacerbated by the presence of a small anode and a large cathode. Pitting is commonly observed on surfaces with little or no general corrosion. It is often initiated at MnS inclusion sites, surface scratches, exposure of dislocations or other defects to the surface, local breakdown of the passive layer due to exposure to chloride, fluoride and other halide ions or local arbitrary variations in the composition of the electrolyte solution. 

After their initiation, pits either keep growing or repassivation may occur. An alloy with a high pitting corrosion resistance should ideally combine low susceptibility to pit initiation, low pit propagation rate, and fast repassivation. For metals with electron-conducting passive films such as stainless steels, the number of pits usually correlates inversely with their average depth, since the cathodic current consumed by the large passive surface area fosters anodic dissolution inside the pits. The pits may grow at different rates, depending on the number of active pits [41].

An increase in the resistance to pitting corrosion is concomitant with an increase in *E*_b_. While pitting will occur on a pit-free surface above *E*_b_, it will occur only in the range of potentials between *E*_p_ and *E*_b_ if the surface is already pitted. Increasing the temperature usually increases the susceptibility to pitting. All metals that are susceptible to pitting corrosion are also susceptible to crevice corrosion but not vice versa. Stainless steels are susceptible in particular. The pits usually grow in the direction of gravity. They can have different shapes, such as deep and narrow, shallow and wide, elliptical, undercutting, subsurface, horizontal or vertical grain attack. ASTM G46 [275] describes the selection of procedures that can be used in the identification and examination of pits and in the evaluation of pitting, including a standard rating chart [46].

The most common mechanism of pitting corrosion of a stainless steel is as follows. In an alkaline chloride solution, anodic dissolution of stainless steel produces Fe^2+^, which attracts negative anions such as Cl^−^ to the initiation site. Hydrolysis occurs following the reaction

(29) $$Fe^{2+}+2H_{2}O+2Cl^{-}\to \mathrm{Fe}{\left(\mathrm{OH}\right)}_{2}+2\mathrm{HCl}.$$

Consequently, the pH drops in the initiation site. An autocatalytic mechanism of pit growth occurs. The acidic chloride solution rushes anodic dissolution, which consequently concentrates more Cl^−^ in the pit. Insoluble cap of the corrosion product, Fe(OH)_3_, forms at the entrance to the pit; Fe^2+^ diffuses from inside to outside of the pit, where it is oxidized to Fe^3+^ and precipitates in the neutral bulk solution. The cap inhibits escape of Fe^2+^ but is amply porous to allow Cl^−^ migration into the pit, thus preserving high acidic chloride concentration within the pit. Anodic polarization of the inner pit occurs by coupling to the outer passive cathodic surfaces. Reduction of solute oxidizer such as oxygen consumes the electrons that are generated by the anodic reaction in the pit [46].

Pitting corrosion was a common problem with the early 304 stainless steel implants. However, the addition of 2–3 wt% Mo in 316L stainless steel has greatly reduced the number of failures due to pitting corrosion [36]. Mudali et al. [276] reported that alloying annealed 316L stainless steel with 0.05–0.22 wt% nitrogen significantly increased the pitting corrosion resistance in a 0.5 M NaCl electrolyte. A synergistic effect of nitrogen alloying and cold working of up to 20% provided an improved pitting resistance. However, at higher cold working levels, the pitting resistance decreased, the effect being more pronounced at higher nitrogen contents. These synergistic effects were attributed to the role of nitrogen in increasing the density of fine deformation bands. 

Cobalt-based alloys have been found resistant to pitting corrosion under static conditions [277,278,279] but exposed to pitting corrosion under cyclic loads or following severe cold work [280]. In addition, in vivo pitting of CoCrMo is a common characteristic of corrosion damage near or within modular tapers. It indicates the likelihood that the electrode potential of this alloy can be shifted very positive compared to its rest potential in physiological solutions, probably due to exposure to reactive oxygen species (ROS) associated with inflammation [271].

Titanium is susceptible to passivity breakdown in chloride–containing solutions only at very high anodic potentials that are not met in any in vivo environment. For example, for CP-Ti, pitting potentials higher than 10 V have been documented in chloride-containing solutions. Ti is an interesting exception in that the chloride ion is the least pit-triggering anion among the halides, in contrast to other materials with a passive layer [41]. Although titanium alloys may be less resistant than CP-Ti due to discontinuities in the protective oxide film, in vivo pitting-related failures have not been reported, to the best of my knowledge.

It is worth noting, however, that metastable pitting corrosion has been reported for both CP-Ti and Ti–6Al–4V in NaCl and in SBFs far below the pitting potential, even at the OCP. This means that local passivity breakdown events can occur in the passive region of titanium but instead of pit propagation, the small pits do not remain active; instead, fast repassivation occurs. Such events of metastable pitting can be witnessed experimentally as current transient bursts within the passive region in potentiodynamic or potentiostatic polarization curves, as long as the experiments are conducted with sufficient current and time resolutions and no noise filtering is imposed. For Ti in chloride-containing solutions, these events can typically only be resolved using microelectrodes. Although metastable pitting does not result in a complete deterioration of the system, it nevertheless indicates that passivity of the metal is not fully stable in the environment and the small activation/repassivation events contribute to passive dissolution modes. Furthermore, in contrast to the metastable pitting behaviour of stainless steels, where the number of pit initiation events typically drops to virtually zero over time, for titanium the metastable pitting activity remains high throughout the experiment (namely, for some hours). This behaviour may be non-desirable with respect to metal ion release from Ti-based implants [41].

The risk of pitting corrosion in the oral cavity is much higher due to the availability of oxygen and acidic foodstuffs. However, the development of ultraclean grades, such as 316LVM, and/or nitrogen additions have reduced this risk for stainless steels. On the other hand, in vitro experiments have shown that titanium alloys might suffer from pitting at high potentials in saline or in the high fluoride solutions used in dental cleaning procedures [36]. CP-Ti exposed to various static immersion tests has also shown a significant increase in ion release (by approximately four orders of magnitude) in the presence of fluoride [281].

**Figure 22:**
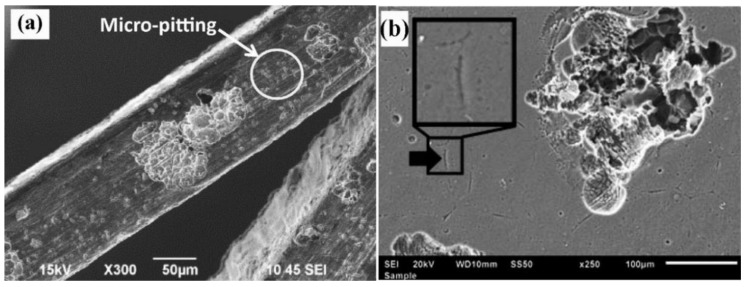
Pitting corrosion. (**a**) An oxidized tubing Nitinol stent after six months implantation into the iliac artery of a minipig [283]. (**b**) A Mg–Y–Ca–Zr WX11 alloy in the as-cast condition following potentiodynamic polarization test in DMEM with 10% FBS at 37 °C and cleaning with CrO_3_/AgNO_3_ solution [284]. (b) also shows corrosion at prone grain boundary regions (arrow). Reprinted with permission from Elsevier.

ASTM F746 [282] describes a laboratory screening test for the determination of the resistance of passive metals and alloys from which surgical implants are manufactured to pitting or crevice corrosion or both. Figure 22 shows two examples of pitting corrosion in vivo. Figure 22a shows micro-pitting on an oxidized tubing (OT) Nitinol stent after six months implantation into the iliac artery of a minipig [283]. Figure 22b shows the surface of a Mg–Y–Ca–Zr WX11 alloy in the as-cast condition after potentiodynamic polarization test in Dulbecco’s Modified Eagle Medium (DMEM) with 10% FBS at 37 °C and cleaning with CrO_3_/AgNO_3_ solution to remove the corrosion products. Pitting corrosion, a commonly observed phenomenon in Mg alloys, is evident along with corrosion at grain boundary regions (arrow) that are prone to corrosion due to higher secondary phase localization [284].

### 7.2. Intergranular Corrosion

**Figure 23:**
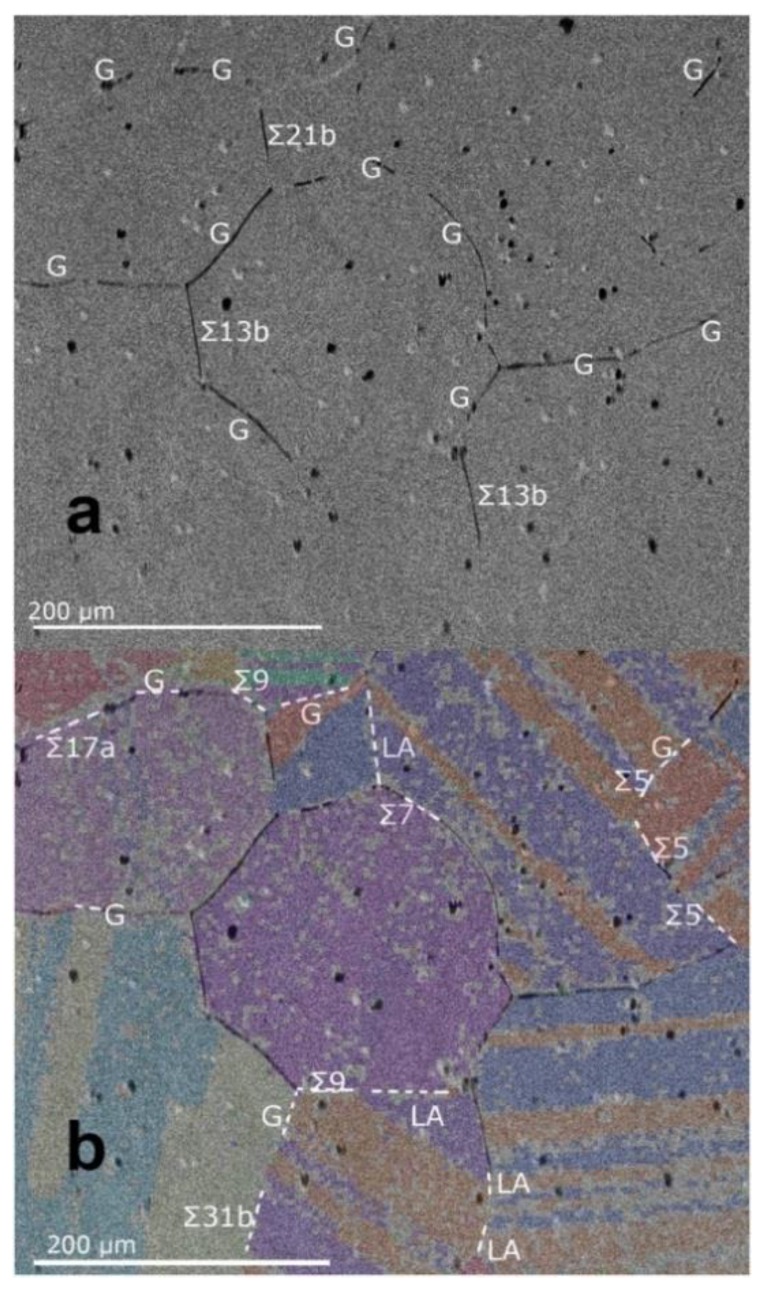
(**a**) Indexing of corroded grain boundaries based on orientational image mappings (OIM) established by electron backscatter diffraction (EBSD). (**b**) Indexing of uncorroded grain boundaries based on OIM (overlaid). “G” indicates general grain boundary geometry with no lattice site coincidence, “LA” indicates low angle [287]. Reprinted with permission from John Wiley & Sons, Inc.

The term intergranular corrosion refers to corrosion occurring at grain boundaries due to the presence of a second phase, contaminants or atom segregation. Galvanic cell is then established, with the anode around/at grain boundaries. Two examples of intergranular corrosion in engineering materials are sensitization and exfoliation [46]. Since the carbon content in 316 stainless steel was lowered in the 316L and 316LVM grades, sensitization of this steel is no longer a problem as it used to be. Intergranular corrosion has been observed primarily in CoCrMo alloy in modular taper junctions [271,285,286] but also in some cases of Ti–6Al–4V [271]. Figure 23 shows intergranular pitting corrosion of a CoCrMo alloy [287]. Electrochemical corrosion tests were conducted in a solution of bovine calf serum (BCS) with protein, which was buffered at pH 7.4 and kept at 37 °C. The attack was localized at phase boundaries and grain boundaries of high energy (namely, high-angle and low lattice coincidence, Σ11 or higher). In contrast, grain boundaries of lower energy did not seem to corrode.

### 7.3. Dealloying

Dealloying, also referred to as *selective leaching* or parting corrosion, is a corrosion process in which the more active metal is selectively removed from an alloy, leaving behind a porous weak deposit of the more noble metal. Specific categories of dealloying often carry the name of the dissolved element. For example, the preferential leaching of zinc from brass is called dezincification. In the dealloying process, typically one of two mechanisms occurs: (1) alloy dissolution and replating of the cathodic element, or (2) selective dissolution of an anodic alloy constituent. In either case, the metal is left spongy and porous and loses much of its strength, hardness and ductility [46].

**Figure 24:**
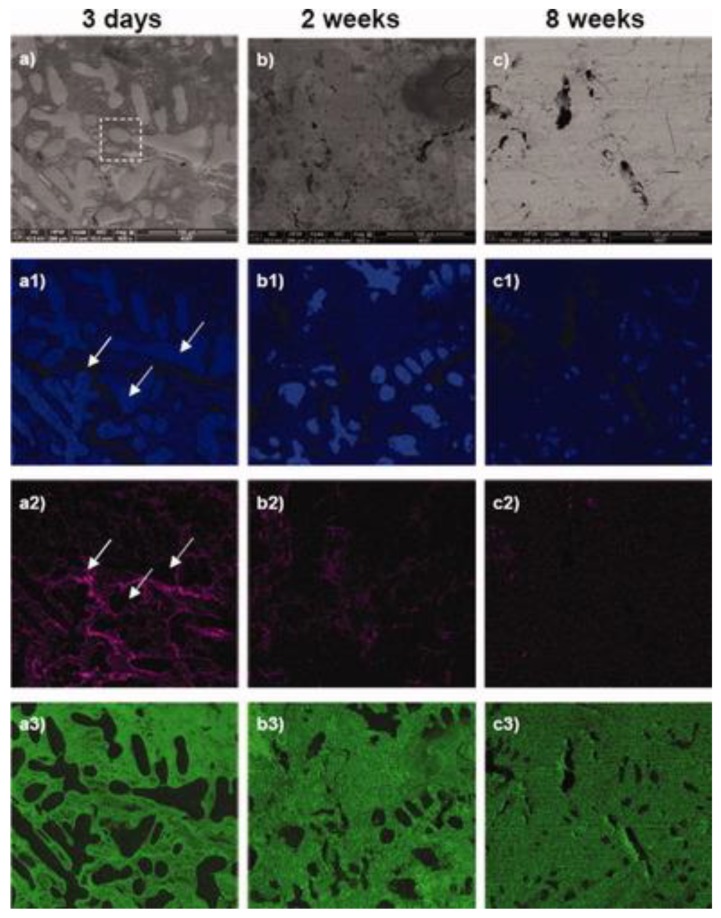
Comparison of elemental mapping of the cross-sections of cylindrical Mg–Ca specimens, which were implanted in the subchondral bone of rat femoral condyle, at (**a**) 3 days, (**b**) 2 weeks, and (**c**) 8 weeks post-implantation. (**a–c**) Cross-sectional SEM images acquired by a BSE detector. EDS elemental maps show the distributions of Mg (**a1–c1**), Ca (**a2–c2**) and O (**a3–c3**). The white dotted arrows direct to Mg-depletion traces (a1) and Ca-rich lines (a2) between primary Mg particles and the lamellar structures. The in vivo corrosion is macroscopically governed by the interdiffusion of Ca and O along the three-dimensional lamellar network and by the simultaneous surface diffusion of the primary Mg phase [292]. Reprinted with permission from John Wiley & Sons, Inc.

In some specific binary alloys containing Ca and Zn, decalcification or dezincification occur because of the significant difference in electronegativity of the two elements [288,289,290,291,292]. A similar phenomenon has been observed in Mg–Ca alloys. Jung et al. [292] elucidated the in vivo corrosion mechanism of biodegradable Mg–10Ca (wt%) in rat femoral condyle. It was found that the lamellar Mg_2_Ca phase was quickly corroded by interdiffusion of Ca and O, leading to a structural change from lamellar network to nanocrystalline MgO (see Figure 24). In contrast to the high corrosion rate of the lamellar structure, the primary Mg phase slowly transformed into nanocrystalline MgO through surface corrosion by oxygen delivered along the lamellar networks. The rapid interdiffusion induced an inhomogeneous Ca distribution and led to the formation of a transient CaO phase, which acted as a selective leaching path for Ca. In addition, the outgoing Ca with P from body fluids formed needle-type CaP at the interior and surface of the implant.

Selective leaching is usually not so important, even in dentistry. Tai et al. [293] studied leaching of nickel, chromium and beryllium ions from crowns made of Rexillium III base Ni–Cr alloy, which contains approximately 75% Ni, 13% Cr and less than 1.8% Be, in an artificial oral environment. That simulated 1-year period of mastication showed that nickel and beryllium were released both by dissolution and occlusal wear.

### 7.4. Galvanic Corrosion

Galvanic corrosion, also known as bimetallic corrosion, is an accelerated corrosion of a relatively active metal (anode) when it is brought in electrical contact with a more noble metal (cathode) in a common electrolyte. This form of corrosion may be either uniform or localized. It can be particularly severe under conditions where protective corrosion films do not form or where they are removed by conditions of erosion corrosion. ASTM G71 [294], ASTM G82 [295] and ASTM F3044 [296] are three applicable standards.

The *EMF series* is generated by arranging the standard potentials of different half-cell reactions at standard conditions ([M^n+^] = 1 M, *p* = 1 atm, *T* = 25 °C) relative to the SHE. According to the International Union of Pure and Applied Chemistry (IUPAC), this series is expressed in the form of reduction potentials, from positive values (noble metals) at the top to negative values (active metals) at the bottom. The potential represents the tendency of the reaction to occur; it is an intensive thermodynamic property. *E*^0^ of the cell must be positive in order that the overall cell reaction will occur spontaneously in the direction written [46].

Metals such as Au and Pt are very noble, that is, they have low driving force for oxidation in aqueous solutions; hence, they tend to maintain their metallic form in vivo. Other metals at the bottom of the EMF series, including titanium, have high driving force for oxidation. Fortunately, the practical nobility, rather than thermodynamic nobility, is what matters in service. From practical standpoint, the nobility is higher as the immunity and passivation regions in the Pourbaix diagrams extend below and above the water stability region and as these regions overlap with the pH range of 4–10. As discussed before, titanium and its alloys serve very well in vivo. This is because they become passive (i.e., essentially inert) under most service conditions due to the spontaneous, rapid formation of a dense, fully covering and well adhered oxide layer that serves as a *kinetic* barrier to the transport of metal ions and electrons. Other alloys that rely on the formation of a passive film to prevent oxidation are based on iron, zirconium, cobalt, tantalum, niobium and so forth.

Dissimilarity of electrodes may result also from a non-uniform chemical composition of the electrode material, local changes in solution chemistry or dissolved oxygen concentration, different processing routes (e.g., wrought versus cast Co alloys) and surface defects. 

The EMF series though has several limitations: (1) it reflects standard conditions, while in situations of practical interest the system is rarely, if ever, under standard conditions; (2) it lists only pure metals, not alloys; (3) it does not reflect kinetic effects such as passivity [46].

**Figure 25:**
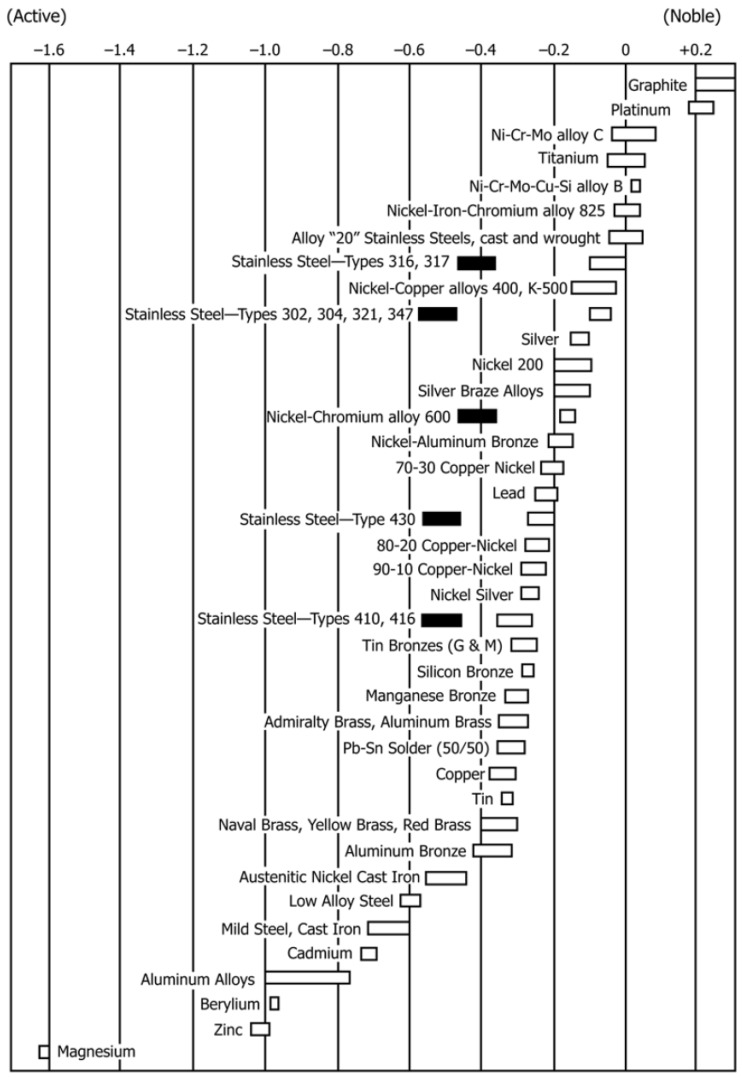
A galvanic series of various metals and alloys in flowing seawater at 2.4 to 4.0 m s^–1^ for 5 to 15 days at 5 to 30 °C. Dark boxes indicate active behaviour of active-passive alloys. Values of top axis: volts versus SCE. Source: ASTM G82–98(2014) [295].

The *Galvanic series*, Figure 25, is more appropriate than the EMF series for description of actual corrosion problems as alloys and not pure metals are often coupled, and the metals are undergoing corrosion rather than being in equilibrium with their ions at standard concentrations. Usually, the further apart two metals are in the series (i.e., the greater the potential difference between them), the greater is the driving force for galvanic corrosion and the greater the damage to the anode will often be [46]. Often, the maximum allowable potential difference between the metals is 200 mV.

The anode-to-cathode surface areas ratio is also important: the lower this ratio is, the higher current density on the anode is and the higher corrosion rate is. The maximal damage will occur near the plane of contact between the two metals, because the driving force farther along is diminished by the potential drop across the solution resistance. Low conductivity of the electrolyte solution and low polarization usually focus the galvanic attack closer to the contact zone. The nature of the environment is also important [46].

A common current meter (ammeter) measures the current as a voltage drop across an internal shunt resistor. When measuring the current in common electric circuits, this voltage drop can be disregarded. However, it is too high to allow accurate measurement of small currents in galvanic corrosion or local oxide breakdown. An ammeter for such electrochemical measurements should (ideally) have an internal zero resistance. Zero-resistance ammeter (ZRA) is a current-to-voltage converter that produces a voltage output proportional to the current flowing between its input terminals while imposing a “zero” voltage drop (zero resistance) to the external circuit. Any potentiostat can be used as a ZRA: simply connect electrode WE1 to the electrode terminal, WE2 to both the RE plug and the CE plug, set the potential to 0 mV and switch to potentiostatic mode (counter electrode on). In this setup, a current is displayed on the potentiostat’s current meter and a corresponding voltage is fed to the current output terminal. The voltage between WE1 and WE2 is zero! Thus, you measure the current between two electrodes that are kept at the same potential [46]. 

In zero-resistance ammetry (see ASTMs G71 and G82), the galvanic current *I*_couple_ between two dissimilar electrodes or between two presumably identical electrodes exposed to different environments, is measured by a ZRA. The dissimilar electrodes may represent different chemical compositions, heat treatments, surface conditions, residual stress levels and so forth. This technique has been found useful to study depolarization of the cathode in a galvanic couple, detecting either low levels of dissolved oxygen or the presence of bacteria that increase the coupling current [46].

In principal, strategies for prevention of galvanic corrosion include selection of materials with as similar electrode potentials as possible, use of insulators between dissimilar metals, and use of coatings or special designs to limit the cathode area relative to the anode area. 

Contact between dissimilar metals immersed in an electrolyte is common in orthopedic, dental and other biomedical applications. Examples include hip prostheses with ball made of 316L stainless steel and socket made of Ti–6Al–4V, a CoCrMo femoral head in contact with a Ti–6Al–4V femoral stem, and a gold crown coupled to an amalgam core in the oral cavity. 

When titanium- and cobalt-based alloys are coupled together in vivo, it may be anticipated that the passive titanium alloy would become the cathode while the less passive cobalt alloy would undergo accelerated corrosion. In practice, however, since the kinetics of the oxygen and water reduction reactions are slow on titanium surfaces and because the passive current of titanium is virtually independent of potential so it is easily polarized, titanium is a poor cathode. This means that the extent of accelerated corrosion caused to any metal from coupling to titanium should be small. Thus, titanium-cobalt combinations have been found stable both in vitro and in vivo, at least as long as no relative motion (fretting) occurs [297,298,299]. On the other hand, 316L stainless steel is susceptible to pitting corrosion when it is coupled to either Ti- or Co-based alloys [300]. Mellado-Valero et al. [301] analysed the galvanic corrosion of CoCr, CoCr-c, NiCrTi, Au-Pd and Ti–6Al–4V dental alloys for implant superstructures when coupled to Ti grade 2 implants in artificial saliva (AS), with and without fluorides in different acidic conditions. It was concluded that NiCrTi is not recommended for implant superstructures due to the risk of Ni ion release to the body and that fluorides should be avoided in acidic media because Ti, Ti–6Al–4V and CoCr-c superstructures show galvanic corrosion. The best combinations were Ti/Ti–6Al–4V and Ti/CoCr, as alternative to noble gold alloys. Arslan et al. [302] compared between the galvanic couples Ti–6Al–4V/Au, Ti–6Al–4V/CoCr and Ti–6Al–4V/CrNi in Ringer’s solution by the use of Tafel plots, Evans diagrams and EIS Nyquist plots. It was concluded that the Ti–6Al–4V/Au couple was most suitable for dental applications, whereas the corrosion behaviours of Ti–6Al–4V/CoCr and Ti–6Al–4V/CrNi couples were similar. Zitter and Pauluzzi [303] reported galvanic corrosion of an implant consisting of almost equal parts of iron and brass, where brass was cathodically protected until the iron part was completely dissolved. British/ISO standard 21534 [304] defines acceptable and non-acceptable combinations of materials for either articulating or non-articulating contacting surfaces of implants. 

### 7.5. Stress Corrosion Cracking (SCC)

SCC is a phenomenon associated with the combination of static tensile stress and corrosive environment. Failures often occur in mild environments under tensile stresses well below the macroscopic yield strength of the material. The origin of tensile stresses may be external forces, thermal stresses or residual stresses. In general, as these stresses increase, the time required to initiate SCC decreases. The kinetics of SCC also depends on the chemical and metallurgical state of the material (e.g., chemical composition, thermal condition, grain size, presence of secondary phases and precipitates); on environmental conditions (e.g., environment composition, temperature, pressure, pH, electrochemical potential, solution viscosity and mixing); and on crack geometry and stress state (uniaxial, triaxial and so forth.) [46].

SCC may be intergranular (IG), transgranular (TG) or mixed, depending on the alloy, its microstructure and the environment. However, the crack follows a general macroscopic path that is always normal to the tensile component of stress. TG failures are less common than the IG ones but both may exist in the same system or even in the same failed part, depending on conditions. The IG failure mode suggests some inhomogeneity at the grain boundaries. For example, segregation of sulphur and phosphorous at grain boundaries has been suggested as the cause of IG SCC of low-alloy steels [305].

The resistance to SCC depends, among others, on the thermal condition of the alloys as well as on the crystallographic direction in the material. On the anodic polarization curve, two zones of the highest likelihood of TG-SCC are around the bottom and upper parts of the passive region. IG-SCC may occur within a broader potential range, due to inhomogeneities at the grain boundaries. Despite large amount of data, the mechanisms of SCC are still under considerable debate. Several mechanistic models have been suggested for crack propagation. These mechanisms are divided into anodic and cathodic SCC. Cross-sections of SCC often reveal branched cracks. This river branching pattern is unique to SCC and is used in failure analysis to identify this corrosion form [46,305].

Blackwood has claimed [36] that SCC had not been observed on recovered surgical implants. Kannan and Raman [306] evaluated the SCC susceptibility of sand-cast Mg–Al–Zn alloy in modified-SBF using the slow strain rate test (SSRT) method. It was concluded that the SCC susceptibility of this alloy is not substantial should not be of concern in clinical applications. Jafari et al. [307] reviewed the SCC and CF of Mg alloys in chloride-containing corrosive environments, including SBFs and the associated fracture mechanisms, as well as the critical relevance to biodegradable implant applications. Bundy et al. [308] studied 316L stainless steel, a Co–Cr alloy, Ti–6Al–4V ELI and MP35N implant alloys exposed simultaneously to tensile stresses and corrosion environments. In the in vivo studies, the stainless steel and the titanium alloy exhibited cracklike features when loaded to the yield stress and implanted for 16 weeks. The Co–Cr alloy stressed beyond *σ*_y_ exhibited cracklike features in plastically deformed areas, while MP35N appeared to be immune. The in vivo environment was found to be more aggressive than a 37 °C Ringer’s solution due to both bioelectric effects and organic constituents. Bombara and Cavallini [309] investigated fixation nails made of 316 stainless steel that failed during service in the recovery treatment of femoral fractures. It was concluded that pitting in crevice-induced SCC from acid chlorides was the failure mechanism. The authors thus suggested using the new high-Cr ferritic stainless steels that are extra low in interstitial elements, which are much more resistant than ordinary austenitic grades to pitting and SCC in chloride media. Jones et al. [310] reported that cast Vitallium and 316L stainless steel are not susceptible to SCC in a physiological saline (Tyrode’s) solution. Several high-strength, high-ductility stainless steels (TRIP steels) also appeared to be immune to SCC. SCC has also been observed in several cases of orthopedic implants made of stainless steels [37,311]. Industrial uses of stainless steels in saline environments also support possible susceptibility to SCC [2]. Wang et al. [312] analysed the fracture of the CoCrMo connection taper in the Revitan™ revision modular femoral stem. It was concluded that the failure started with crack nucleation by SCC and propagated by corrosion fatigue. The SCC in the connection taper was characterized by crevice pitting-associated multiple cracks on the lateral side of the implants subjected to dynamic tensile stress. Some of these cracks coalesced and propagated to a critical size under dynamic tensile loading, leading later to corrosion fatigue fracture.

**Figure 26:**
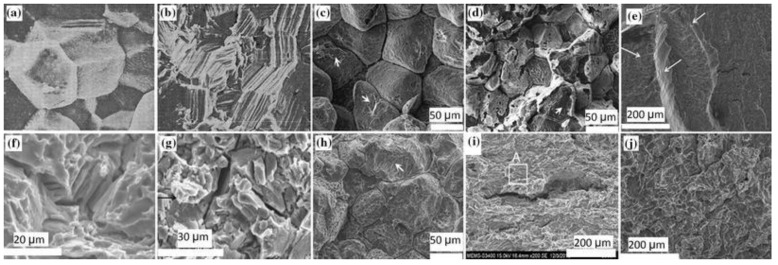
SEM fractographs of various Mg biomaterials that fractured by SCC in different solutions. (**a,b**) represent crack propagation by an anodic dissolution mechanism in in NaCl + K_2_CrO_4_ solution; the rates of film rupture and regrowth compete. (**a**) IGSCC, (**b**) TGSCC of Mg–Al alloy. (c) through (h) represent crack propagation by a mixed-mode mechanism. (**c**) IGSCC of ZE41 in NaCl. The IGSCC is associated with second-phase particles along grain boundaries. (**d**) TGSCC of QE22 in NaCl. The TGSCC fracture path is consistent with a mechanism involving hydrogen. (**e,f**) TGSCC of ZX50 and WZ21 in modified simulated body fluid (m-SBF), respectively. The electrochemical breakdown or mechanical rupture of the protective film resulted in entry of hydrogen into the alloy matrix and subsequent embrittlement. (**g**) IGSCC of WE43 in m-SBF. The intergranular cracking (IGC) is associated with electrochemical dissolution along the grain boundaries. (**h**) IGSCC and TGSCC of EV31A in NaCl. While the IGC is due to the presence of bulky precipitates at grain boundaries, the transgranular cracking (TGC) is due to hydrogen entry into the matrix. (**I**,**j**) represent hydrogen-assisted cracking mechanism. (**i**) TGSCC of Mg–Mn in NaCl. TGSCC with the evidence of flat parallel facets is an indication of the hydrogen-assisted cracking. (**j**) TGSCC of AZ91D in m-SBF. Fracture of protective films at the crack surface allows hydrogen to diffuse into the alloy matrix [307]. Reprinted with permission from Springer Nature.

Figure 26 shows typical fractographs of different Mg alloys that failed in different solutions due to SCC. SCC crack propagation is often attributed to either continuous crack propagation by anodic dissolution at the crack tip or discontinuous crack propagation by hydrogen-assisted processes. In the former case, mechanical stress can cause localized rupture of the protective film ahead of the crack tip. Next, dissolution at film-free crack tip causes crack extension and further crack propagation, which is defined by the competing processes of film rupture and repassivation. In the second case, atomic hydrogen generated during the cathodic reaction of Mg alloys (and even at anodic potentials due to the negative difference effect) can enter the Mg matrix and cause cracking [307].

### 7.6. Corrosion Fatigue (CF)

The term corrosion fatigue refers to the initiation and propagation of cracks as a result of the synergistic effect of a cyclic stress and a corrosive environment. An aggressive environment usually has a harmful effect on fatigue strength and fatigue lifetime, producing failure in fewer stress cycles than would be required in a more inert environment. The action of the cyclic stress leads to local destruction of surface oxides, hence, corrosion pits form. The cyclic stress also removes corrosion products that usually inhibit further corrosion. The bottom of the pit is anodic compared to the remaining surface, therefore corrosion propagates inward, being helped by destruction of oxide layers by cyclic strain. Cracking will appear when the pit becomes sufficiently sharp to establish high stress concentration [46,313].

Corrosion fatigue depends strongly on the interactions between mechanical, metallurgical and environmental variables. Mechanical variables include the maximum stress or stress-intensity factor, stress amplitude, stress ratio, cyclic loading frequency, state of stress, residual stress, crack size and shape and their relation to component size and geometry and so forth. Metallurgical variables include alloy composition, distribution of alloying elements and impurities, microstructure and crystal structure, heat treatment, mechanical working, preferred orientation of grains and grain boundaries (texture), mechanical properties (e.g., strength or fracture toughness) and so forth. Environmental variables include temperature, pH, types of environment (e.g., gaseous or liquid), partial pressure of damaging species in gaseous environments, concentration of damaging species in aqueous or other liquid environments, electrical potential, viscosity of the environment, coatings, inhibitors and so forth. [46].

Corrosion fatigue usually leads to TG fracture with little or no branching. Individual cracks may occur, although families of parallel cracks are more common. Decreasing the strain rate increases the effect of environment. Corrosion fatigue is more pronounced at low stress frequencies. Often, endurance limit is not evident in aggressive environment. All metals and alloys are susceptible to CF. Even some alloys that are immune to SCC, for example, ferritic stainless steels, are subject to failure by CF [46]. ASTM F1801 [314] describes the CF testing of metallic implant materials. ISO 14801 [315] specifies a method of dynamic testing of single post endosseous dental implants of the transmucosal type in combination with their premanufactured prosthetic components. While it simulates the functional loading of an endosseous dental implant under “worst case” conditions, it is not applicable for predicting the in vivo performance of an endosseous dental implant or dental prosthesis, particularly if multiple endosseous dental implants are used for a dental prosthesis.

Many medical devices are subjected to low-frequency loads, for example, normal walking results in a hip implant being subjected to cyclic loads at about 1 Hz. Yet, it has been claimed that CF holds only a minor percentage of total fatigue failures of implants [36]. It was also claimed that CF occurs mainly when specifications of materials and processes are not followed or after long implantation periods [54,316]. 

Morita et al. [56] reported that the in vivo fatigue strengths of 316 stainless steel and CoCrNiFe alloy were considerably lower than the equivalent in vitro values. These authors suggested that this was due to the low dissolved oxygen concentration in body fluids. Piehler et al. [317] tested hip nail plates and found that large plates had better CF resistance than small ones and that Ti–6Al–4V performed better than 316L stainless steel. Hughes et al. [61] found that the CF resistance of titanium was almost independent of pH over the range of 2 to 7, whereas the fatigue strength of stainless steel declined rapidly below pH 4. This is consistent with the finding [318] that pitting corrosion facilitates the initiation of CF in stainless steels. In the latter work, it was also reported that the CF resistance of Ti–6Al–4V could be enhanced by nitrogen implantation and heat treatments to produce fine grain sizes [36]. Okazaki et al. [319] compared the corrosion resistance and the CF strength of Ti–15Zr–4Nb–4Ta–0.2Pd–0.2O–0.05N and Ti–15Sn–4Nb–2Ta–0.2Pd–0.2O alloys with those of Ti–6Al–4 V ELI, Ti–6Al–2Nb–1Ta, CP-Ti grade 2 and *β* type Ti–15Mo–5Zr–3Al. The fatigue strength at 10^8^ cycles of the first two alloys was lower than that of Ti–6Al–2Nb–1Ta. The fatigue strength of the *β*- type Ti–15Mo–5Zr–3Al alloy at 10^7^ cycles was lower than that of the *α*+*β* type alloys. Fleck and Eifler [320] reviewed the corrosion, fatigue and corrosion fatigue behaviour of titanium and its alloys, with a special emphasis on the influence of simulated in vivo conditions. Papakyriacou et al. [321] compared the corrosion fatigue properties of cold worked CP-Nb and CP-Ta with those of CP-Ti and Ti–6Al–7Nb. Constant amplitude fatigue experiments (S–N curves) were performed at ultrasonic frequency (20 kHz) with two different surface finishes–ground and blasted and shot peened. Two environments were studied–ambient air and a corrosive fluid similar to the body fluid in the oral cavity. The endurance limit at 2 × 10^8^ cycles of all materials decreased by 5–20% in corrosive fluid compared to ambient air. The loss of fatigue strength was more pronounced for ground Ti–6Al–7Nb and CP-Ti than for ground CP-Nb and CP-Ta. Fractography showed a more significant embrittlement of ground Ti–6Al–7Nb alloy and CP-Ti after cycling in corrosive fluid than ground CP-Ta and CP-Nb. A positive effect on the fatigue properties of surface modification by blasting and shot peening was found for all materials in both environments. It should be noted that the values obtained at such a high frequency might not be fully representative of the actual in vivo behaviour; yet, for comparative screening they may be good enough.

Raman and Harandi [322] reviewed the studies of CF of Mg alloys in pseudo-physiological solutions and the employed testing procedures. Mg alloys are reported to be susceptible to CF also in the corrosive human body fluids. The CF of Mg alloys, both in vitro [323,324] and in vivo [307], is also described elsewhere.

Diener and Speidel [325] compared the high-cycle CF data of nitrogen-bearing austenitic stainless steels with similar data for Ti- and Co-base alloys. The beneficial effect of alloying with nitrogen was demonstrated. Colangelo [326] examined orthopedic devices made of type 316 SMO stainless steel after retrieval from humans. In addition, bending fatigue tests were conducted in air and in isotonic saline solution in order to determine the crack growth rates. Data followed the empirical Paris law, with *m* rising from 3.23 in air to 4.16 in isotonic saline. A clear change in the appearance of the fracture surface was noticed on the specimens fractured in the saline environment. 

**Figure 27:**
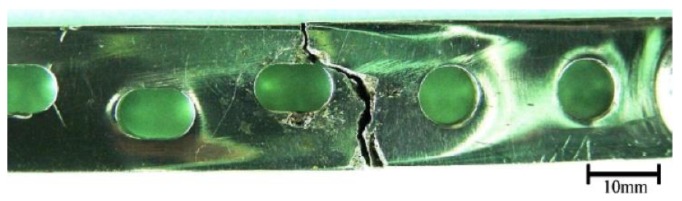
Top view of the fitted fractured mates of a stainless steel plate after ultrasonic cleaning [327]. Reprinted with permission from Elsevier.

Amel-Farzad et al. [327] examined a stainless steel orthopedic DCS barrel (femoral) plate, which had fractured inside a patient’s thigh (Figure 27). The plate had apparently fractured during the first few months in vivo, when the bone had not been reconstructed completely. Different damages were observed, such as pits related to crevice corrosion, initiation of cracks from these pits, intergranular surface cracking inside the crevice and also SCC-like branched cracks. Yet, the main failure mechanism was determined to be CF assisted by crevice corrosion. The material used to manufacture the plate had a chemical composition that deviated significantly from the requirements of ASTM F-138 standard for 316L stainless steel. The low quality of the material led to the onset of pitting corrosion inside the crevices between the screws and the plate. Next, cyclic loading nucleated cracks at the localized corrosion sites. Cracks propagated with the increase in stress cycles, and when a critical size was attained–the catastrophic fracture happened.

Hamandi et al. [328] conducted failure analysis of a fractured stainless steel proximal humerus internal locking system (PHILOS) plate and screws, used for ankle arthrodesis. It was found that the plate failed by CF, whereas overload separated the screws in two parts. Three-dimensional models of the plate and the screws (cortical, locking and cannulated) were prepared using Solidworks and imported into ANSYS to simulate the loading conditions and regions of stress development. Statistical analysis was done to understand the effect of load, screw design pattern, coefficient of friction between the plate and screws and cortical screw displacement on the maximum von Mises stresses of the locking compression plate. The finite element simulation of the plate validated the fractographic results. Fractographic examination of the cortical and locking screws supported the mechanism of CF fracture propagating from pit initiation sites associated with inclusions.

**Figure 28:**
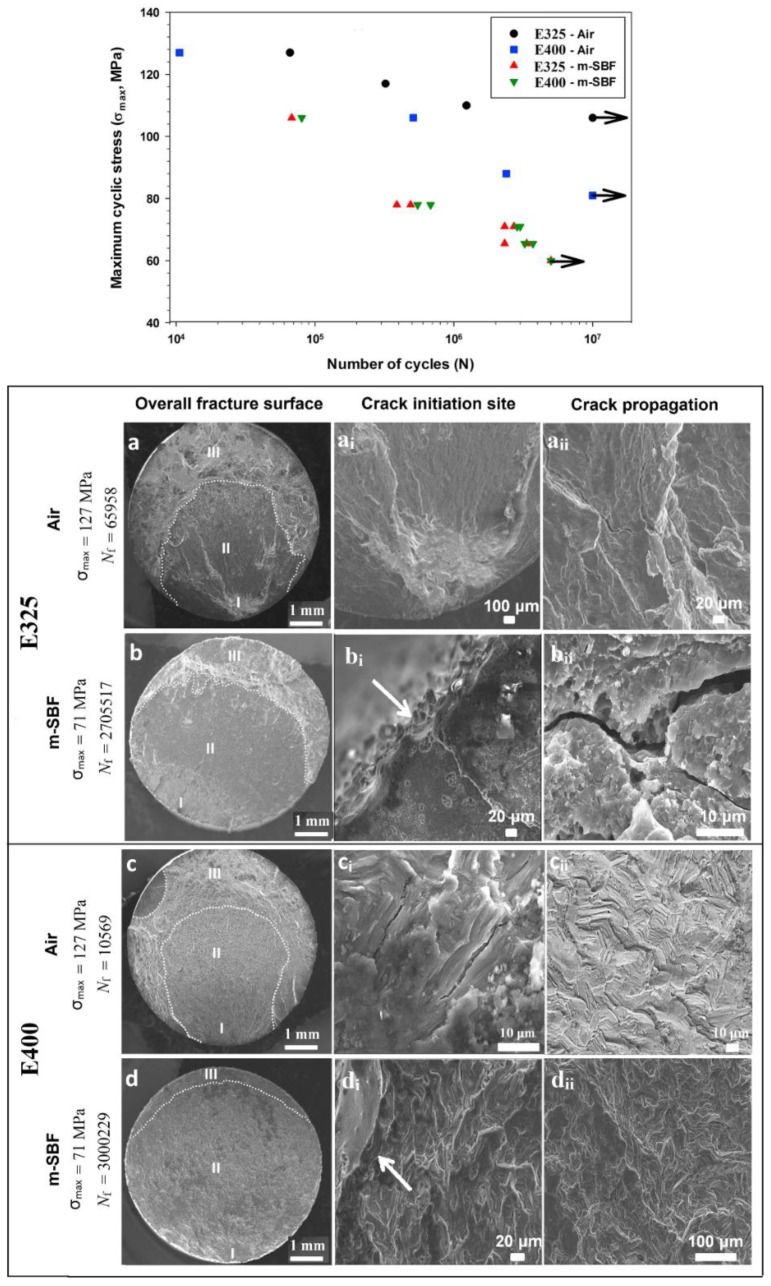
Top: S-N fatigue curves for E325 and E400 Mg alloys tested in air and m-SBF. Arrows correspond to run-out samples. Bottom: SEM fractographs: (**a**) E325 tested in air, (**b**) E325 tested in m-SBF, (**c**) E400 tested in air, (**d**) E400 tested in m-SBF. *σ*_max_ is the maximum applied stress amplitude and *N*_f_ is the number of cycles to failure. The arrows in b_i_ and d_i_ indicate locations of crack nucleation [324]. Reprinted with permission from Elsevier.

Figure 28 [324] shows the S-N curves and the typical fractographs of a newly developed high-strength low-alloy Mg alloy, MgZn_1_Ca_0.3_ (ZX10), processed at two different extrusion temperatures of 325 and 400 °C (named E325 and E400, respectively) and tested both in air and in m-SBF. While E325 has superior fatigue properties to E400 when tested in air due to a grain-boundary strengthening mechanism (106 and 81 MPa, respectively, at 10^7^ cycles), both show similar fatigue strength of 60 MPa at 5 × 10^6^ cycles when tested in m-SBF. Fractographs of the samples tested in m-SBF revealed that corrosion pits served as crack nucleation sites. Whereas the fracture of E325 was transgranular, E400 displayed a mixed-mode cracking, namely both transgranular and intergranular [324].

### 7.7. Fretting Corrosion

*Tribocorrosion* is defined as wear-accelerated corrosion of a material surface [329]. A special mode of tribocorrosion very relevant to implants is *fretting corrosion*, which is of particular risk to spinal fixation systems, stem/neck and neck/head interfaces in modular hip implants, screw/plate junctions of fracture fixation plates and dental implants. Nevertheless, fretting corrosion may occur also at backside contacts in joint prostheses, bone-implant contact regions, overlapping cardiovascular stent contact points and other locations where surface contact and small cyclic motion exist [40]. Hence, fretting corrosion of biomaterials and implants has been studied extensively in the lab as well as on retrieved implants [40,68,142,143,271,329,330,331,332,333,334,335,336,337,338,339,340,341,342,343,344,345,346,347,348]. ASTM F1875 [349] specifies fretting corrosion testing of hip femoral head/bore and cone taper interface in modular implants.

Fretting fatigue takes place when two mating surfaces are subjected to cyclic loading while small, oscillatory displacements (on the order of microns) with a total amplitude smaller than the contact width occur between them. This phenomenon was already discussed in Section 6. When corrosion is simultaneously involved, the phenomenon becomes fretting corrosion.

The damage in fretting corrosion is mainly limited to the local site where generated debris are accumulated, leading to an increase in stress. Fretting corrosion appears as pits or grooves in the metal, surrounded by corrosion products. Damage increases with normal load on the contacting surfaces and with the amplitude of motion. There is no known minimum amplitude below which fretting stops. A decrease in pH was reported to have a negative effect on the fretting corrosion behaviour of stainless steel but no effect on CP-Ti or Co–Cr alloy [337].

**Figure 29:**
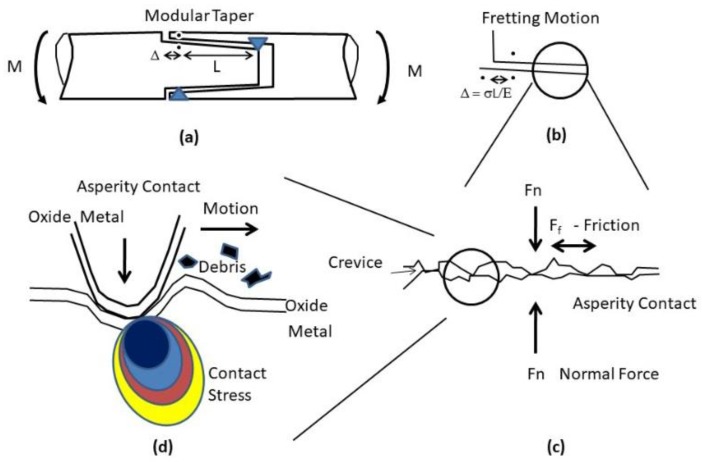
Schematics of the mechanics of fretting [40]. (**a**) Elastic bending strains under an applied bending moment, *M*, in conjunction with rigidly bound contact point (triangles) can give rise to elastically-based displacements, Δ, in the taper. (**b**) Zoom-in of elastic fretting strains with the displacement being dependent on the bending stress, elastic modulus and distance from the rigid contact. (**c**) Schematic of a zoom-in onto the contact region within a modular taper. Crevice solution can creep to the contacts and the interface will consist of asperity-asperity contact and both normal and frictional stresses. (**d**) Close-up of metal-oxide surfaces in asperity contact, causing contact stresses, local surface deformation and oxide debris generation [40]. Reprinted with permission from Springer Nature.

Gilbert and Mali [40] discussed the mechanical and electrochemical conditions in fretting corrosion. Figure 29 is a series of schematics of a modular taper assembly. Figure 29a shows a typical modular taper under the action of an applied bending moment, *M*. It is assumed that there are two points within the taper that can be regarded as rigidly connected to the opposing surface. Thus, the elastic strain associated with cyclic bending will give rise to a displacement (Δ) within the taper interface (Figure 29a,b). Fretting displacements can arise merely from elastic deformation; they do not require rigid body displacements of the implant. Materials with high Young’s modulus of elasticity such as Co–Cr alloys or tapers with larger diameters will experience less elastic-associated fretting motion. A zoom-in to the interfacial mechanics reveals that there is asperity-asperity contact that initially takes place between metal-oxide surfaces (Figure 29c,d). Within this contact zone, there is enough space for a crevice and solution to fill the space. Contact stresses and frictional interactions will arise within these regions and if the stresses and deformation mechanisms are sufficiently large, oxide disruption will occur and oxide debris will be generated [40].

**Figure 30:**
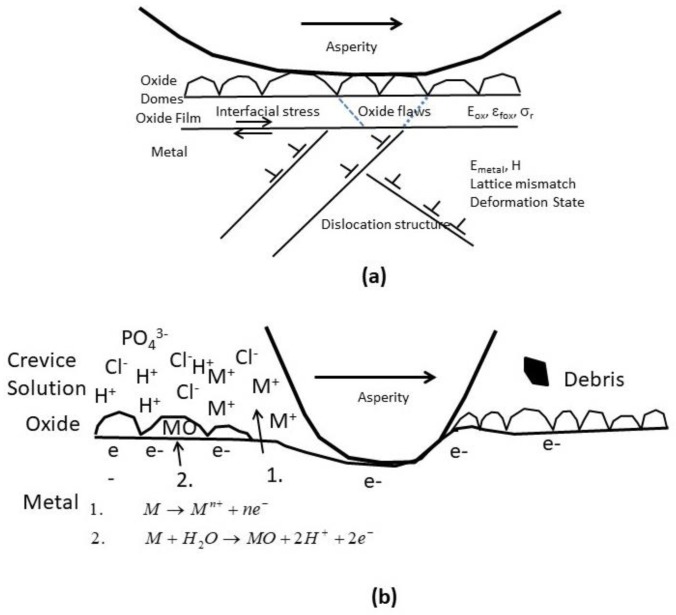
Schematics of asperity-oxide-metal interfaces showing some of the factors associated with fretting corrosion processes [40]. (**a**) The surface oxide film in contact with an opposing asperity has a structure that typically consists of an oxide dome on an oxide film with its characteristic modulus of elasticity, fracture strain and residual stresses. A lattice mismatch between oxide and metal gives rise to residual stresses and interfacial stresses as does the asperity interaction. The metal substrate structure and chemistry are also important, for example, the dislocation density. (**b**) An asperity moving across a metal-oxide surface where contact stresses and motion scrape oxide from the metal surface, generating oxide debris. Behind the moving asperity, electrochemical reactions regenerate the oxide and dissolve metal cations into the crevice solution. Hydrogen ion generation and anions migrating into the crevice to maintain charge neutrality cause local pH drop [40]. Reprinted with permission from Springer Nature.

Figure 30, also suggested by Gilbert and Mali [40], illustrates the mechanical, microstructural and electrochemical processes that take place during fretting corrosion. The nominal contact stresses necessary to induce oxide disruption sufficient to cause enhanced corrosion is an imperative parameter; it is probably very low for Co–Cr alloys and even lower for Ti alloys. The local asperity-asperity contact stresses are probably much higher; however, it is also probable that only rather low contact stresses are necessary for oxide abrasion processes. Figure 30b also shows what happens electrochemically just behind a moving asperity that is scraping oxide film from the metal surface. Oxide debris is released while metal dissolution and oxide repassivation reactions take place. Both electrochemical reactions lead to the accumulation of cations in the crevice solution and electrons on the metal surface (causing a drop in surface potential). The electrons can be transferred to any other location on the metal surface where reduction reactions can take place. In addition, anions such as Cl^−^ and $PO_{4}^{3-}$ will diffuse into the crevice in order to maintain charge neutrality and balance the cation release. This combination results in lowering of both pH and phosphate and chloride ion levels (among others) in the crevice. These reactions, if they continue within the crevice and are not terminated, will result in a highly aggressive acidic solution that may result in continued corrosion processes after the fretting motion stops. In fact, in vitro testing of modular tapers reveals this continuing process by the increased currents that remain after loading is stopped [40].

Fretting corrosion can harshly change the corrosion behaviour by mechanically destroying the protective passive film. Consequently, the ability of the material to repassivate is vital. Fretting corrosion at joints results in aseptic loosening, early loss of mechanical integrity and eventual failure of the implant, thus requiring revision surgery and causing suffer to the patients [341]. Mechanical wear at joints can lead to loss of the surrounding cement or bone, thus increasing the level of implant motion and the likelihood of corrosion fatigue [337]. In addition, accelerated release of corrosion products such as heavy-metal ions and particulate debris into the surrounding tissue could cause adverse biological reactions and reduce the biocompatibility, as discussed in Section 3.

The resistance against fretting corrosion is determined by the chemical composition and properties of both the bulk material and the surface oxide, hardness, tolerances of modular connections and the biological environment [40,337]. 316L stainless steel was reported to have better fretting corrosion behaviour than Ti–6Al–4V, while the latter along with Ti–Al–Nb have better fretting corrosion resistance than CP-Ti. The poor fretting corrosion resistance of Ti–6Al–4V (see Section 6) is a significant drawback of this alloy. In an attempt to improve its resistance, anodizing, thermal oxidation, nitride coatings and some other approaches have been explored. Higher hardness typically results in lower fretting wear [337].

Sawada et al. [350] compared the fretting corrosion behaviour in saline solution of an experimental cast titanium alloy Ti–20Cr (wt%) to that of CP-Ti. The Ti–20Cr alloy showed reduced surface damage and improved fretting corrosion resistance compared to CP-Ti. The latter exhibited severe damage and significantly higher wear depth.

A typical fretting corrosion plot is presented in Figure 34. Each maximum current peak and the baselines before and after scratching are recorded. Each activation peak (*I*_peak_) is calculated from the difference between the baseline (*I*_∞_) and the maximum current peak (*I*_max_), where the current is typically in units of μA, using Equation (30) [350]:

(30) $$I_{\mathrm{peak}}=I_{\max}-I_{\infty }.$$

**Figure 31:**
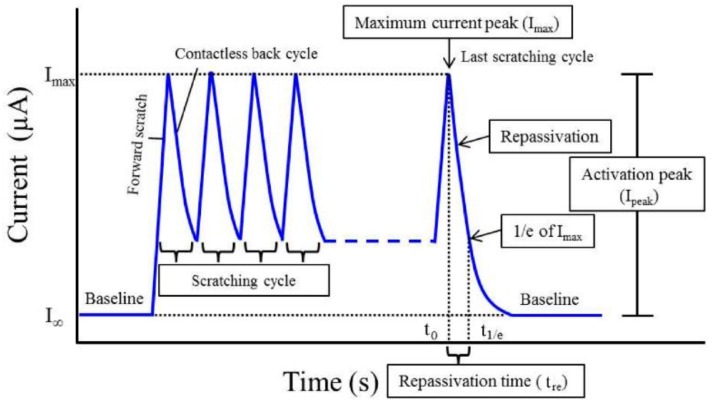
A typical fretting corrosion current transient [350]. Reprinted with permission from MDPI AG.

It is usually assumed that the passivation is completed when the baseline is reached again. Since this cannot always be reliably determined, the repassivation time (*t*_re_) can be defined as the difference between the times of the maximum current peak (*t*_0_) and 1/e of the maximum current peak (*t*_1/e_) in the last scratching cycle (see Figure 31), using Equation (31) [350]:

(31) $$t_{\mathrm{re}}=t_{1/e}-t_{0}.$$

Sivakumar et al. [346] studied the effect of surface roughness on the fretting corrosion behaviour of CP-Ti in Ringer’s solution at pH 7.3. The typical shift of potential towards more negative (cathodic) values during fretting corrosion was determined experimentally and then calculated using some empirical parameters. Celis et al. [351] explained the potential drop in terms of change in the reversible potential, *E*_rev_, that is shifted from the standard conditions of 1 atmospheric pressure and a unit ion activity when the pressure raises due to fretting contact. The material deformation caused by the fretting load is expected to alter the enthalpy and, therefore, the Gibbs free energy, which is translated into change in the electrochemical potential. In addition, hydrodynamics and change in the surface state from passive to active could both lead to drop in potential during fretting corrosion. Vieira et al. [352] analysed quantitatively the potential drop during fretting corrosion at OCP condition in terms of a galvanic couple where the area under the fretting stroke acts as an anode and the surrounding area acts as a cathode. The Tafel equation was used to calculate the potential drop. The anodic current density was used to simulate the potential drop; it was chosen arbitrarily and the best-fit value was compared with the current density obtained from anodic polarization curves.

**Figure 32:**
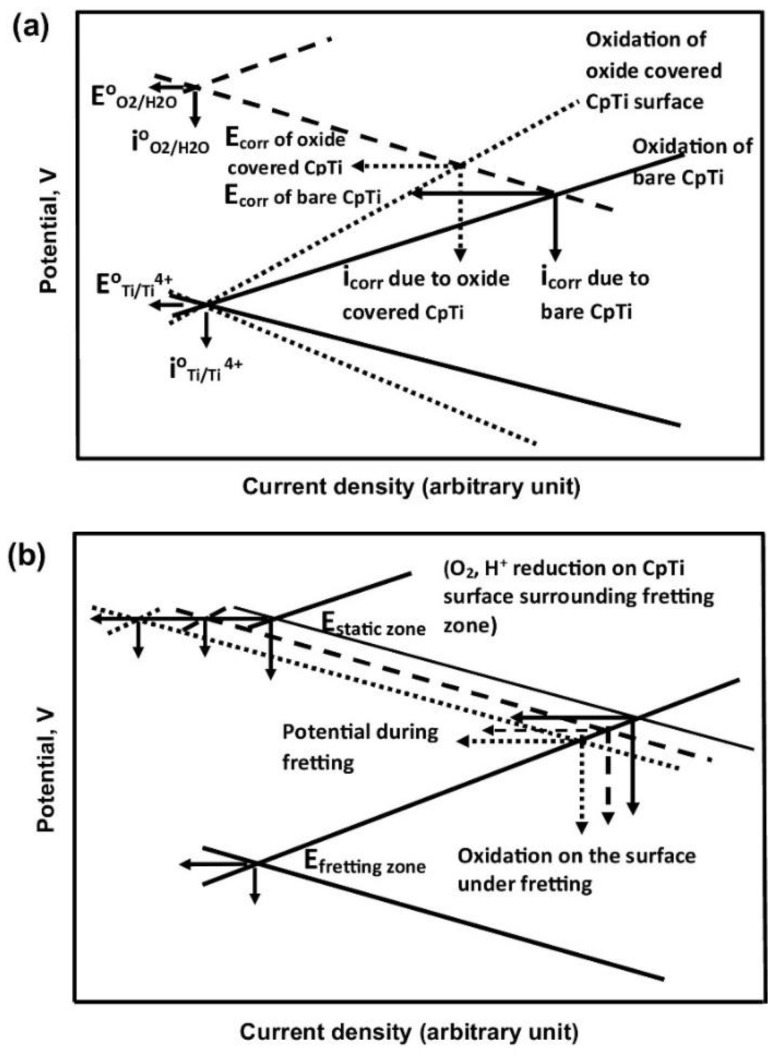
Evans diagrams illustrating the change in potential and current due to (**a**) the oxide covered Ti surface, and (**b**) the increase in fretting area/time. Dashed lines represent the changes occurring as the cathode area decreases with the progress in fretting [346]. Reprinted with permission of Elsevier.

Sivakumar et al. [346] used Evans diagrams to describe the potential drop during fretting corrosion of CP-Ti in Ringer’s solution, see Figure 32. Figure 32a shows increase in OCP towards more positive values and decrease in corrosion current density as a result of the higher Tafel slopes of the oxide-covered CP-Ti. The negative drop in the OCP during fretting corrosion is manifested by the removal of existing passive oxide film formed during the fretting ‘OFF’ and exposure of the fresh CP-Ti surface to the Ringer’s solution. The maximum drop in potential due to the fretting corrosion is controlled by the oxidation of titanium and reduction of oxygen in the Ringer’s solution. The value of the corrosion potential thus obtained lies between the values for oxygen reduction and titanium oxidation according to the mixed-potential theory. The corrosion potential will be influenced further by the kinetic parameters associated with the chemical species in the solution that form oxides. As soon as fretting disrupts the passive film, the corroding system becomes galvanic, with small anode under the fretting contact and large cathode outside the fretting zone, establishing potential difference on the surface that accelerates corrosion. This is illustrated in Figure 32b. As fretting progresses, the anodic area broadens, therefore the potential further decreases to more negative values. The decrease in absolute potential drop with the increase in the fretting area/time is evident in Figure 32b.

**Figure 33:**
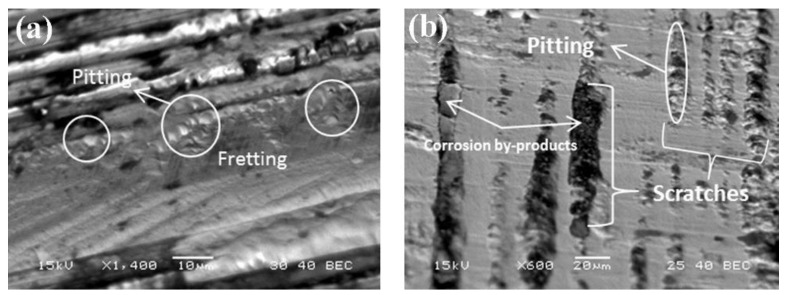
(**a**) The metal taper of a retrieved hip implant revealing pitting corrosion (marked in white circles) initiated preferentially in a crevice formed due to fretting abrasion (5–40 μm scratches). This damage was imaged midway between proximal and distal ends on the taper. (**b**)A different component showing scratches (50–500 μm) throughout head taper, with preferential pitting inside the scratches, imaged midway between the proximal and distal ends on the taper. Corrosion by-products (biological and electrochemical deposits) accumulated inside the scratches [353]. Reprinted with permission of Elsevier.

Figure 33 [353] shows typical fretting corrosion features at the metal taper of a retrieved hip implant. Pitting preferentially evolved in regions that showed evidence of fretting (scratches in the scale of 5–40 μm) or had larger scratches (50–500 μm). It was hypothesized that the larger scratches were formed when the head was impacted onto the trunnion during the primary surgery or during removal. 

## 8. Strategies for Corrosion Control In Vivo

As discussed in the previous sections, corrosion of biomaterials in vivo rises two major concerns: (1) its effect on the functionality and lifetime of the medical device, and (2) possible adverse effects on biocompatibility (e.g., hypersensitivity and caner). Ion toxicity levels may be attained even at corrosion rates that are insignificant with respect to the physical performance of the implant. 

Most strategies commonly used in industry to mitigate corrosion are not applicable in the body environment. These include: (1) changing the chemistry of the environment, (2) controlling the oxygen level, (3) adding inhibitors, (4) changing the pH, (5) lowering the temperature, and (6) applying anodic or cathodic protection. In addition, service in vivo raises unique challenges due to the negative effects of proteins, enzymes and other biological matter on corrosion processes and the action of wear and high loads on load-bearing implants. Hence, corrosion control in vivo is currently limited mainly to careful design (e.g., to prevent galvanic couples or crevices), proper material selection, and surface modification. From Section 5 it could be realized that the list of potential materials is currently somehow limited because of mechanical and biocompatibility requirements. Therefore, much attention has been paid to surface modification approaches [6,8,33,37,39,354,355,356,357,358]. Yet, coatings are of limited use because many of them may be subjected to wear in vivo. This makes proper material selection during implant design exceptionally important. 

A new challenge that will confront corrosion scientists in the near future stems from the introduction of additively manufactured (AM) or 3D-printed dental and other implants. 3D printed metals are usually more porous, less homogeneous and possibly contain higher residual stresses than the wrought counterpart metal. Similarly, AM cellular and other structures often contain inherently more crevices. Therefore, these advanced materials and implants might be more prone to corrosion.

Some processes for surface modification of implants include laser-induced surface modification, ion implantation, laser melting, laser alloying, sintering, plasma spraying (PS), pulsed laser deposition (PLD), sputtering, physical or chemical vapor deposition (PVD or CVD, respectively), precipitation from solution, sol-gel wet chemistry, electrophoretic deposition (EPD), electrodeposition and surface texturing.

HAp and other CaP coatings provide enhanced osseointegration and fixation of orthopedic and dental implants [7,8,9,10,11,12,13,14,15,16,17,18,19,20,21,22,23,24,25,26,27,28,29,30,31,32]. In spite of the porous structure of many of these bioceramic coatings, it was reported that the corrosion resistance of CP-Ti could be improved by electrodepositing it with HAp, as long as the deposition conditions did not result in a too porous coating [12]. 

**Figure 34:**
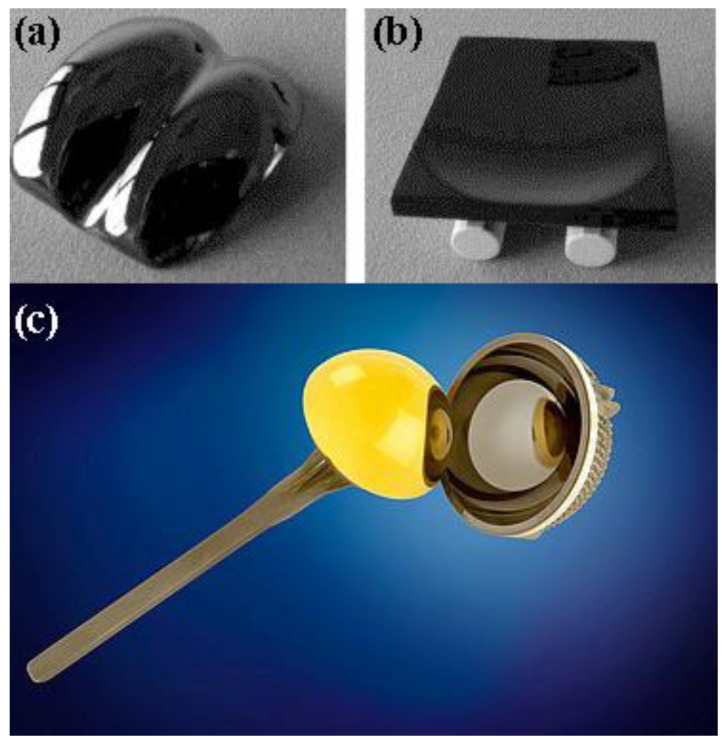
DLC-coated artificial joints. (**a**) Talar and (**b**) tibial components of an ankle joint, both made from a nitrided AISI Z5 CNMD 21 steel and coated with DLC. Manufacturer: Matériels Implants du Limousin SA (M.I.L.SA.) [360]. Reprinted with permission of Elsevier. (**c**) Diamolith™ (DLC)-coated acetabular cup in a hip joint. Diamolith™ is deposited by Plasma Enhanced Chemical Vapour Deposition (PECVD). It is deposited by a vacuum-based process where precursor gases are introduced into the process chamber and are broken down within the ionized plasma into various species of carbon, hydrogen and other dopants that are subsequently condensed as a solid entity onto the substrate surface. Source: http://www.dpaonthenet.net/article/55077/Diamond-like-coatings-service-prolongs-tool-and-component-life.aspx.

Diamond-like carbon (DLC) coatings have emerged during the last 20 years as promising materials for biomedical applications [359,360,361,362,363,364,365,366,367,368]. DLC, also known as amorphous hydrogenated carbon (a-C:H), is a class of materials with excellent mechanical, tribological and biological properties. By the addition of other elements to the DLC, all of these properties can be tailored within a certain range. DLC also has excellent corrosion resistance. DLC coatings have excellent haemocompatibility, which is expressed in a decreased thrombus formation. When exposed to blood, an increased ratio of albumin to fibrinogen adsorption as well as decreased platelet activation is observed on coated surfaces. Hence, DLC-coated cardiovascular implants such as artificial heart valves and stents are already commercially available. On the hand, findings from both in vitro and in vivo tests on the suitability of DLC-coated bearing surfaces of joint replacement remain inconclusive [360,363,365]. Figure 34 shows artificial ankle and hip joints coated with DLC.

When stainless steel products are manufactured, free iron is transferred to the surface of the material from the steel cutting, stamping and forming tools used in the manufacturing process. Free iron can also be imparted on the surface by polishing or blasting operations that utilize the same polish or blast media between both mild steel and corrosion resistant steel grades. Free iron readily oxidizes, forming visible rust on the surface of the product. *Passivation* of stainless steels is a chemical treatment with a specific acid formulation that removes free iron or other surface contamination from the stainless steel while simultaneously promoting the formation of a passive layer. When the surface iron is removed, the other components of the alloy (primarily chromium, often nickel as well) are left behind as a surface layer over the underlying steel. Upon exposure to air, they react with oxygen to form an oxide layer that protects the rest of the steel from corrosion. Passivation treatment usually consists of cleaning the stainless steel and then immersing it in a concentrated HNO_3_ solution (in the case of stainless steels that contain more than 17 wt.% Cr) or in a solution of HNO_3_ with oxidizing salts (in the case of stainless steels for machining, polished surfaces, or stainless steels with Cr-content lower than 17 wt.%). This process forms a transparent passive layer, typically about 3 nm thick. Applicable standards include ASTM A380, A967 and F86, AMS 2700, ISO 16048 and QQ-P-35 specifications. Passivation of high-strength steels should be followed by hydrogen release [46].

**Figure 35:**
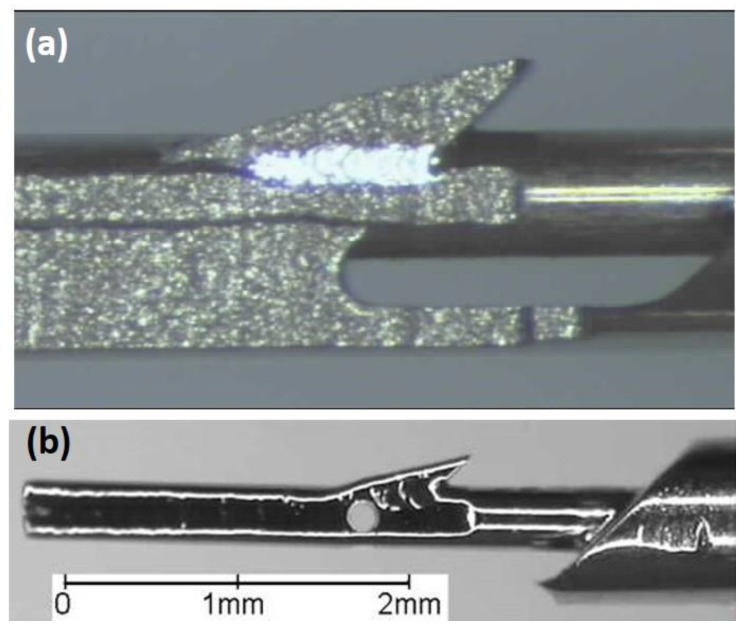
A miniature glaucoma implant (Ex-PRESS™, Optonol Ltd.) made of 316LVM steel. (**a**) A close-up after mechanical grinding, and (**b**) A device after novel electropolishing [369]. Reprinted with permission of John Wiley and Sons.

The corrosion performance of medical devices made of stainless steels can also be improved by electrochemical polishing (*electropolishing*) [369,370]. In this process, the part to be polished is immersed in an electrolyte and, while an electric current (or voltage) is applied, a thin metal layer is removed. Thus, the surface becomes smooth, free of contaminants and internal stresses and more passive. The improvement in corrosion resistance may be attributed to preferred dissolution of Fe and Ni, thereby forming a Cr-rich oxide layer. The decreased surface roughness results in an increased resistance against bacteria growth and a reduced protein adsorption, which is helpful in preventing ingrowth of tissue and complications (e.g., restenosis in stent applications). Several ASTM standards report on passivation of stainless steels and cobalt-based alloys by electropolishing. According to ASTM F 86 [371], one can omit the nitric acid rinse (i.e., chemical passivation) requirement if electropolishing is conducted as the final processing stage, since it is recognized that electropolishing is a satisfactory passivation treatment. Figure 35 shows an example of a commercial medical device from Optonol Ltd., made of 316LVM stainless steel. The fabrication of this miniature glaucoma implant, called Ex-PRESS™, involves processes such as electro-erosion (EDM) and laser welding. Due to the complex geometry and surface inhomogeneities (both chemical and microstructural) resulting from the fabrication processes, it is very difficult to achieve good results using the standard electropolishing process. Therefore, delegate mechanical polishing (each implant separately) was used in the past. However, following the development of an efficient non-standard electropolishing process [369], this medical device is nowadays electropolished automatically. It is anticipated that further improvements in this area will allow polishing of other miniature implants as well, thus improving their performance in vivo. Good electropolishing will be required and is currently still a challenge, also for obtaining good surface finish of 3D-printed parts.

A broadly studied method for optimizing the surface morphology of Ti implants is spark-anodizing [372,373,374,375,376]. The corrosion behaviour of anodized Ti is typically enhanced significantly in comparison to a machined or polished Ti surface due to the presence of a thick oxide layer on the surface [38].

Nitrogen ion implantation is another way of surface modification of medical devices, mainly made of stainless steels. Surface barriers and coatings often have only limited use in protecting implants due to their abrasion and wear, especially in orthopedic applications. Ion implantation is a versatile surface alloying technique, which produces novel metastable solid-solution surface alloys of no composition limits as those normally imposed by equilibrium phase diagrams. Nitrogen ion implantation can be carried out on finished orthopedic devices because the process does not introduce any dimensional changes at the surface after implantation. In addition to corrosion resistance, it also imparts excellent wear resistance to the modified surfaces [37].

Maurer et al. [377] evaluated the reduction of fretting corrosion of Ti–6Al–4V by ion implantation, PVD nitriding and plasma ion nitriding. The latter was found to have the most significant beneficial effect, with the weight loss and titanium release being only 11% and 2% of the control values, respectively.

Shot blasting is commonly used to introduce compressive stresses to the surface, increase the surface area and improve the adhesion of coatings to substrates. Aparicio et al. [378] compared between the corrosion resistance of CP-Ti blasted with either SiC or Al_2_O_3_ of various particle sizes. The differences found in the electrochemical behaviour and corrosion resistance were attributed not only to the increased surface area but also to the compressive residual surface stresses induced by shot blasting. CP-Ti blasted with SiC showed significantly higher current densities in cyclic polarization tests compared to CP-Ti blasted with Al_2_O_3_.

Laser processing is used to produce a high degree of purity with sufficient roughness for good osseointegration [379]. It was also found to significantly improve the corrosion resistance of Ti–6Al–4V alloy [380].

Surface biofunctionalization of metals and other biomaterials has been studied extensively [20,381]. Metoki et al. [382,383] investigated both active (electroassisted) and passive (adsorption) approaches for surface modification of Ti–6Al–4V using alkylphosphonic acid self-assembled monolayers (SAMs) with different chain lengths. The electrosorbed SAMs were found to exhibit better blockage of electron transfer across the interface and thus had better corrosion resistance. Nevertheless, because the main purpose of surface biofunctionalization is usually not to impair corrosion resistance, even if on the way it does, it will not be discussed here in more detail.

With respect to proper materials selection, surgeons and implant manufacturers should pay attention not to bring in contact different grades of stainless steels (or stainless steels with other alloys) because this might result in galvanic corrosion. As an example, Jedwab et al. [384] reported failure when just one of a group of screws holding a 316L fracture plate in position was fabricated from the lower grade 304L.

The corrosion behaviour of porous metals (e.g., Ti foams and trabecular metals) should be well studied as one can anticipate higher corrosion rates in inherent crevices in such materials [186,195,385,386,387,388,389]. Since the studies reported in the literature usually compare a specifically porosified material with a bulk material, the results are not consistent and do not lead to a generic, mechanistic understanding of the behaviour of porous surfaces and porous materials. Thus, a separation between size-effects and surface reactivity effects still needs to be elucidated [38].

Implant manufacturers should seek for the best design practices in order to minimize porosity, inherent pits and crevices, and inclusions [390]. Improved alloy ’cleanliness,’ especially the use of vacuum melting and re-melting, has been claimed to eliminate pitting in such medical devices [391]. One should refrain from implanting different metals in the same region. In the manufacturing process, matched parts from the same batch of the same variant of a given alloy must be provided. It must be ensured that instruments are made from the same material as the implant. The strongest practices of quality control should be assured. Corrosion might result from improper materials selection, wrong metallurgical condition or from the supply of material which is off-specification [392]. Always keep in mind that a metal that resists corrosion in one body environment might corrode in another part of the body [390].

The practice of steam sterilization of implants with saline in the environment gives rise to surface corrosion of stainless steels in both instruments and implants and should be forbidden. Mixed metal sterilization in the same basket might also cause corrosion. Both spotting and staining do not portray sterility. Flash sterilization can also damage passive layers eventually. This happens mainly because of the rapid temperature change that the surface experiences during flash sterilization. Implants should be handled with delicacy, never thrown around in basins or shaken together in a basket or immersed in saline. Implants should be kept in their packages or placed in protective containers until the time of use. Implants become seriously attenuated after being subjected to repeated stresses in the hostile body environment and are much more liable to fail if reused [392]. Avoid transfer of metal from tools to the implant or tissue. For example, drill guides should be used to prevent contact between drill and plate [392]. Poor assembly of multi-component implants might lead to crevice corrosion and should thus be avoided.

## 9. Conclusions

Metallic biomaterials represent the class of materials with the largest use in medical devices in humans today. Metals and alloys have a large range of biomedical applications, including devices for fracture fixation, partial and total joint replacement, external splints, craniofacial plates and screws, braces and traction apparatus, dental amalgams, cardiovascular surgery and so forth. The most common metallic biomaterials are reviewed in this article, with focus on their corrosion performance in vitro and in vivo. These biomaterials include stainless steels, cobalt-chromium alloys, titanium and its alloys, Nitinol shape memory alloy, dental amalgams, gold, metallic glasses and biodegradable metals. The importance of passivity, its breakdown and regeneration is highlighted. Corrosion is an important factor in the design and selection of metals and alloys for service in vivo. The corrosion resistance of an implant material affects its functionality and durability and is a primary factor governing biocompatibility. Ion toxicity levels, possibly leading to hypersensitivity and caner, may be attained even at corrosion rates that are insignificant with respect to the physical performance of the implant. From the perspective of corrosion, the most important characteristics of body fluids are the chloride, dissolved oxygen and pH levels. Different parts of the body have different pH values and oxygen concentrations. Consequently, a metal which performs well in one part of the body may suffer an unacceptable amount of corrosion in another part. Biological macromolecules can influence the rate of corrosion by interfering in different ways with the anodic or cathodic reactions. When combined with mechanics (static loading, dynamic loading or wear), restricted crevice-like geometries, inflammation or any combination thereof, corrosion is intensified. Failures of implants are usually classified as either mechanical, electrochemical, biological or combinations of these. The corrosion behaviour of a metal in non-physiological in vitro studies versus physiological in vitro studies versus in vivo studies may vary dramatically. The forms of corrosion most typical of implants and other medical devices include localized (both pitting and crevice), intergranular, dealloying and galvanic corrosion, as well as stress corrosion cracking, corrosion fatigue and fretting corrosion. Most practices commonly used in industry to mitigate corrosion are not applicable in the body environment. Therefore, corrosion control in vivo is currently limited to careful design, proper material selection and surface modification. The effectiveness of coatings may be limited in vivo due to wear. Advanced 3D-printed metal implants raise special concerns regarding their corrosion resistance. 

## Figures and Tables

**Table 1:** Compositions of various body fluids [55].

Component	Interstitial Fluid (mg·L^–1^)	Synovial Fluid (mg·L^–1^)	Serum (mg·L^–1^)
Na^+^	3280	3127	3265
K^+^	156	156	156
Ca^2+^	100	60	100
Mg^2+^	24	---	24
Cl^-^	4042	3811	3581
HCO_3_^-^	1892	1880	1648
HPO_4_^2-^	96	96	96
SO_4_^2-^	48	48	48
Organic acids	245	---	210
Protein	4144	15,000	66,300

**Table 2:** Compositions of various simulated body fluids (SBFs) [55].

Component	PBS (g·L^–1^)	Ringer’s (g·L^–1^)	Hank’s (g·L^–1^)
NaCl	8.00	8.60	8.00
CaCl_2_	---	0.33	0.14
KCl	0.20	0.30	0.40
MgCl_2_·6H_2_O	---	---	0.10
MgSO_4_·7H_2_O	---	---	0.10
NaHCO_3_	---	---	0.35
NaH_2_PO_4_	1.15	---	---
Na_2_HPO_4_·12H_2_O	---	---	0.12
KH_2_PO_4_	0.20	---	0.06
Phenol red	---	---	0.02
Glucose	---	---	1.00

**Table 3:** The biological effects of certain metal ions that may be leached in vivo due to corrosion.

Metal	Effects
Nickel (Ni)	The major cause of allergic contact dermatitis. The significant biological parameter is the amount released to the skin during exposure to human sweat. Threshold: 0.5 mg cm^–2^ week^–1^, at which only a minor part of Ni-sensitive subjects will react. Has toxic effects with cellular damage in cell cultures at high concentrations. Harmful to bone in tissue cultures, although less than Co or V. Has a potency for carcinogenicity [75]. A normal blood level of Ni is about 5 mg L^–1^ [73]
Cobalt (Co)	Its function is confined to its role in vitamin B12 [73]. Anaemia B inhibiting Fe absorption into the blood stream [79]
Chromium (Cr)	Ulcers and central nerve system disturbances [79]. Blood level average: 2.8 μg/100 g). Its compounds are only poorly absorbed after oral ingestion and storage of Cr(III) is largely confined to the reticuloendothelial systems. Cr(VI) ion is able to pass the plasma membrane freely in both directions [73]
Aluminium (Al)	Epileptic effects and Alzheimer’s disease [79]
Vanadium (V)	Toxic in the elementary state [79]
Molybdenum (Mo)	An essential dietary element. Its highest concentration in the liver: 1–3 ppm. Is necessary for the function of certain enzymes. Is quite readily absorbed from the intestinal tract. Is toxic in large doses; symptoms include diarrhoea, coma and cardiac failure and inhibition of activity of ceruloplasmin, cytochrome oxidase, glutaminase, choline esterase and sulphite oxidase. High levels of Mo can also interfere with Ca and P metabolism [73]

**Table 4:** Analysis of the surface oxide film on various metallic biomaterials.

Biomaterial	Surface Oxides	Surface Analysis
CP-Ti	Ti^0^, Ti^2+^, Ti^3+^, Ti^4+^	Ti^2+^ oxide thermodynamically less favourable than Ti^3+^ formation at the surface. Ti^2+^ and Ti^3+^ oxidation process proceeds to the uppermost part of the surface film and Ti^4+^ is observed on the surface outer most layer
Ti–6Al–4V	TiO_2_	Surface consists of small amount of Al_2_O_3_, hydroxyl groups and bound water. The alloying element V is not detected
Ni–Ti	TiO_2_-based oxide	Minimal amounts of Ni in both oxide and metal states
Ti–56Ni	TiO_2_	Very low concentrations of metallic Ni, NiO, hydroxyl groups and bound water
Ti–Zr	Ti and Zr oxides	Ti and Zr are uniformly distributed along the depth direction. The thickness of the oxide film increases with increase in Zr content
316L stainless steel	Oxides of Fe, Cr, Ni, Mo and Mn	Thickness ~3.6 nm. The surface film contains a large amount of (OH)^–^, that is, it is either hydrate or oxyhydroxide. The outer surface layer is enriched with Fe, beneath is it enriched with Ni, Mo and Mn
Co–36.7Cr–4.6Mo alloy	Oxides of Co and Cr, without Mo	Thickness ~2.5 nm. The surface film contains a large amount of (OH)^–^, that is, it is either hydrate or oxyhydroxide. Cr and Mo are distributed more in the inner layer of the film

**Table 5:** Chemical compositions of stainless steels and cobalt-based alloys currently (or potentially) used as biomaterials.

Alloy	Fe	Co	W	Al	Cr	Ni	Mo	Mn	Si	C	P	S	N	Nb	Ti
UNS S31673	Bal.	---	---	---	17.00–19.00	13.00–15.00	2.25–3.00	max 2.00	max 0.75	max 0.03	max 0.025	max 0.01	max 0.10	---	---
UNS S31675	Bal.	---	---	---	19.50–22.00	9.00–11.00	2.00–3.00	2.00–4.25	max 0.75	max 0.08	max 0.025	max 0.01	0.25–0.50	0.25–0.80	---
UNS S31703	Bal.	---	---	---	18.00–20.00	11.00–15.00	3.00–4.00	max 2.00	max 0.75	max 0.03	max 0.045	max 0.03	max 0.10	---	---
UNS S31803	Bal.	---	---	---	21.00–23.00	4.50–6.50	2.50–3.50	max 2.00	max 1.00	max 0.03	max 0.03	max 0.02	0.08–0.20	---	---
UNS S44660	Bal.	---	---	---	25.00–28.00	1.00–3.50	3.00–4.00	max 1.00	max 1.00	max 0.03	max 0.04	max 0.03	max 0.04	(Ti + Nb) = 0.20–1.00 and 6 × (C + N) min	
UNS S29108	Bal.	---	---	---	19.00–23.00	max 0.05	0.50–1.50	21.00–24.00	max 0.75	max 0.08	max 0.03	max 0.01	0.85–1.10	---	---
UNS R30075	max 0.75	Bal.	max 0.20	max 0.10	27.00–30.00	max 0.50	5.00–7.00	max 1.00	max 1.00	max 0.35	max 0.02	max 0.01	max 0.25	---	max 0.10
UNS R30605	max 3.00	Bal.	14.00–16.00	---	19.00–21.00	9.00–11.00	---	1.00-2.00	max 0.40	0.05–0.15	max 0.04	max 0.03	---	---	---
UNS R30035	max 1.00	Bal.	---	---	19.00–21.00	33.00–37.00	9.00–10.50	max 0.15	max 0.15	max 0.025	max 0.015	max 0.01	---	---	max 1.00
UNS R30003	Bal.	39.00–41.00	---	---	19.00–21.00	14.00–16.00	6.00–8.00	1.50–2.50	max 1.20	max 0.15	max 0.015	max 0.015	---	---	---
UNS R31537	max 0.75	Bal.	---	---	26.00–30.00	max 1.00	5.00–7.00	max 1.00	max 1.00	max 0.14	---	---	max 0.25	---	---

**Table 6:** Chemical compositions of titanium and its alloys currently in use as biomaterials.

	Ti	Al	V	Ta	Nb	Mo	Zr	O	N	C	H	Fe	Ni	Co	Cu
UNS R50250	Bal.	---	---	---	---	---	---	max 0.18	max 0.03	max 0.08	max 0.015	max 0.20	---	---	---
UNS R50400	Bal.	---	---	---	---	---	---	max 0.25	max 0.03	max 0.08	max 0.015	max 0.30	---	---	---
UNS R50550	Bal.	---	---	---	---	---	---	max 0.35	max 0.05	max 0.08	max 0.015	max 0.30	---	---	---
UNS R50700	Bal.	---	---	---	---	---	---	max 0.40	max 0.05	max 0.08	max 0.015	max 0.50	---	---	---
UNS R56401	Bal.	5.50–6.50	3.50–4.50	---	---	---	---	max 0.13	max 0.05	max 0.08	max 0.012	max 0.25	---	---	---
UNS R58130	Bal.	---	---	---	12.50–14.0	---	12.50–14.0	max 0.15	max 0.05	max 0.08	max 0.012	max 0.25	---	---	---
UNS R56700	Bal.	5.50–6.50	---	max 0.50	6.50–7.50	---	---	max 0.20	max 0.05	max 0.08	max 0.009	max 0.25	---	---	---
UNS R58120	Bal.	---	---	---	---	10.0–13.0	5.0–7.0	0.008–0.28	max 0.05	max 0.05	max 0.02	1.5–2.5	---	---	---
NiTi	Bal.	---	---	---	max 0.025	---	---	max 0.05	---	max 0.07	max 0.005	max 0.05	54.5–57.0	max 0.05	max 0.01

**Table 7:** Mechanical properties of selected biomaterials.

Material	UNS Designation	*σ*_UTS_ (MPa)	*σ*_YP_ (MPa)	*E* (GPa)	*ε* (%)
316L, annealed	S31673	min 490	min 190	190	min 40
316L, cold worked	S31673	min 860	min 690	190	min 10
BioDur^®^ 108 stainless steel, annealed	S29108	min 827	min 517	--	min 30
Co-28Cr-6Mo, as-cast	R30075	min 655	min 450	210	min 8
Co-28Cr-6Mo, forged	R31537	min 1172	min 827	210	min 12
Co-20Cr-15W-10Ni, annealed	R30605	min 860	min 310	210	min 30
Co-35Ni-20Cr-10Mo, solution annealed	R30035	793–1000	241–448	232	min 50
CP-Ti, grade 1	R50250	min 240	min 170	110	min 24
CP-Ti, grade 4	R50700	min 550	min 483	110	min 15
Ti–6Al–4V ELI, annealed	R56401	min 825	min 760	116	min 8
Ti–6Al–7Nb, annealed	R56700	min 900	min 800	114	min 10
Ti–13Nb–13Zr, capability aged	R58130	min 860	min 725	75	min 8
Ti–12Mo–6Zr–2Fe, solution-annealed	R58120	min 932	min 897	74–85	min 12
Ni–Ti, annealed	--	min 551	--	40	min 10
Cortical bone	--	70–150	30–70	10–30	0–8
